# The synthesis of imidazo[1,5-*a*]quinolines *via* a decarboxylative cyclization under metal-free conditions†

**DOI:** 10.1039/c8ra03786h

**Published:** 2018-06-25

**Authors:** Zicong Yan, Changfeng Wan, Yu Yang, Zhenggen Zha, Zhiyong Wang

**Affiliations:** Hefei National Laboratory for Physical Sciences at Microscale, CAS Key Laboratory of Soft Matter Chemistry & Center for Excellence in Molecular Synthesis of Chinese Academy of Sciences, Collaborative Innovation Center of Suzhou Nano Science and Technology & School of Chemistry and Materials Science, University of Science and Technology of China 230026 Hefei Anhui P. R. China zwang3@ustc.edu.cn +86-551-6360-3185; College of Chemistry and Chemical Engineering, Jiangxi Normal University 330022 Nanchang P. R. China wanfeng@jxnu.edu.cn

## Abstract

An iodine-mediated decarboxylative cyclization was developed from α-amino acids and 2-methyl quinolines under metal-free conditions, affording a variety of imidazo[1,5-*a*]quinolines with moderate to good yields.

## Graphical abstract

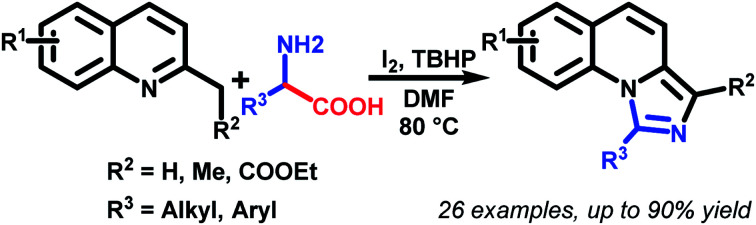

## Introduction

**Scheme 1:**
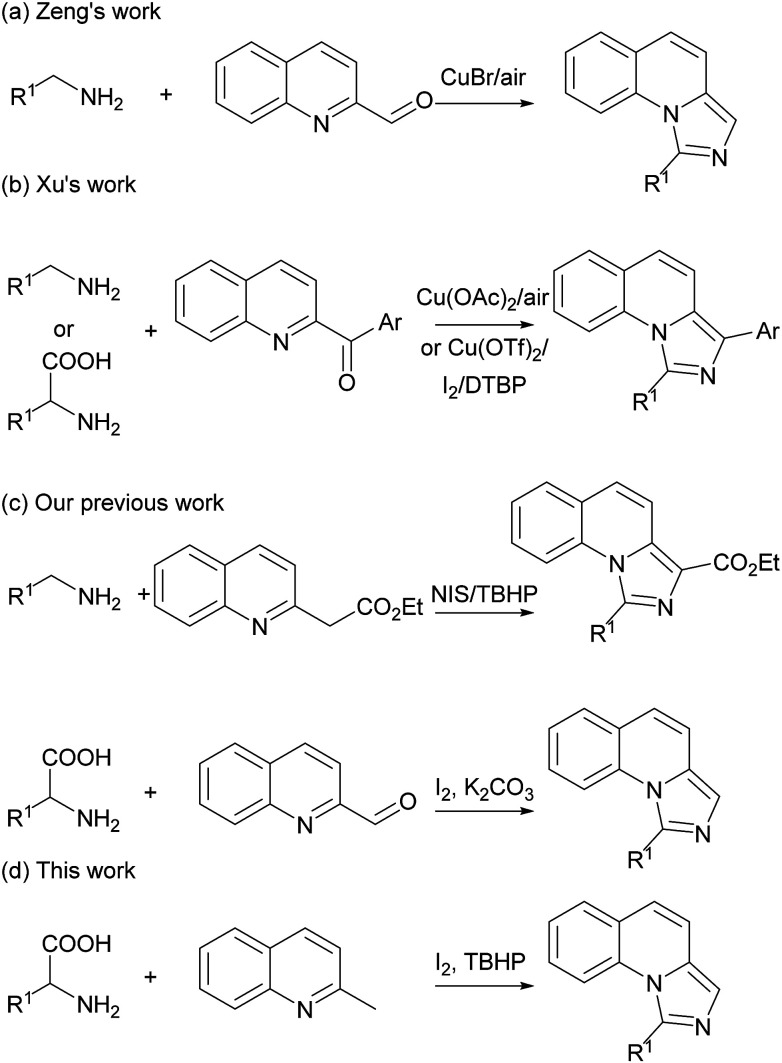
Previous study and this work for the synthesis of imidazo[1,5-*a*]quinolines.

The imidazo[1,5-*a*]quinoline moiety is an important class of fused *N*-heterocyclic compounds such as skeletons of NK1 receptor ligands,^1^ antitumor drug C-1311,^2^ potential antimicrobial natural product cribrostatin 6,^3^ inhibitor targeting phosphodiesterase 10A^4^ and highly efficient ligands of central benzodiazepine receptors.^5^ As the structure for the precursors of *N*-heterocyclic carbene^6^ and other transformations,^7^ much effort has been done for the synthesis of imidazo[1,5-*a*]quinolines. The previous strategies mainly focused in Vilsmeier-type cyclizations using *N*-2-pyridylmethylamides as starting materials.^8^ Subsequently, various new protocols have also been developed to synthesize these compounds.^9^ Recently, Zeng and Xu groups independently reported copper-catalyzed imidazole synthesis *via* the oxidative C–H amination reactions (Scheme 1a and b).^10^ These elegant protocols led to new reactivity pathways and made great progress in imidazole synthesis. However, these reported methods still suffered from the usage of transition-metal catalysts. Our group also developed previously some oxidative C–H amination reactions for imidazole synthesis under metal-free conditions.^11^ The main drawbacks are the narrow scope of the substrates and the difficulty of the available starting materials. Therefore, a more environmental-friendly methodology with readily available starting materials for the preparation of imidazo[1,5-*a*]quinolines is also highly desirable.

On the other hand, decarboxylative reactions were widely used in organic synthesis, especially in pericyclic reactions for heterocycles.^12^ Recently, a series of cascade decarboxylative reactions involving azomethine ylides have been developed to construct C–C and C–N bonds.^13^ As a facile, stable and cheap starting materials, amino acids has long been neglected in organic synthesis.^14^ Our group also developed some decarboxylative cyclization reactions from amino acids.^15^ As our continuing interest in the decarboxylative reaction for the synthesis of *N*-heterocycles, herein we report a new decarboxylative cascade reaction for the synthesis of imidazo[1,5-*a*]quinolines starting from readily available materials under metal-free condition (Scheme 1c and d).

## Results and discussion

Initially, we employed 2-methylquinoline (1a) and valine (2a) as model substrates. The results are summarized in Table 1. Firstly, the reaction of 1 equiv. of 1a and 1.5 equiv. of 2a was carried out in the presence of 1 equiv. of I_2_ in *N*,*N*-dimethylformamide (DMF) at 80 °C for 3 h to give rise to a 20% yield of the expected product 1-isopropylimidazo[1,5-*a*]quinoline (3aa) (Entry 1, Table 1). Then the influence of oxidants on this reaction was investigated (Entries 2–6, Table 1). It was found that the yield could be improved to 84% by using 3 equiv. of *tert*-butyl hydroperoxide (TBHP) while the other oxidants, such as di-*tert*-butyl peroxide (DTBP), dioxygen and peroxysulfates had little influence on the reaction or deteriorated the reaction.

**Table 1:** Optimization of reaction conditions^a^

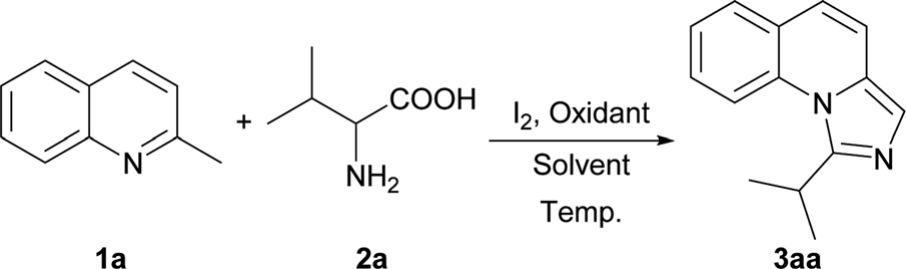				
Entry	Oxidant	Solvent	Temp. (°C)	Yield^b^ (%)
1	—	DMF	80	20
2	TBHP	DMF	80	84
3	O_2_	DMF	80	84
4	DTBP	DMF	80	18
5	K_2_S_2_O_8_	DMF	80	0
6	(NH_4_)_2_S_2_O_8_	DMF	80	Trace
7	TBHP	DMA	80	81
8	TBHP	DMSO	80	57
9	TBHP	H_2_O	80	n. d.
10	TBHP	Tol.	80	n. d.
11	TBHP	MeCN	80	Trace
12	TBHP	MeOH	80	n. d.
13	TBHP	THF	80	n. d.
14	TBHP	1,4-Dioxane	80	n. d.
15	TBHP	DMF/H_2_O	80	37
16	TBHP	DMF/MeOH	80	49
17	TBHP	DMF	25	25
18	TBHP	DMF	50	43
19	TBHP	DMF	100	83
20	TBHP	DMF	120	83
21^c^	TBHP	DMF	80	0
22^d^	TBHP	DMF	80	17

a Reaction conditions: 1a (1.0 equiv., 0.2 mmol), 2a (1.5 equiv., 0.3 mmol), I_2_ (1.0 equiv.), oxidant (3 equiv.) in solvent (0.5 mL).

b Isolated yield.

c The reaction was carried out without I_2_.

d The reaction was carried out with 20% of I_2_.

Afterwards several solvents were also examined (Entries 7–16, Table 1). *N*,*N*-Dimethylacetamide (DMA) had a little negative influence on the reaction (Entry 7, Table 1) while dimethyl sulfoxide (DMSO) reduced the reaction yield remarkably. Other solvents, such as ethyl nitrile, toluene, tetrahydrofuran (THF), methanol, 1,4-dioxane and water would result in the failure of the reaction (Entries 9–14, Table 1). The mixed solvents deteriorated the reaction either (Entries 15–16, Table 1). Subsequently the different temperature was examined for this reaction. When the reaction temperature was increased to 100 °C, the corresponding product can be obtained with a yield of 83%. (Entry 19, Table 1). Further increase of the temperature could not improve the yield (Entry 20, Table 1). The product 3aa was obtained with the yields of 25% and 43% when the reactions were carried out at 25 °C and 50 °C, respectively (Entry 17–18, Table 1). This indicated that reducing the reaction temperature would destroy this reaction. The reaction could not proceed without I_2_ and the yield of the product decreased to 17% while only 20% of the I_2_ was added (Entries 21–22, Table 1). Finally, the optimized reaction conditions were obtained as described in entry 2 of Table 1: 1.0 equiv. of 2-methyl quinoline 1a and 1.5 equiv. of α-amino acid 2a as the reaction substrates, 1.0 equiv. of iodine and 3.0 equiv. of TBHP as the oxidants, the reaction being carried out in 0.5 mL of DMF at 80 °C for 3 h.

With the optimized conditions in hand, we explored the scope of the reaction substrates. Firstly, different substrates with groups on R^1^ and R^2^ were examined, and the results were listed in Table 2. Generally, 6-substituted 2-methylquinolines could be converted to the corresponding products in moderate to good yields (3ba–3ia). The substrates with electron-withdrawing groups presented more efficient than that with the electron-donating groups in this reaction. For example, 2-methyl-6-methoxylquinoline only afforded the product (3ha) with 44% yield while 2-methyl-6-nitroquinoline gave the product with a high yield of 81% (3ga). Subsequently, the substitution position of methoxyl group was investigated. The yields between 4-methoxyl and 6-methoxyl product were almost the same. However, the 8-methoxyl product was obtained with an abnormal yield of 79% (3ha, 3ja and 3ka). This implied that steric hindrance had little influence on the reaction when 2-methyl-8-phenylquinoline was employed (3ia*vs.*3la). The 2-ethylquinoline showed lower reactivity. We could not obtain the desired product under the standard conditions but only the corresponding ketone was obtained. The desired imidazo[1,5-*a*]quinoline can be obtained with a yield of 50% when the reaction temperature was increased to 120 °C (3ma). Agreeing with former reports,^11*b*^ an electron-withdrawing substituent on the carbon of methyl at 2-position favoured the reaction (3na). 1-Methylisoquinoline could also converted to the desired product well under standard conditions (3oa).

**Table 2:** Substrate scope of various quinolines^a^

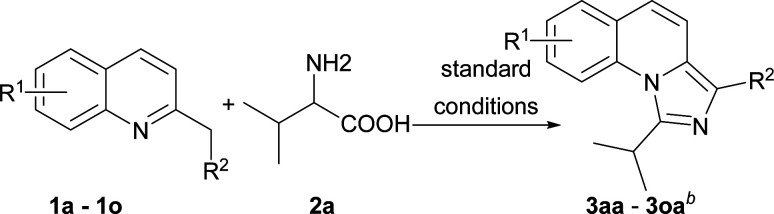
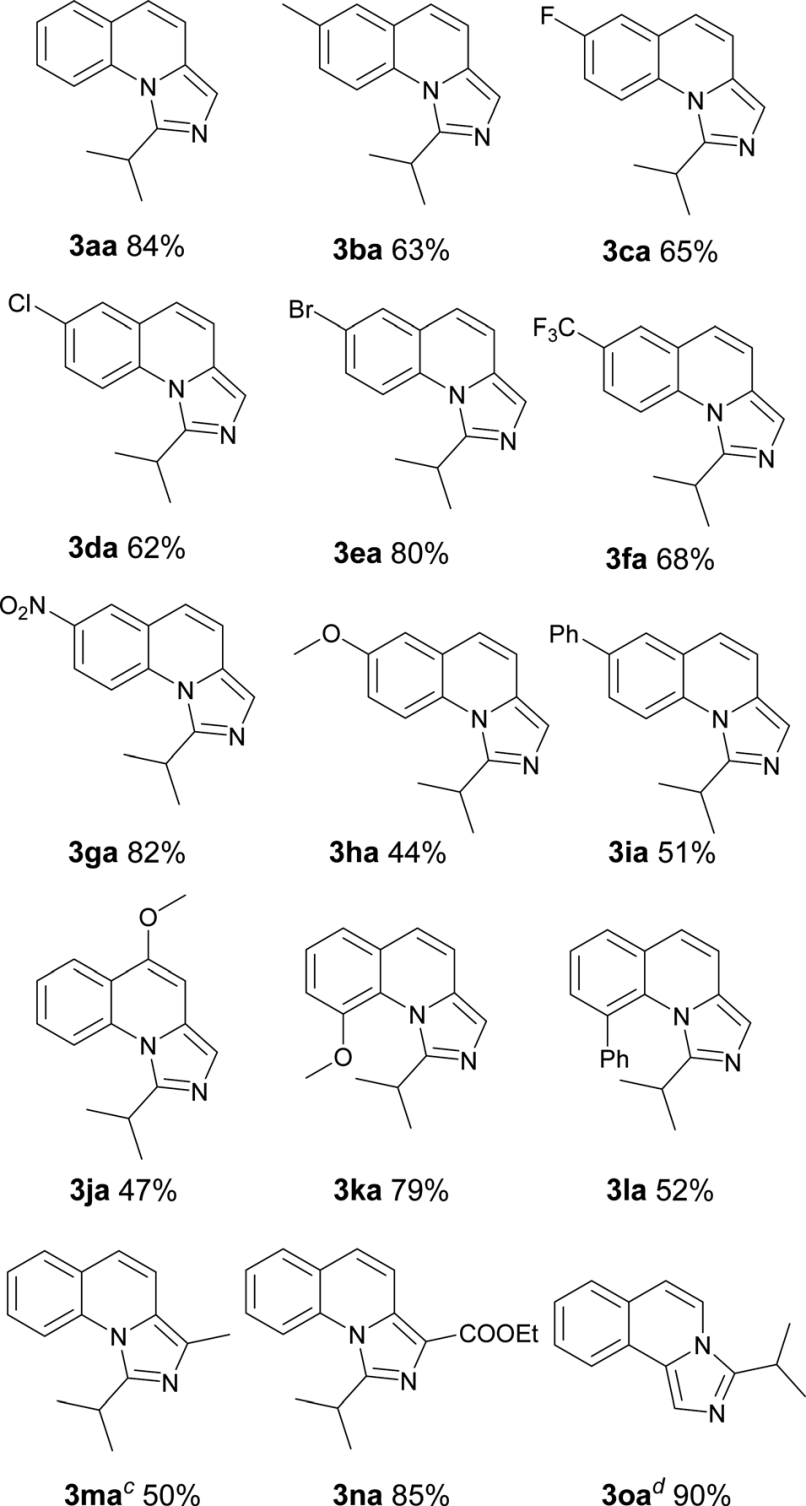

a Reaction conditions: 1a–1o (1.0 equiv., 0.2 mmol), 2a (1.5 equiv., 0.3 mmol), I_2_ (1.0 equiv.), oxidant (3 equiv.) in solvent (0.5 mL).

b Isolated yield.

c This reaction was carried out at 120 °C.

d This product was obtained with the starting material of 1-methylisoquinoline so the structure was different from 3 as shown.

On the other hand, the scope of the amino acids were investigated (Table 3). Alkyl amino acids worked well in this reaction to afford the desired products with moderate to good yields (3ab–3ah), while the aromatic amino acids, such as phenylglycine and phenylalanine, can also be employed as the substrate to afford the corresponding products with lower yields (3aj–3ak). This may be due to the fact that under these conditions, aromatic amino acids are more active and some side reactions can occur. For instance, part of the amino acids might be decarboxylated first, which could not convert to desired products. As for tyrosine, the phenolic hydroxyl group could tolerate the reaction conditions to give the desired product with good yield (3al). To our delight, non-substituted imidazo[1,5-*a*]quinoline (3ai) can be obtained at 120 °C by virtue of this method, which was challenging from other methods.^9*a*,11*a*^

**Table 3:** Substrate scope of various amino acids^a^

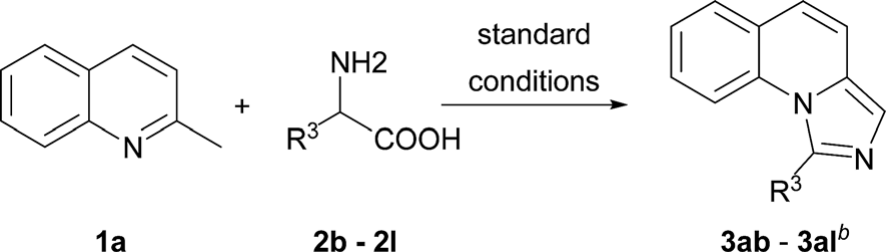
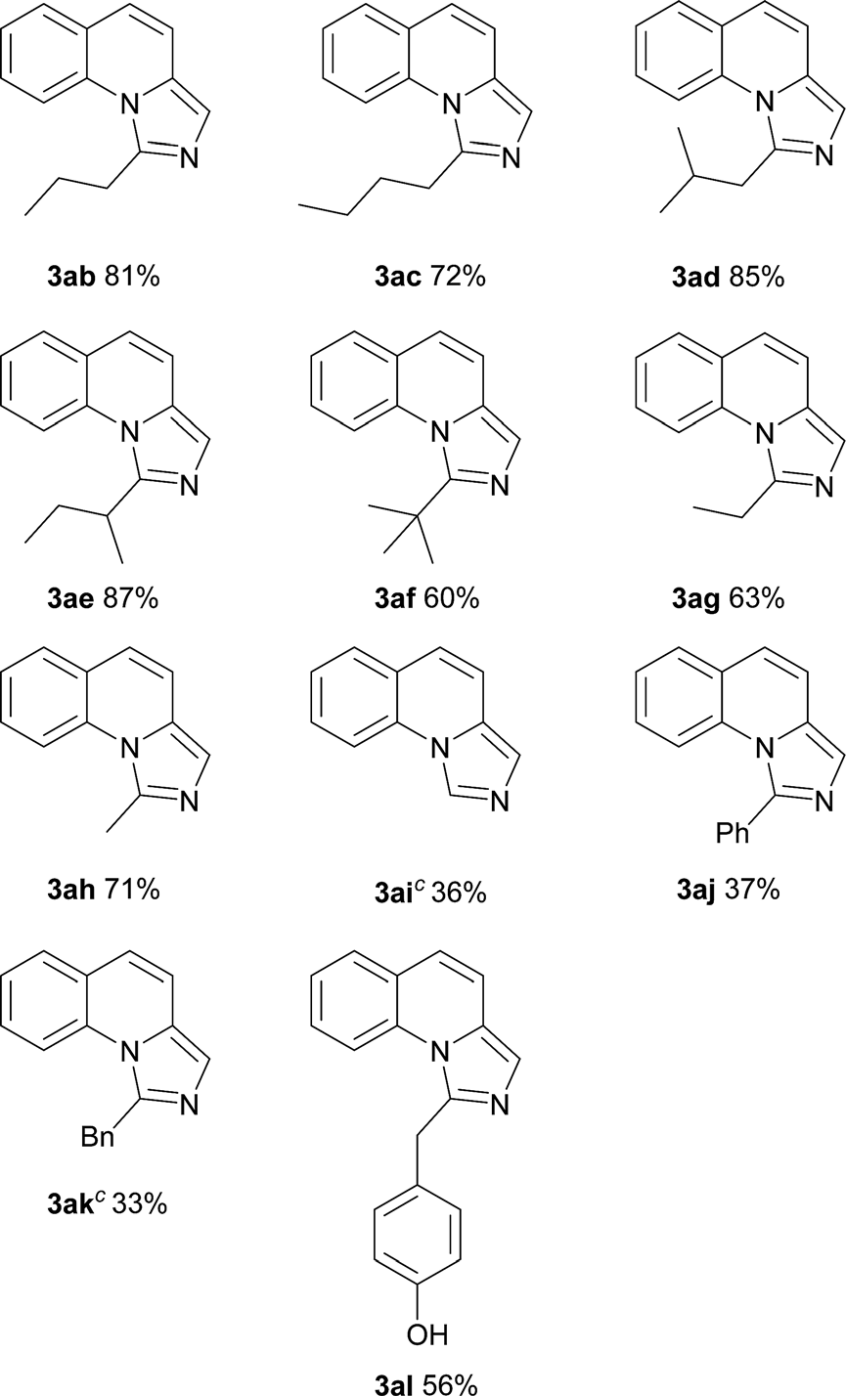

a Reaction conditions: 2b–2o (1.0 equiv., 0.2 mmol), 2a (1.5 equiv., 0.3 mmol), I_2_ (1.0 equiv.), oxidant (3 equiv.) in solvent (0.5 mL).

b Isolated yield.

c This reaction was carried out at 120 °C.

**Scheme 2:**
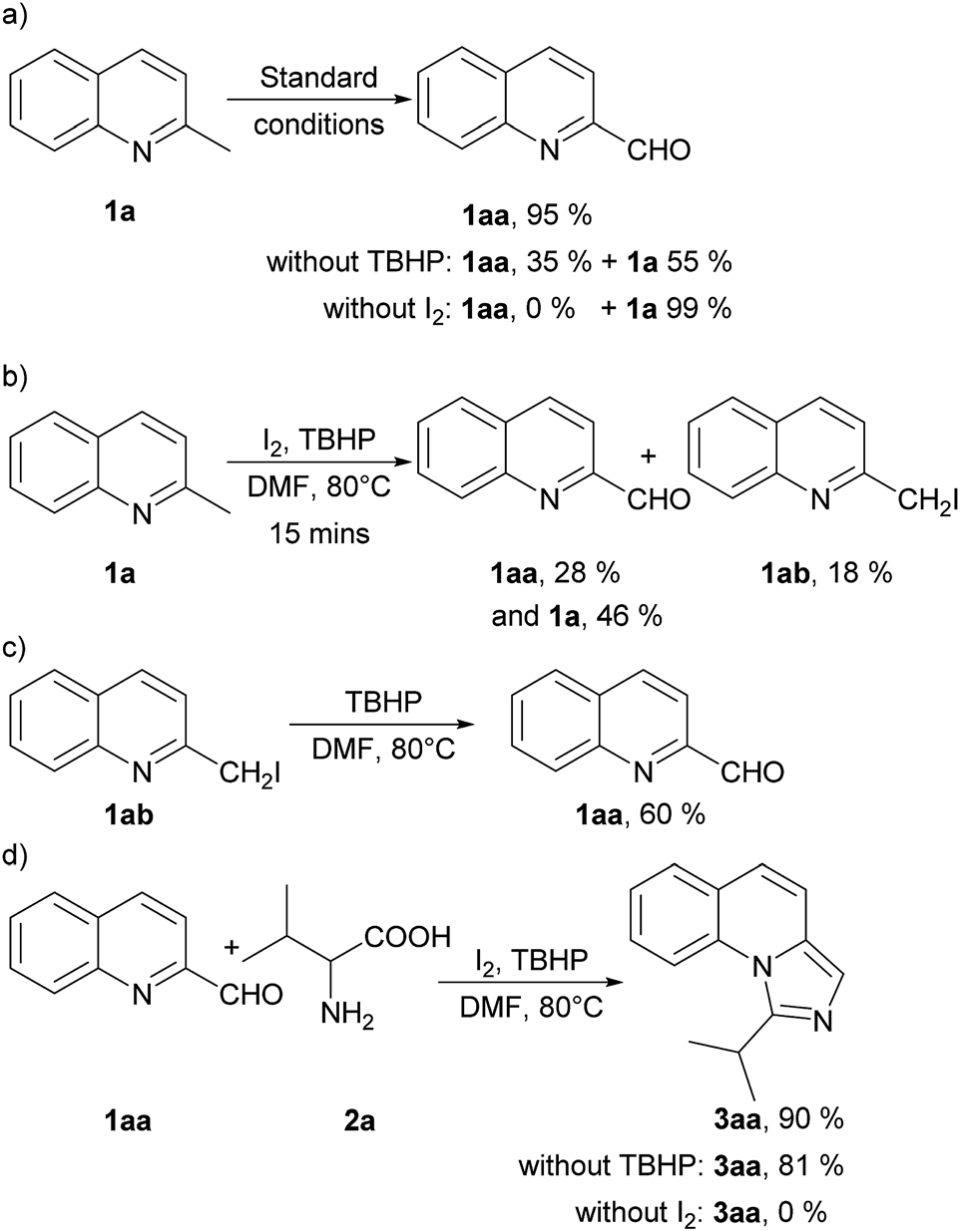
Control experiments.

To get an insight into the mechanism of this process, we conducted several control experiments (Scheme 2). Firstly, only 2-methylquinoline (1a) was employed under the standard conditions and quinoline-2-carbaldehyde (1aa) was obtained with a yield of 95%. The yield decreased when TBHP was absent with 55% of 1a recovered, and no corresponding aldehyde was found without iodine (Scheme 2a). Besides, the aldehyde (1aa) and 2-(iodomethyl) quinoline (1ab) could be detected at 15 minutes of the model reaction (Scheme 2b). Moreover, 2-(iodomethyl) quinoline (1ab) was employed under the standard conditions without I_2_ and 1aa was obtained with a yield of 60% (Scheme 2c). When quinoline-2-carbaldehyde (1aa) and valine (2a) were used as the reaction substrates under standard conditions, 90% of 3aa could be afforded. It should be noted that the yield decreased slightly when TBHP was absent, but no corresponding product 3aa was detected without iodine (Scheme 2d). These experiments indicated that 1aa might be an intermediate of this reaction under the promotion of iodine and TBHP. In the subsequent transformation, TBHP might not be important but I_2_ is needed.

**Scheme 3:**
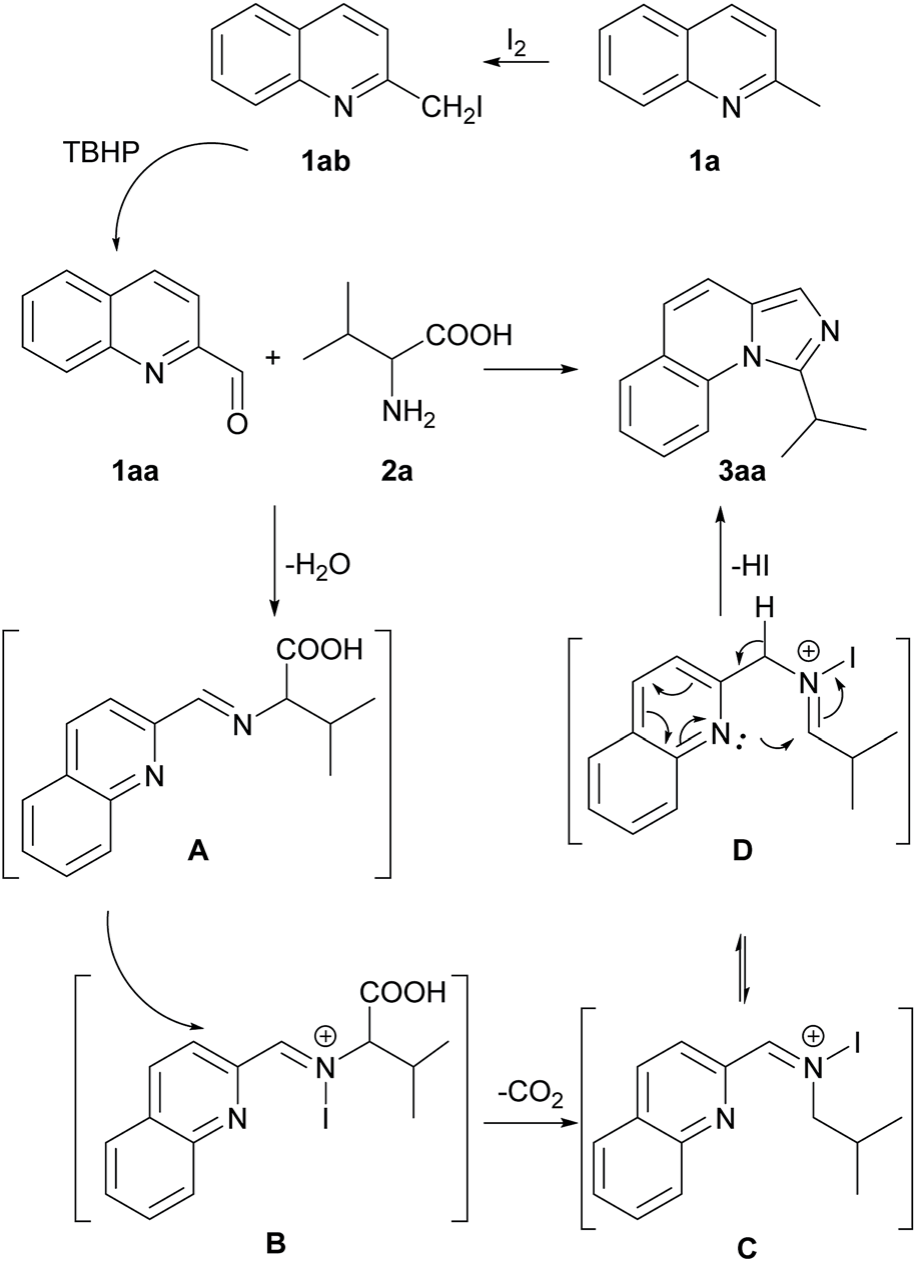
The proposed reaction mechanism.

Based on the experimental results above and the previous reports,^10*c*,11*a*^ a possible reaction pathway was proposed as shown in Scheme 3. Initially, 2-methylquinoline (1a) is quickly substituted with iodine to afford 2-(iodomethyl) quinoline (1ab) and subsequently oxidized to quinoline-2-carbaldehyde (1aa). Thereafter, 1aa produces imine A with the amino acid 2a. The imine A goes through *N*-iodination process, generating intermediate B. Afterward, the intermediate B undergoes a decarboxylative pathway to generate intermediate C at high temperature. Finally, C transforms to D and then cyclization happens easily through an intramolecular nucleophilic attack to give the final product 3aa.

## Conclusions

In summary, we developed a facile decarboxylative cyclization to construct imidazo[1,5-*a*]quinolines with readily available starting material under metal-free condition. Compared to previous reports, the substrate scope of the primary α-amino acid was largely extended. In particular, the synthesis avoids the metal residues in the products, which provides a useful method for the pharmaceutical synthesis.

## Experimental

### Materials and methods

Products were purified by flash chromatography on 200–300 mesh silica gels using petroleum ether/ethyl acetate as eluent. NMR spectra were recorded on 400 MHz (101 MHz for ^13^C NMR) Bruker NMR spectrometer with CDCl_3_ or DMSO-d^6^ as the solvent and tetramethylsilane (TMS) as the internal standard. High resolution mass spectra (ESI) were recorded on a Waters™ Q-TOF Premier. Melting points were determined on a melting point apparatus and are uncorrected.

### General procedure for the synthesis of imidazo[1,5-*a*]quinolines (3)

2-Methyl quinoline (1a, 28 mg, 0.2 mmol), valine (2a, 35 mg, 0.3 mmol) and iodine (51 mg, 0.2 mmol) were added to 0.5 mL of *N*,*N*-dimethylformamide. Then 60 μL of *tert*-butyl hydroperoxide was added and the solution was kept at 80 °C for 3 h. After the reaction was finished, the reaction mixture was washed and extracted by CH_2_Cl_2_. The organic phase was dried by Na_2_SO_4_ and then evaporated under reduced pressure. The resulting residue was purified by silica gel column chromatography (PE/EtOAc) to afford the desired product 3.

### 1-Isopropyl imidazo[1,5-*a*]quinoline (3aa)

The title compound was prepared according to the general working procedure and purified by column chromatography to give the product as a yellow oil. Silica gel TLC Rf = 0.6 (PE : EtOAc = 3 : 1); ^1^H NMR (400 MHz, CDCl_3_) *δ* 8.07–8.05 (d, *J* = 8.5 Hz, 1H), 7.52–7.50 (d, *J* = 7.7 Hz, 1H), 7.41–7.37 (t, *J* = 7.9 Hz, 1H), 7.27–7.24 (m, 2H), 7.15–7.12 (d, *J* = 9.6 Hz, 1H), 6.81–6.79 (d, *J* = 9.3 Hz, 1H), 3.77–3.67 (sept, *J* = 6.6 Hz, 1H), 1.47–1.45 (d, *J* = 6.6 Hz, 6H). ^13^C NMR (101 MHz, CDCl_3_) *δ* 149.3, 133.1, 130.2, 128.7, 127.7, 126.0, 124.8, 120.6, 117.5, 116.9, 30.0, 21.6; HRMS (ESI) *m*/*z* calcd for C_14_H_15_N_2_ [M + H]^+^ 211.1235, found 211.1236.

### 1-Isopropyl-7-methyl-imidazo[1,5-*a*]quinoline (3ba)

The title compound was prepared according to the general working procedure and purified by column chromatography to give the product as a brown oil. Silica gel TLC Rf = 0.45 (PE : EtOAc = 3 : 1); ^1^H NMR (400 MHz, CDCl_3_) *δ* 8.06–8.04 (d, *J* = 8.6 Hz, 1H), 7.40 (s, 1H), 7.35 (s, 1H), 7.31–7.29 (d, *J* = 8.6 Hz, 1H), 7.21 (d, *J* = 8.1 Hz, 1H), 6.86–6.84 (d, *J* = 9.4 Hz, 1H), 3.85–3.75 (sept, *J* = 6.7 Hz, 1H), 2.44 (s, 3H), 1.56–1.54 (d, *J* = 6.7 Hz, 6H); ^13^C NMR (101 MHz, CDCl_3_) *δ* 149.0, 134.4, 131.0, 130.0, 128.7, 128.7, 126.0, 120.5, 117.4, 116.8, 29.8, 21.5, 20.7; HRMS (ESI) *m*/*z* calcd for C_15_H_17_N_2_ [M + H]^+^ 225.1392, found 225.1390.

### 7-Flouro-1-isopropyl-imidazo[1,5-*a*]quinoline (3ca)

The title compound was prepared according to the general working procedure and purified by column chromatography to give the product as a yellow solid. Silica gel TLC Rf = 0.6 (PE : EtOAc = 3 : 1); mp = 74–76 °C; ^1^H NMR (400 MHz, CDCl_3_) *δ* 8.16–8.12 (dd, *J* = 9.1, 4.4 Hz, 1H), 7.40 (s, 1H), 7.31–7.27 (m, 2H), 7.25–7.20 (m, 1H), 6.87–6.84 (d, *J* = 9.3 Hz, 1H), 3.81–3.71 (sept, *J* = 6.7 Hz, 1H), 1.56 (d, *J* = 6.7 Hz, 6H); ^13^C NMR (101 MHz, CDCl_3_) *δ* 160.4, 157.9 (d, ^1^*J*_CF_ = 251.5 Hz), 149.2, 129.9, 129.5, 129.5 (d, ^4^*J*_CF_ = 2.2 Hz), 127.9, 127.8 (d, ^3^*J*_CF_ = 8.3 Hz), 121.2, 119.7, 119.7 (d, ^4^*J*_CF_ = 2.7 Hz), 118.8, 118.6, 118.5 (d, ^3^*J*_CF_ = 8.5 Hz), 115.0, 114.8 (d, ^2^*J*_CF_ = 23.5 Hz), 114.0, 113.7 (d, ^2^*J*_CF_ = 22.3 Hz), 29.9, 21.4; HRMS (ESI) *m*/*z* calcd for C_14_H_14_N_2_F [M + H]^+^ 229.1141, found 229.1138.

### 7-Chloro-1-isopropyl-imidazo[1,5-*a*]quinoline (3da)

The title compound was prepared according to the general working procedure and purified by column chromatography to give the product as a yellow oil. Silica gel TLC Rf = 0.6 (PE : EtOAc = 2 : 1) ^1^H NMR (400 MHz, CDCl_3_) *δ* 8.10–8.08 (d, *J* = 9.0 Hz, 1H), 7.59 (s, 1H), 7.46–7.44 (d, *J* = 9.0 Hz, 1H), 7.39 (s, 1H), 7.30–7.27 (d, *J* = 9.6 Hz, 1H), 6.84–6.82 (d, *J* = 9.4 Hz, 1H), 3.80–3.70 (sept, *J* = 6.7 Hz, 1H), 1.57–1.55 (d, *J* = 6.7 Hz, 6H); ^13^C NMR (101 MHz, CDCl_3_) *δ* 149.5, 131.5, 130.17, 130.0, 127.8, 127.5, 127.5, 121.3, 119.4, 118.8, 118.3, 30.0, 21.4; HRMS (ESI) *m*/*z* calcd for C_14_H_14_N_2_Cl [M + H]^+^ 245.0846, found 245.0844.

### 7-Bromo-1-isopropyl-imidazo[1,5-*a*]quinoline (3ea)

The title compound was prepared according to the general working procedure and purified by column chromatography to give the product as a yellow oil. Silica gel TLC Rf = 0.15 (PE : EtOAc = 10 : 1); ^1^H NMR (400 MHz, CDCl_3_) *δ* 8.03 (m, 1H), 7.75–7.74 (d, *J* = 2.3 Hz, 1H), 7.60–7.57 (m, 1H), 7.39 (s, 1H), 7.29–7.27 (m, 1H), 6.83–6.80 (m, 1H), 3.80–3.69 (sept, *J* = 6.6 Hz, 1H), 1.56–1.54 (d, *J* = 6.6 Hz, 6H); ^13^C NMR (101 MHz, CDCl_3_) *δ* 149.5, 131.9, 130.9, 130.3, 130.0, 127.9, 121.3, 119.3, 118.8, 118.5, 117.8, 30.0, 21.3; HRMS (ESI) *m*/*z* calcd for C_14_H_14_N_2_Br [M + H]^+^ 289.0340, found 289.0340.

### 1-Isopropyl-7-(trifluoromethyl)-imidazo[1,5-*a*]quinoline (3fa)

The title compound was prepared according to the general working procedure and purified by column chromatography to give the product as a yellow needle solid. Silica gel TLC Rf = 0.5 (PE : EtOAc = 3 : 1); mp = 60–62 °C; ^1^H NMR (400 MHz, CDCl_3_) *δ* 8.27–8.24 (d, *J* = 8.9 Hz, 1H), 7.88 (s, 1H), 7.74–7.72 (d, *J* = 8.9 Hz, 1H), 7.41 (s, 1H), 7.34–7.32 (d, *J* = 9.3 Hz, 1H), 6.95–6.92 (d, *J* = 9.3 Hz, 1H), 3.84–3.74 (sept, *J* = 6.6 Hz, 1H), 1.57 (d, *J* = 6.6 Hz, 6H); ^13^C NMR (101 MHz, CDCl_3_) *δ* 150.0, 135.0, 130.2, 127.8, 125.1, 122.4, 119.7 (q, ^1^*J*_CF_ = 272.0 Hz), 127.4, 127.0, 126.7, 126.4 (q, ^2^*J*_CF_ = 33.0 Hz), 126.1, 125.8, 125.7, 125.7, 125.7 (q, *J*_CF_ = 3.9 Hz), 124.2, 124.2, 124.1, 124.1 (q, *J*_CF_ = 3.4 Hz), 121.5, 119.9, 119.1, 117.4, 30.1, 21.4; HRMS (ESI) *m*/*z* calcd for C_15_H_14_N_2_F_3_ [M + H]^+^ 279.1109, found 279.1107.

### 1-Isopropyl-7-nitro-imidazo[1,5-*a*]quinoline (3ga)

The title compound was prepared according to the general working procedure and purified by column chromatography to give the product as an orange needle solid. Silica gel TLC Rf = 0.45 (PE : EtOAc = 3 : 1); mp = 190–192 °C; ^1^H NMR (400 MHz, CDCl_3_) *δ* 8.47–8.24 (m, 3H), 7.44–7.43 (d, *J* = 4.8 Hz, 1H), 7.40–7.36 (dd, *J* = 9.3, 5.5 Hz, 1H), 7.00–6.96 (m, 1H), 3.79–3.74 (sept, *J* = 6.4 Hz, 1H), 1.57 (d, *J* = 6.5, 6H); ^13^C NMR (101 MHz, CDCl_3_) *δ* 150.5, 143.9, 136.7, 130.2, 126.5, 123.8, 122.3, 122.1, 120.0, 119.8, 117.6, 30.2, 21.4; HRMS (ESI) *m*/*z* calcd for C_14_H_14_N_3_O_2_ [M + H]^+^ 256.1086, found 256.1083.

### 1-Isopropyl-7-methoxy-imidazo[1,5-*a*]quinoline (3ha)

The title compound was prepared according to the general working procedure and purified by column chromatography to give the product as a brown oil. Silica gel TLC Rf = 0.30 (PE : EtOAc = 3 : 1); ^1^H NMR (400 MHz, CDCl_3_) *δ* 8.11–8.08 (d, *J* = 8.8 Hz, 1H), 7.37 (s, 1H), 7.26–7.24 (d, *J* = 9.6 Hz, 1H), 7.10–7.08 (m, 2H), 6.88–6.86 (d, *J* = 9.3 Hz, 1H), 3.90 (s, 3H), 3.84–3.74 (sept, *J* = 6.7 Hz, 1H), 1.56 (d, *J* = 6.7 Hz, 6H); ^13^C NMR (101 MHz, CDCl_3_) *δ* 156.3, 148.7, 129.9, 127.4, 127.3, 120.6, 120.4, 118.2, 118.0, 115.1, 111.2, 55.5, 29.8, 21.5; HRMS (ESI) *m*/*z* calcd for C_15_H_17_N_2_O [M + H]^+^ 241.1341, found 241.1342.

### 1-Isopropyl-7-phenyl-imidazo[1,5-*a*]quinoline (3ia)

The title compound was prepared according to the general working procedure and purified by column chromatography to give the product as a yellow oil. Silica gel TLC Rf = 0.15 (PE : EtOAc = 10 : 1); ^1^H NMR (400 MHz, CDCl_3_) *δ* 8.24–8.22 (d, *J* = 8.8 Hz, 1H), 7.82 (s, 1H), 7.75–7.73 (d, *J* = 8.8 Hz, 1H), 7.67–7.65 (d, *J* = 7.5 Hz, 4H), 7.50–7.46 (t, *J* = 7.5 Hz, 2H), 7.40–7.37 (m, 2H), 7.28–7.26 (t, *J* = 6.8 Hz, 1H), 6.98–6.96 (d, *J* = 9.3 Hz, 2H), 3.90–3.80 (sept, *J* = 6.6 Hz, 1H), 1.59 (d, *J* = 6.6 Hz, 6H); ^13^C NMR (101 MHz, CDCl_3_) *δ* 149.4, 139.7, 137.6, 130.2, 128.9, 127.6, 127.0, 126.8, 126.5, 126.4, 120.8, 120.7, 117.9, 117.3, 30.0, 21.5. HRMS (ESI) *m*/*z* calcd for C_20_H_19_N_2_ [M + H]^+^ 287.1548, found 287.1550.

### 1-Isopropyl-5-methoxy-imidazo[1,5-*a*]quinoline (3ja)

The title compound was prepared according to the general working procedure and purified by column chromatography to give the product as a green oil. Silica gel TLC Rf = 0.50 (PE : EtOAc = 3 : 1); ^1^H NMR (400 MHz, CDCl_3_) *δ* 8.15–8.12 (d, *J* = 8.7 Hz, 1H), 8.08–8.06 (d, *J* = 8.1 Hz, 1H), 7.57–7.53 (m, 1H), 7.42–7.38 (m, 1H), 7.17 (s, 1H), 6.50 (s, 1H), 3.94 (m, 3H), 3.80–3.71 (sept, *J* = 6.5 Hz, 1H), 1.54 (d, *J* = 6.6 Hz, 6H); ^13^C NMR (101 MHz, CDCl_3_) *δ* 148.5, 147.9, 133.4, 130.1, 128.5, 124.4, 123.4, 121.3, 117.9, 116.7, 91.9, 55.3, 30.0, 21.5; HRMS (ESI) *m*/*z* calcd for C_15_H_17_N_2_O [M + H]^+^ 241.1341, found 241.1338.

### 1-Isopropyl-9-methoxy-imidazo[1,5-*a*]quinoline (3ka)

The title compound was prepared according to the general working procedure and purified by column chromatography to give the product as a green oil. Silica gel TLC Rf = 0.55 (PE : EtOAc = 3 : 1) ^1^H NMR (400 MHz, CDCl_3_) *δ* 7.41 (s, 1H), 7.33–7.29 (m, 1H), 7.18–7.16 (d, *J* = 8.3 Hz, 2H), 7.00–6.98 (d, *J* = 8.1 Hz, 1H), 6.79–6.77 (d, *J* = 9.2 Hz, 1H), 3.92 (m, 3H), 3.75–3.65 (sept, *J* = 6.7 Hz, 1H), 1.36 (d, *J* = 6.7 Hz, 6H); ^13^C NMR (101 MHz, CDCl_3_) *δ* 154.6, 149.7, 130.5, 129.1, 125.7, 122.7, 121.6, 119.7, 118.0, 109.9, 55.3, 30.2, 22.9; HRMS (ESI) *m*/*z* calcd for C_15_H_17_N_2_O [M + H]^+^ 241.1341, found 241.1345.

### 1-Isopropyl-9-phenyl-imidazo[1,5-*a*]quinoline (3la)

The title compound was prepared according to the general working procedure and purified by column chromatography to give the product as a yellow oil. Silica gel TLC Rf = 0.35 (PE: EtOAc = 10: 1); ^1^H NMR (400 MHz, CDCl_3_) *δ* 7.56–7.53 (m, 1H), 7.45–7.43 (d, *J* = 9.2 Hz, 3H), 7.40–7.36 (t, *J* = 7.4 Hz, 2H), 7.32–7.28 (t, *J* = 7.4 Hz, 1H), 7.26–7.24 (d, *J* = 9.4 Hz, 1H), 7.20–7.18 (d, *J* = 6.9 Hz, 2H), 6.92–6.90 (d, *J* = 9.2 Hz, 1H), 2.83–2.73 (sept, *J* = 6.7 Hz, 1H), 0.69–0.67 (d, *J* = 6.7 Hz, 6H); ^13^C NMR (101 MHz, CDCl_3_) *δ* 154.5, 141.0, 133.0, 131.0, 130.9, 129.5, 129.2, 129.0, 127.60, 127.6, 126.5, 125.6, 122.1, 120.3, 117.7, 29.8, 21.3; HRMS (ESI) *m*/*z* calcd for C_20_H_19_N_2_ [M + H]^+^ 287.1548, found 287.1552.

### 1-Isopropyl-3-methyl-imidazo[1,5-*a*]quinoline (3ma)

The title compound was prepared according to the general working procedure except the temperature was 120 °C and purified by column chromatography to give the product as a yellow solid. Silica gel TLC Rf = 0.25 (PE : EtOAc = 5 : 1); mp = 110–112 °C; ^1^H NMR (400 MHz, CDCl_3_) *δ* 8.13–8.11 (d, *J* = 8.5 Hz, 1H), 7.60–7.58 (d, *J* = 7.6 Hz, 1H), 7.49–7.45 (t, *J* = 7.4 Hz, 1H), 7.36–7.33 (t, *J* = 7.4 Hz, 1H), 7.20–7.17 (d, *J* = 9.4 Hz, 1H), 6.84–6.81 (d, *J* = 9.4 Hz, 1H), 3.86–3.76 (sept, *J* = 6.7 Hz, 1H), 2.49 (s, 3H), 1.57–1.55 (d, *J* = 6.7 Hz, 6H); ^13^C NMR (101 MHz, CDCl_3_) *δ* 148.0, 133.3, 128.6, 128.5, 127.5, 126.2, 126.1, 124.6, 118.8, 117.1, 116.9, 29.7, 21.7, 12.5; HRMS (ESI) *m*/*z* calcd for C_15_H_17_N_2_ [M + H]^+^ 225.1392, found 225.1390.

### Ethyl 1-isopropyl-imidazo[1,5-*a*]quinoline-3-carboxylate (3na)

The title compound was prepared according to the general working procedure and purified by column chromatography to give the product as a pale yellow solid. Silica gel TLC Rf = 0.2 (PE : DCM = 2 : 1); mp = 118–120 °C; ^1^H NMR (400 MHz, CDCl_3_) *δ* 8.30–8.28 (d, *J* = 8.5 Hz, 1H), 8.15–8.13 (d, *J* = 9.3 Hz, 1H), 7.77–7.75 (d, *J* = 7.5 Hz, 1H), 7.65–7.61 (t, *J* = 7.6 Hz, 1H), 7.57–7.41 (t, *J* = 7.4 Hz, 1H), 7.33–7.31 (d, *J* = 9.5 Hz, 1H), 4.51–4.46 (q, *J* = 7.0 Hz, 2H), 3.93–3.83 (sept, *J* = 6.3 Hz, 1H), 1.64–1.62 (d, *J* = 6.4 Hz, 6H), 1.48–1.45 (t, *J* = 7.0 Hz, 3H); ^13^C NMR (101 MHz, CDCl_3_) *δ* 163.8, 150.0, 134.6, 132.8, 129.2, 128.9, 125.8, 125.7, 125.6, 121.9, 118.1, 117.3, 60.6, 30.3, 21.5, 14.6; HRMS (ESI) *m*/*z* calcd for C_17_H_18_N_2_O_2_Na [M + Na]^+^ 305.1266, found 305.1265.

### 3-Isopropyl-imidazo[5,1-*a*]isoquinoline (3oa)

The title compound was prepared according to the general working procedure and purified by column chromatography to give the product as a pale yellow solid, mp = 73–75 °C, silica gel TLC Rf = 0.3 (PE : EtOAc = 3 : 1); ^1^H NMR (400 MHz, CDCl_3_) *δ* 8.00–7.98 (d, *J* = 7.9 Hz, 1H), 7.77 (s, 1H), 7.65–7.63 (d, *J* = 7.5 Hz, 1H), 7.57–7.55 (d, *J* = 7.7 Hz, 1H), 7.52–7.48 (t, *J* = 7.5 Hz, 1H), 7.42–7.38 (t, *J* = 7.4 Hz, 1H), 6.82–6.80 (d, *J* = 7.4 Hz, 1H), 3.41–3.31 (sept, *J* = 6.8 Hz, 1H), 1.48 (d, *J* = 6.8 Hz, 6H); ^13^C NMR (101 MHz, CDCl_3_) *δ* 146.0, 128.4, 128.1, 127.0, 127.7, 126.6, 125.1, 122.3, 119.5, 118.2, 113.5, 26.1, 20.8; HRMS (ESI) *m*/*z* calcd for C_14_H_15_N_2_ [M + H]^+^ 211.1235, found 211.1235.

### 1-Propyl-imidazo[1,5-*a*]quinoline (3ab)

The title compound was prepared according to the general working procedure and purified by column chromatography to give the product as a yellow oil. Silica gel TLC Rf = 0.40 (PE : EtOAc = 2 : 1); ^1^H NMR (400 MHz, CDCl_3_) *δ* 8.15–8.13 (d, *J* = 8.1 Hz, 1H), 7.65–7.63 (d, *J* = 7.5 Hz, 1H), 7.54–7.50 (m, 1H), 7.41–7.36 (m, 2H), 7.27–7.25 (m, 1H), 6.94–6.92 (d, *J* = 9.5 Hz, 1H), 3.38–3.34 (t, *J* = 7.4 Hz, 2H), 2.07–1.99 (m, 2H), 1.17–1.13 (t, *J* = 7.3 Hz, 3H); ^13^C NMR (101 MHz, CDCl_3_) *δ* 144.1, 133.2, 130.3, 128.7, 127.7, 125.9, 124.8, 120.6, 117.4, 116.6, 34.3, 20.6, 14.0; HRMS (ESI) *m*/*z* calcd for C_14_H_15_N_2_ [M + H]^+^ 211.1235, found 211.1230.

### 1-Butyl-imidazo[1,5-*a*]quinoline (3ac)

The title compound was prepared according to the general working procedure and purified by column chromatography to give the product as an orange oil. Silica gel TLC Rf = 0.65 (PE : EtOAc = 6 : 1); ^1^H NMR (400 MHz, CDCl_3_) *δ* 8.14–8.12 (d, *J* = 8.5 Hz, 1H), 7.63–7.61 (d, *J* = 7.7 Hz, 1H), 7.53–7.49 (t, *J* = 7.8 Hz, 1H), 7.40–7.35 (m, 2H), 7.25–7.23 (m, 1H), 6.92–6.90 (d, *J* = 9.4 Hz, 1H), 3.38–3.35 (t, *J* = 7.7 Hz, 2H), 2.02–1.95 (m, 2H), 1.61–1.54 (m, 2H), 1.05–1.00 (t, *J* = 7.3 Hz, 3H); ^13^C NMR (101 MHz, CDCl_3_) *δ* 144.2, 133.2, 130.3, 128.7, 127.7, 125.9, 124.8, 120.6, 120.6, 117.5, 116.6, 32.1, 29.3, 22.6, 13.9; HRMS (ESI) *m*/*z* calcd for C_15_H_17_N_2_ [M + H]^+^ 225.1392, found 225.1391.

### 1-Isobutyl-imidazo[1,5-*a*]quinoline (3ad)

The title compound was prepared according to the general working procedure and purified by column chromatography to give the product as a yellow oil. Silica gel TLC Rf = 0.25 (PE : EtOAc = 5 : 1); ^1^H NMR (400 MHz, CDCl_3_) *δ* 8.13–8.10 (d, *J* = 8.5 Hz, 1H), 7.65–7.63 (d, *J* = 7.7 Hz, 1H), 7.54–7.50 (t, *J* = 7.8 Hz, 1H), 7.41–7.37 (m, 2H), 7.27–7.25 (m, 1H), 6.95–6.92 (d, *J* = 9.4 Hz, 1H), 3.28–3.26 (d, *J* = 7.0 Hz, 2H), 2.47–2.35 (m, 1H), 1.10–1.09 (d, *J* = 6.6 Hz, 6H); ^13^C NMR (101 MHz, CDCl_3_) *δ* 143.5, 133.1, 130.3, 128.7, 127.7, 125.9, 124.9, 120.6, 120.6, 117.5, 116.6, 41.1, 26.6, 22.6; HRMS (ESI) *m*/*z* calcd for C_15_H_17_N_2_ [M + H]^+^ 225.1392, found 225.1393.

### 1-(*sec*-Butyl)-imidazo[1,5-*a*]quinoline (3ae)

The title compound was prepared according to the general working procedure and purified by column chromatography to give the product as a brown oil. Silica gel TLC Rf = 0.70 (PE : EtOAc = 3 : 1); ^1^H NMR (400 MHz, CDCl_3_) *δ* 8.15–8.13 (d, *J* = 8.6 Hz, 1H), 7.63–7.61 (d, *J* = 7.7 Hz, 1H), 7.52–7.49 (t, *J* = 7.9 Hz, 1H), 7.38–7.35 (m, 2H), 7.27–7.23 (m, 1H), 6.92–6.89 (d, *J* = 9.3 Hz, 1H), 3.64–3.56 (m, 1H), 2.24–2.14 (m, 1H), 1.86–1.75 (m, 1H), 1.55–1.53 (d, *J* = 6.7 Hz, 3H), 1.05–1.01 (t, *J* = 7.4 Hz, 3H); ^13^C NMR (101 MHz, CDCl_3_) *δ* 148.7, 133.2, 130.1, 128.7, 127.6, 126.1, 124.7, 120.7, 120.5, 117.5, 116.9, 36.7, 28.5, 18.9, 11.9; HRMS (ESI) *m*/*z* calcd for C_15_H_17_N_2_ [M + H]^+^ 225.1392, found 225.1396.

### 1-(*tert*-Butyl)-imidazo[1,5-*a*]quinoline (3af)

The title compound was prepared according to the general working procedure and purified by column chromatography to give the product as a yellow oil. Silica gel TLC Rf = 0.55 (PE : EtOAc = 3 : 1); ^1^H NMR (400 MHz, CDCl_3_) *δ* 8.42–8.40 (d, *J* = 8.7 Hz, 1H), 7.63–7.61 (d, *J* = 7.7 Hz, 1H), 7.54–7.50 (t, *J* = 7.9 Hz, 1H), 7.40–7.37 (m, 2H), 7.28–7.26 (d, *J* = 9.1 Hz, 1H), 6.95–6.92 (d, *J* = 9.3 Hz, 1H), 1.75 (s, 9H); ^13^C NMR (101 MHz, CDCl_3_) *δ* 151.5, 133.2, 131.8, 128.7, 126.7, 126.5, 124.8, 120.8, 120.4, 120.3, 117.9, 34.8, 30.5; HRMS (ESI) *m*/*z* calcd for C_15_H_17_N_2_ [M + H]^+^ 225.1392, found 225.1391.

### 1-Ethyl-imidazo[1,5-*a*]quinoline (3ag)

The title compound was prepared according to the general working procedure and purified by column chromatography to give the product as a brown oil. Silica gel TLC Rf = 0.35 (PE : EtOAc = 10 : 1); ^1^H NMR (400 MHz, CDCl_3_) *δ* 8.18–8.16 (d, *J* = 8.5 Hz, 1H), 7.64–7.62 (d, *J* = 7.9 Hz, 1H), 7.53–7.50 (t, *J* = 7.8 Hz, 1H), 7.41–7.37 (m, 2H), 7.27–7.25 (m, 1H), 6.95–6.92 (d, *J* = 9.1 Hz, 1H), 3.44–3.39 (q, *J* = 6.9 Hz, 2H), 1.60–1.56 (t, *J* = 7.3 Hz, 3H); ^13^C NMR (101 MHz, CDCl_3_) *δ* 145.1, 133.2, 130.4, 128.7, 127.8, 125.9, 124.9, 120.7, 120.5, 117.4, 116.6, 25.8, 11.8; HRMS (ESI) *m*/*z* calcd for C_13_H_13_N_2_ [M + H]^+^ 197.1079, found 197.1079.

### 1-Methyl-imidazo[1,5-*a*]quinoline (3ah)

The title compound was prepared according to the general working procedure and purified by column chromatography to give the product as a yellow oil. Silica gel TLC Rf = 0.30 (PE : EtOAc = 6 : 1); ^1^H NMR (400 MHz, CDCl_3_) *δ* 8.23–8.21 (d, *J* = 8.5 Hz, 1H), 7.65–7.63 (dd, *J* = 7.7, 1.4 Hz, 1H), 7.54–7.49 (m, 1H), 7.41–7.37 (m, 1H), 7.33 (s, 1H), 7.26–7.24 (m, 1H), 6.95–6.92 (d, *J* = 9.4 Hz, 1H), 3.09 (s, 3H); ^13^C NMR (101 MHz, CDCl_3_) *δ* 140.0, 133.3, 130.3, 128.6, 127.7, 125.7, 124.9, 120.6, 120.5, 117.3, 116.2, 19.6; HRMS (ESI) *m*/*z* calcd for C_12_H_11_N_2_ [M + H]^+^ 183.0922, found 183.0923.

### Imidazo[1,5-*a*]quinoline (3ai)

The title compound was prepared according to the general working procedure except the temperature was 120 °C and purified by column chromatography to give the product as a brown oil. Silica gel TLC Rf = 0.15 (PE : EtOAc = 3 : 1); ^1^H NMR (400 MHz, CDCl_3_) *δ* 8.65 (s, 1H), 7.98–7.96 (d, *J* = 8.3 Hz, 1H), 7.68–7.66 (d, *J* = 7.8 Hz, 1H), 7.58–7.54 (m, 1H), 7.48–7.41 (m, 2H), 7.34–7.32 (d, *J* = 9.5 Hz, 1H), 7.05–7.03 (d, *J* = 9.5 Hz, 1H); ^13^C NMR (101 MHz, CDCl_3_) *δ* 130.9, 128.8, 128.6, 127.8, 125.6, 124.2, 122.4, 121.3, 116.8, 114.6; HRMS (ESI) *m*/*z* calcd for C_11_H_9_N_2_ [M + H]^+^ 169.0766, found 169.0765.

### 1-Phenyl-imidazo[1,5-*a*]quinoline (3aj)

The title compound was prepared according to the general working procedure and purified by column chromatography to give the product as a pale yellow solid. Silica gel TLC Rf = 0.30 (PE : EtOAc = 6 : 1); mp = 113–115 °C; ^1^H NMR (400 MHz, CDCl_3_) *δ* 7.66–7.60 (m, 3H), 7.54–7.50 (m, 5H), 7.34–7.26 (m, 2H), 7.19–7.14 (m, 1H), 7.03–7.00 (d, *J* = 9.4 Hz, 1H); ^13^C NMR (101 MHz, CDCl_3_) *δ* 142.3, 133.7, 132.3, 130.5, 129.5, 129.2, 128.7, 128.6, 127.3, 125.5, 125.1, 122.3, 121.4, 117.4, 117.1; HRMS (ESI) *m*/*z* calcd for C_17_H_13_N_2_ [M + H]^+^ 245.1079, found 245.1082.

### 1-Benzyl-imidazo[1,5-*a*]quinoline (3ak)

The title compound was prepared according to the general working procedure except the temperature was 120 °C and purified by column chromatography to give the product as a pale yellow solid. Silica gel TLC Rf = 0.40 (PE : EtOAc = 3 : 1); mp = 95–97 °C; ^1^H NMR (400 MHz, CDCl_3_) *δ* 8.00–7.98 (d, *J* = 7.7 Hz, 1H), 7.61–7.59 (m, 1H), 7.47 (s, 1H), 7.35–7.25 (m, 5H), 7.23–7.15 (m, 3H), 6.98–6.96 (d, *J* = 9.4 Hz, 1H), 4.83 (s, 2H); ^13^C NMR (101 MHz, CDCl_3_) *δ* 141.4, 136.9, 132.6, 130.7, 128.9, 128.5, 128.2, 127.8, 126.7, 125.7, 125.0, 121.2, 121.0, 117.3, 116.9, 37.8; HRMS (ESI) *m*/*z* calcd for C_18_H_15_N_2_ [M + H]^+^ 259.1235, found 259.1230.

### 4-(Imidazo[1,5-*a*]quinolin-1-ylmethyl)-phenol (3al)

The title compound was prepared according to the general working procedure and purified by column chromatography to give the product as a brown solid, mp = 224–225 °C. Silica gel TLC Rf = 0.55 (DCM : MeOH = 10 : 1); ^1^H NMR (400 MHz, DMSO) *δ* 9.26 (s, 1H), 8.09–8.07 (d, *J* = 8.2 Hz, 1H), 7.75–7.74 (d, *J* = 7.4 Hz, 1H), 7.47–7.38 (m, 4H), 7.12–7.10 (d, *J* = 9.1 Hz, 1H), 6.91–6.89 (d, *J* = 8.1 Hz, 2H), 6.66–6.64 (d, *J* = 8.0 Hz, 2H), 4.68 (s, 2H); ^13^C NMR (101 MHz, DMSO) *δ* 155.9, 141.9, 131.8, 130.2, 129.0, 128.5, 128.0, 127.1, 125.1, 125.08, 121.0, 120.6, 117.4, 117.0, 115.5, 36.1; HRMS (ESI) *m*/*z* calcd for C_18_H_15_N_2_O [M + H]^+^ 275.1184, found 275.1185.

## Conflicts of interest

There are no conflicts to declare.

## Supplementary Material

RA-008-C8RA03786H-s001
