# Supplementary material for: The synthesis of imidazo[1,5-a]quinolines via a decarboxylative cyclization under metal-free conditions
Source: RSC Adv. 2018 Jun 25;8(41):23058–65. doi: 10.1039/c8ra03786h (PMC9081590; doi:10.1039/c8ra03786h)
Supplement: RA-008-C8RA03786H-s001 [file RA-008-C8RA03786H-s001.pdf]

Electronic Supplementary Material (ESI) for RSC Advances.
This journal is © The Royal Society of Chemistry 2018

Electronic Supporting Information

The synthesis of imidazo[1,5-*a*]quinolines via a decarboxylative cyclization under metal-free condition

Zicong Yan,[a] Changfeng Wan[b], Yu Yang[a], Zhenggen Zha*[a] and Zhiyong Wang*[a]

[a] Hefei National Laboratory for Physical Sciences at Microscale, CAS Key Laboratory of Soft Matter Chemistry & Center for Excellence in Molecular Synthesis of Chinese Academy of Sciences, Collaborative Innovation Center of Suzhou Nano Science and Technology & School of Chemistry and Materials Science, University of Science and Technology of China, 230026, Hefei, Anhui. P. R. China. E-mail: zwang3@ustc.edu.cn; Fax: (+86) 551-6360-3185.
[b]College of Chemistry and Chemical Engineering, Jiangxi Normal University, 330022, Nanchang, P. R. China. E-mail: wanfeng@jxnu.edu.cn.

Contents：

(A) Typical experimental procedure for the synthesis of substrates S2
(B) Copies of NMR spectra for product **3** S3

(A) Typical experimental procedure for the synthesis of substrates

(1) A typical experimental procedure for the synthesis of 2-methyl quinolines:

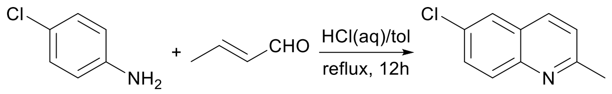

1d

4-Chrolo-aniline (2.54g, 20 mmol), 23.2 mL of 37% HCl (aq) and 10 mL of water were added to a 250 mL round-bottom flask. The mixture was stirred at 100 °C for at least 30 minutes. When the solid were all dissolved, crotonaldehyde (1.75g, 25mmol) in 13 mL of toluene was added and the mixture were kept reflux for 12 h. Then the mixture was cooled to room temperature and poured into 100 mL of Na_2CO_3 (aq). The product was extracted by CH_2Cl_2 and purified by silica gel column chromatography (PE/EtOAc = 15:1) to afford the desired product **1d** (2.52g, 71%).

(2) Experimental procedure for the synthesis of ethyl quinoline-2-acetate (**1n**):

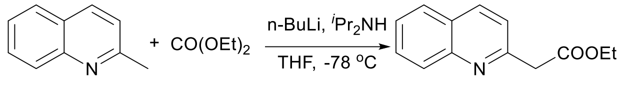

1n

A mixture of 7.08g diisopropylamine (9.8 mL, 70 mmol) and 10 mL of dried THF was added to a three-necked flask by syringe under N_2. After cooling down to -78 °C, 28.4 mL of *n*-Butyl lithium (2.4M in hexane, 68 mmol) was slowly added. The mixture was stirred for 30 minutes. Then 3.5g of 2-methyl quinoline (3.3mL, 24 mmol) was slowly added. The colour turned to orange rapidly then dark-brown. After 30 minutes, 10.6g of diethyl carbonate (11mL, 90 mmol) was added and the mixture was stirred for 2 hours. The reaction was quenched by 10 mL of water. The desired product **1n** (3.3g, 64%) was extracted by EtOAc and purified by silica gel column chromatography (PE/EtOAc = 20:1).

(3) 2-Ethyl quinoline was prepared following the reported methodology[S1].

Reference:

S1 B. Qian, S. Guo, J. Shao, Q. Zhu, L. Yang, C. Xia and H. Huang, *J. Am. Chem. Soc.,* 2010, **132**, 3650 - 3651.

(B) Copies of NMR spectra for product 3

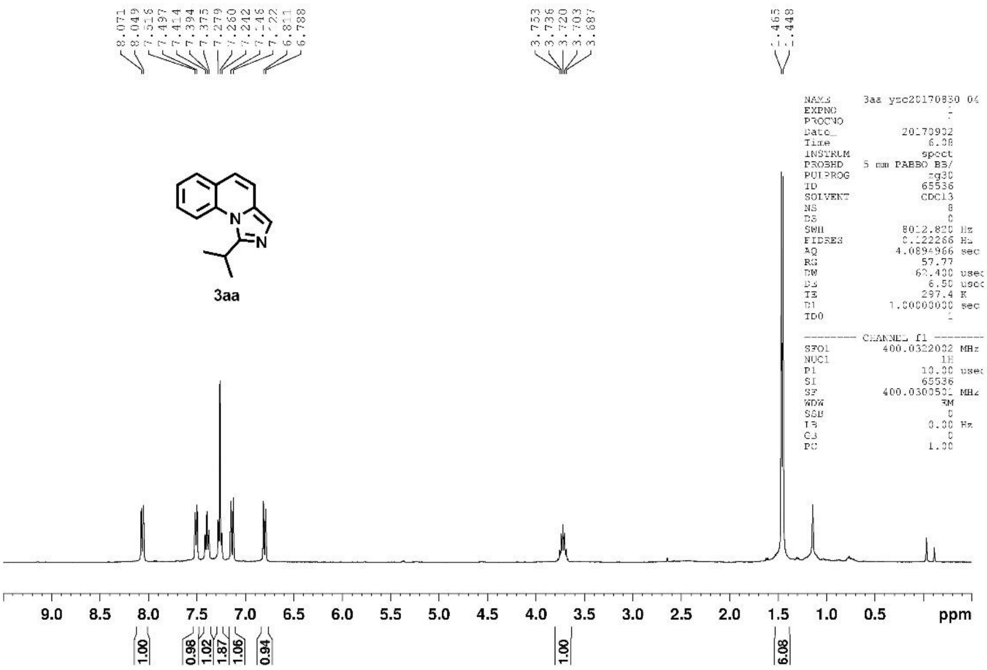

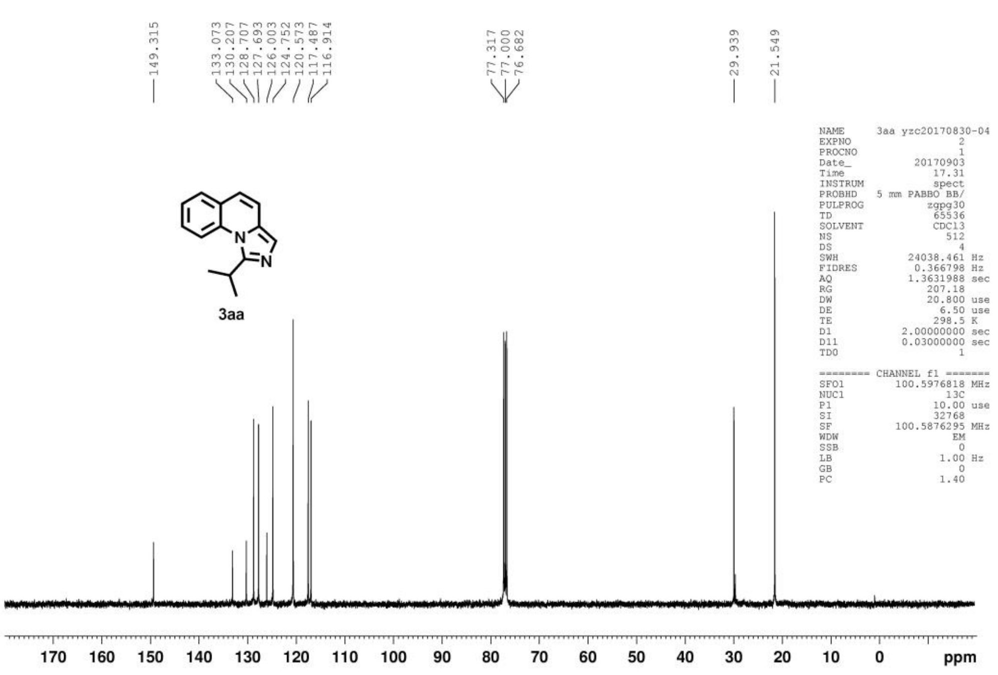

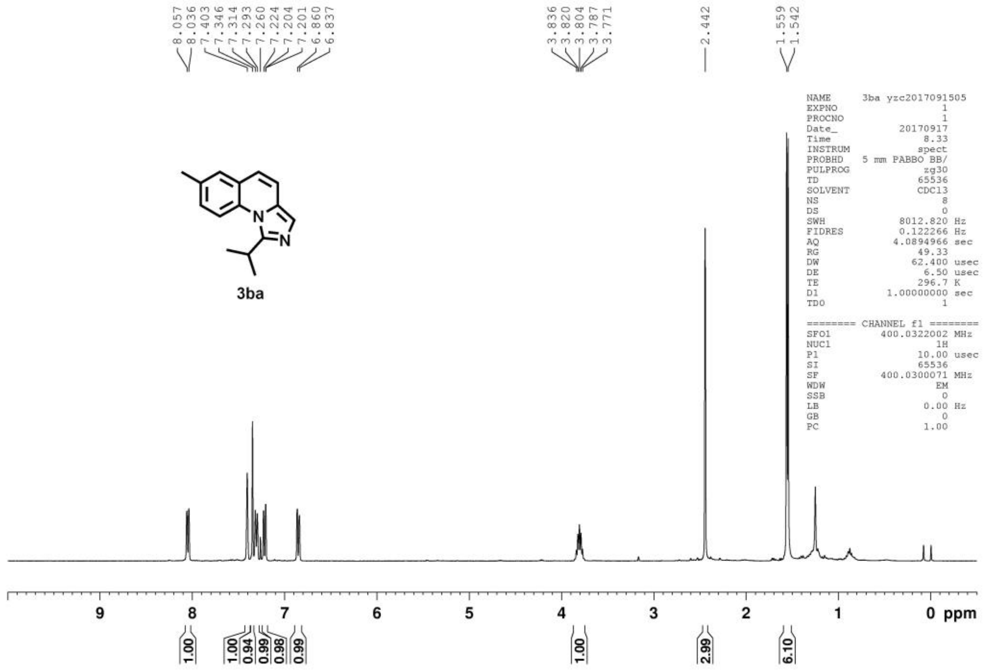
8.057
8.036
7.403
7.346
7.314
7.293
7.260
7.224
7.204
7.201
6.860
6.837
3.836
3.820
3.804
3.787
3.771
2.442
1.559
1.542
3ba
9 8 7 6 5 4 3 2 1 0 ppm
1.00
1.00
0.94
0.99
0.98
0.99
1.00
2.99
6.10

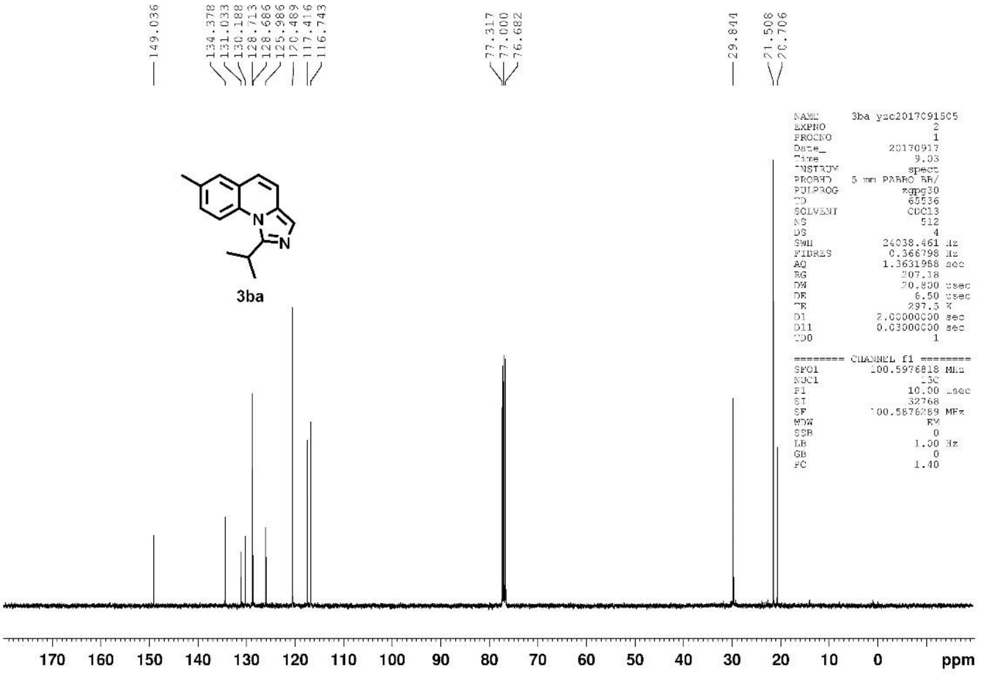
149.036
134.378
131.033
130.188
128.713
128.686
125.986
120.489
117.416
116.743
77.317
77.000
76.682
29.844
21.508
20.706
3ba
170 160 150 140 130 120 110 100 90 80 70 60 50 40 30 20 10 0 ppm

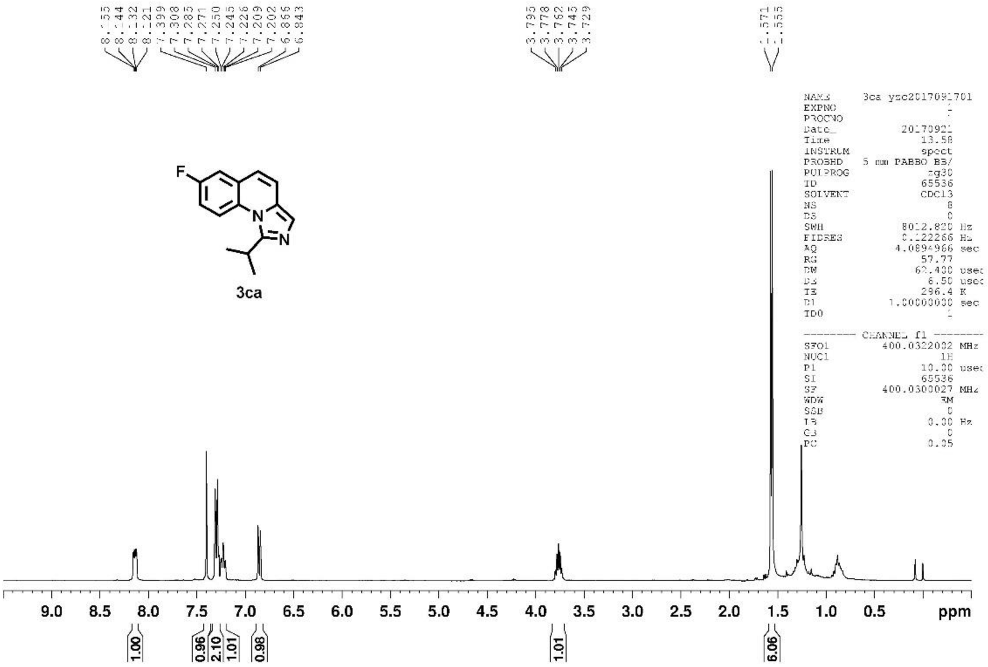
8.155
8.144
8.132
8.121
7.399
7.308
7.285
7.271
7.250
7.245
7.226
7.209
7.202
6.866
6.843
3.795
3.778
3.762
3.745
3.729
1.571
1.555
F
N
N
3ca
NAME 3ca yzc2017091701
SOLVENT CDCl3
SFO1 400.0322032 MHz
NUC1 1H
9.0 8.5 8.0 7.5 7.0 6.5 6.0 5.5 5.0 4.5 4.0 3.5 3.0 2.5 2.0 1.5 1.0 0.5 ppm
1.00
0.96
2.10
1.01
0.98
1.01
6.06

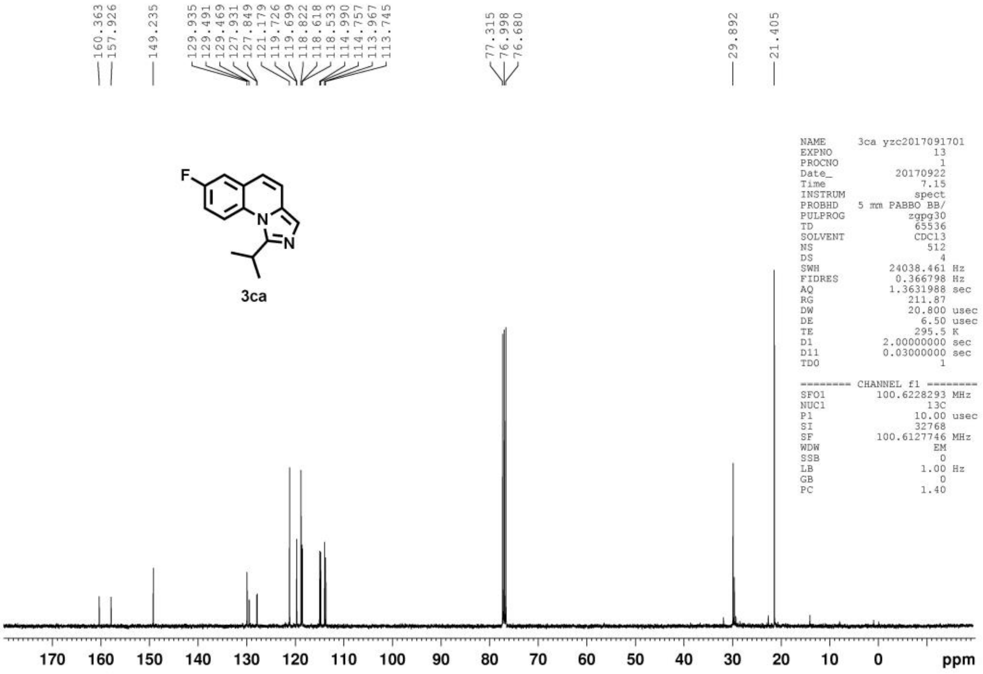
160.363
157.926
149.235
129.935
129.491
129.469
127.931
127.849
121.179
119.726
119.699
118.822
118.618
118.533
114.990
114.757
113.967
113.745
77.315
76.998
76.680
29.892
21.405
F
N
N
3ca
NAME 3ca yzc2017091701
EXPNO 13
PROCNO 1
Date_ 20170922
Time 7.15
INSTRUM spect
PROBHD 5 mm PABBO BB/
PULPROG zgpg30
TD 65536
SOLVENT CDCl3
NS 512
DS 4
SWH 24038.461 Hz
FIDRES 0.366798 Hz
AQ 1.3631988 sec
RG 211.87
DW 20.800 usec
DE 6.50 usec
TE 295.5 K
D1 2.00000000 sec
D11 0.03000000 sec
TD0 1
======== CHANNEL f1 ========
SFO1 100.6228293 MHz
NUC1 13C
P1 10.00 usec
SI 32768
SF 100.6127746 MHz
WDW EM
SSB 0
LB 1.00 Hz
GB 0
PC 1.40
170 160 150 140 130 120 110 100 90 80 70 60 50 40 30 20 10 0 ppm

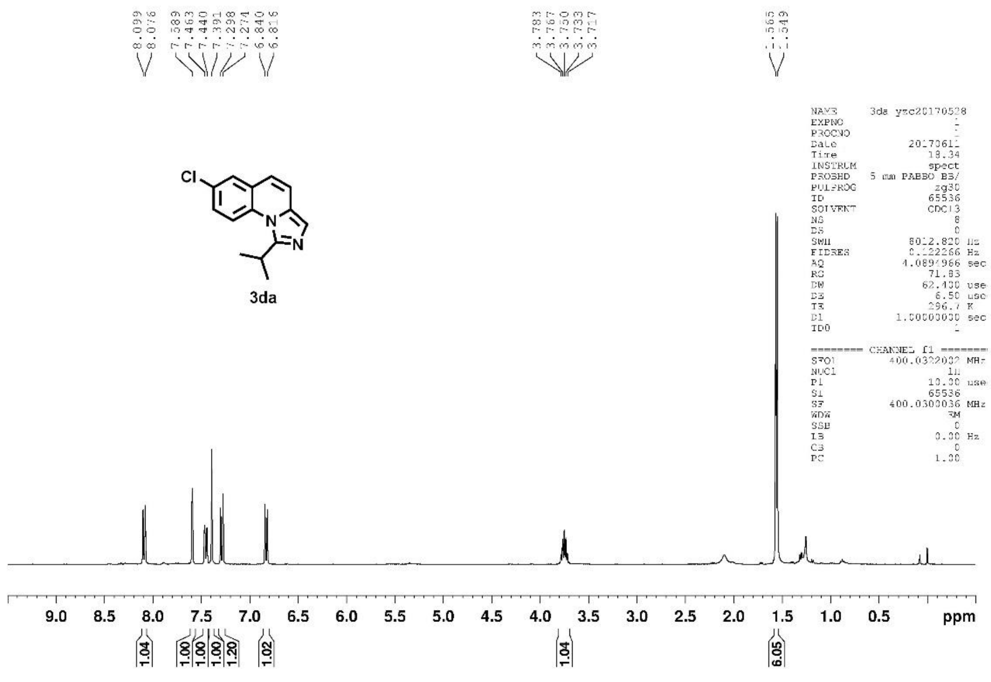
8.099
8.076
7.589
7.463
7.440
7.391
7.298
7.274
6.840
6.816
3.783
3.767
3.750
3.733
3.717
1.565
1.549
Cl
N
N
3da
9.0 8.5 8.0 7.5 7.0 6.5 6.0 5.5 5.0 4.5 4.0 3.5 3.0 2.5 2.0 1.5 1.0 0.5 ppm
1.04
1.00
1.00
1.00
1.20
1.02
1.04
6.05

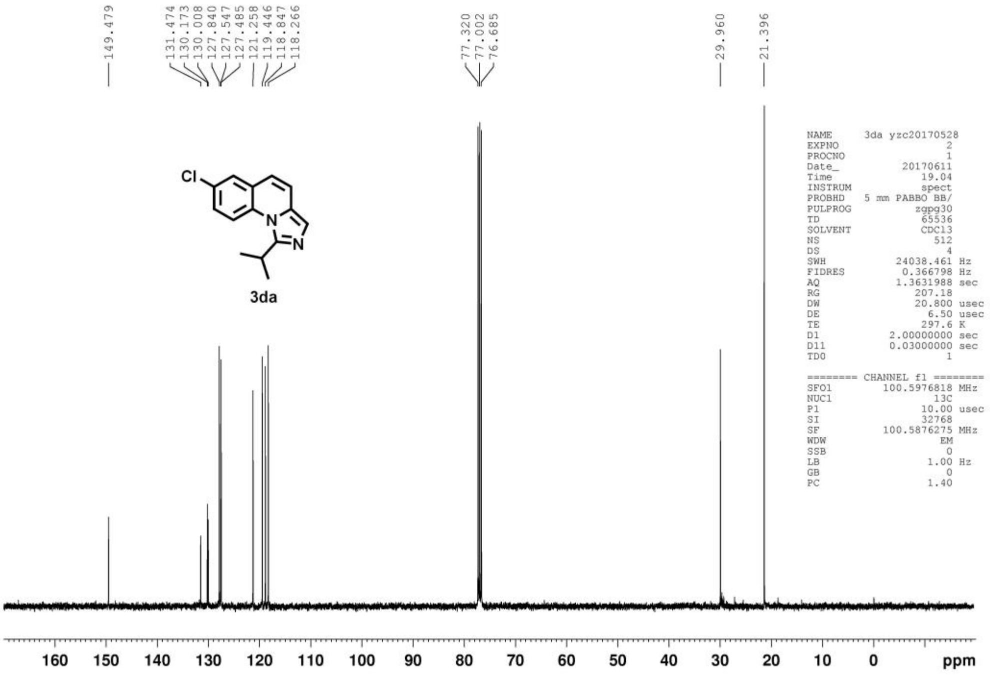
149.479
131.474
130.173
130.008
127.840
127.547
127.485
121.258
119.446
118.847
118.266
77.320
77.002
76.685
29.960
21.396
Cl
N
N
3da
NAME 3da yzc20170528
EXPNO 2
PROCNO 1
Date_ 20170611
Time 19.04
INSTRUM spect
PROBHD 5 mm PABBO BB/
PULPROG zgpg30
TD 65536
SOLVENT CDCl3
NS 512
DS 4
SWH 24038.461 Hz
FIDRES 0.366798 Hz
AQ 1.3631988 sec
RG 207.18
DW 20.800 usec
DE 6.50 usec
TE 297.6 K
D1 2.00000000 sec
D11 0.03000000 sec
TD0 1
======== CHANNEL f1 ========
SFO1 100.5976818 MHz
NUC1 13C
P1 10.00 usec
SI 32768
SF 100.5876275 MHz
WDW EM
SSB 0
LB 1.00 Hz
GB 0
PC 1.40
160 150 140 130 120 110 100 90 80 70 60 50 40 30 20 10 0 ppm

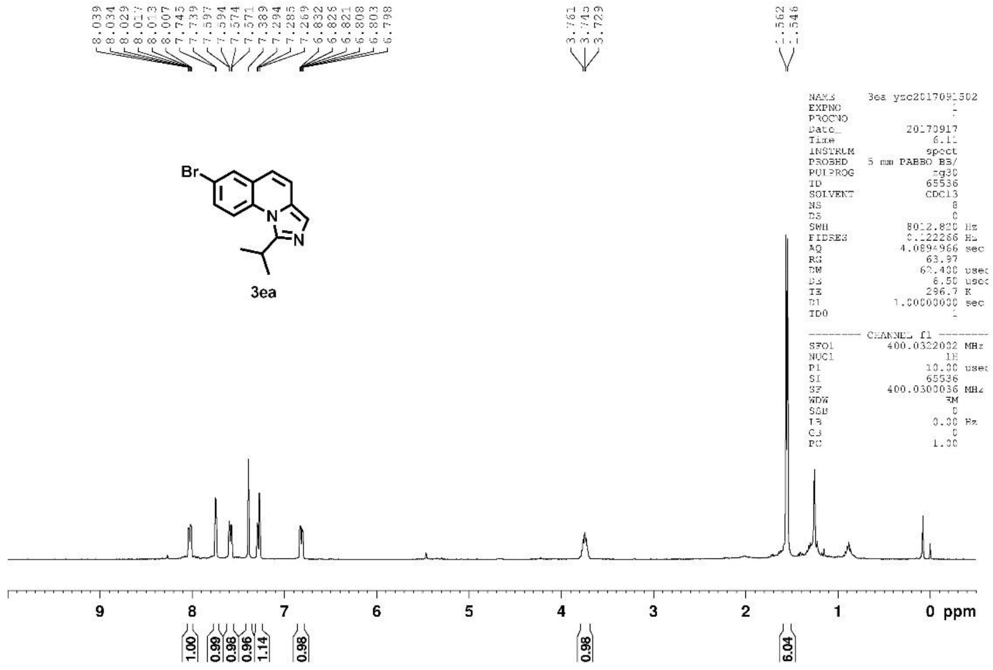
8.039 8.034 8.029 8.017 8.013 8.007 7.745 7.739 7.597 7.594 7.574 7.571 7.389 7.294 7.285 7.269 6.832 6.826 6.821 6.808 6.803 6.798
3.761 3.745 3.729
1.562 1.546
Br
N
N
3ea
NAME 3ea yzc2017091502
EXPNO 1
PROCNO 1
Date_ 20170917
Time 6.11
INSTRUM spect
PROBHD 5 mm PABBO BB/
PULPROG zg30
TD 65536
SOLVENT CDCl3
NS 8
DS 0
SWH 8012.820 Hz
FIDRES 0.122266 Hz
AQ 4.0894966 sec
RG 63.97
DW 62.400 usec
DE 6.50 usec
TE 296.7 K
D1 1.00000000 sec
TD0 1
CHANNEL f1
SFO1 400.0322002 MHz
NUC1 1H
P1 10.00 usec
SI 65536
SF 400.0300036 MHz
WDW EM
SSB 0
LB 0.30 Hz
GB 0
PC 1.00
9 8 7 6 5 4 3 2 1 0 ppm
1.00 0.99 0.98 0.96 1.14 0.98 0.98 6.04

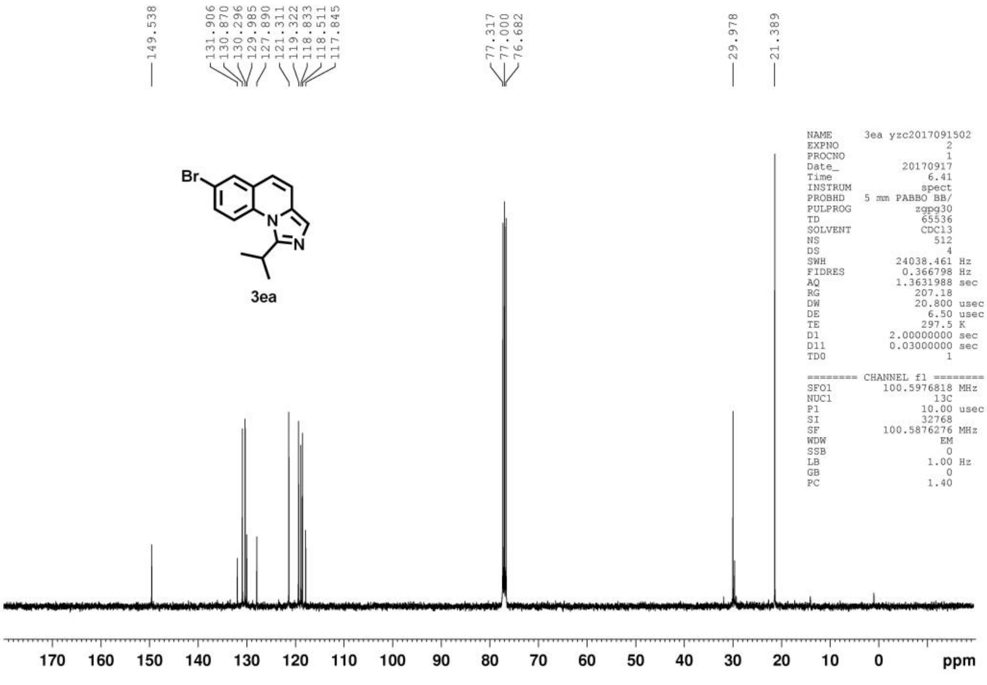
149.538
131.906 130.870 130.296 129.985 127.890 121.311 119.322 118.833 118.511 117.845
77.317 77.000 76.682
29.978
21.389
Br
N
N
3ea
NAME 3ea yzc2017091502
EXPNO 2
PROCNO 1
Date_ 20170917
Time 6.41
INSTRUM spect
PROBHD 5 mm PABBO BB/
PULPROG zgpg30
TD 65536
SOLVENT CDCl3
NS 512
DS 4
SWH 24038.461 Hz
FIDRES 0.366798 Hz
AQ 1.3631988 sec
RG 207.18
DW 20.800 usec
DE 6.50 usec
TE 297.5 K
D1 2.00000000 sec
D11 0.03000000 sec
TD0 1
======== CHANNEL f1 ========
SFO1 100.5976818 MHz
NUC1 13C
P1 10.00 usec
SI 32768
SF 100.5876276 MHz
WDW EM
SSB 0
LB 1.00 Hz
GB 0
PC 1.40
170 160 150 140 130 120 110 100 90 80 70 60 50 40 30 20 10 0 ppm

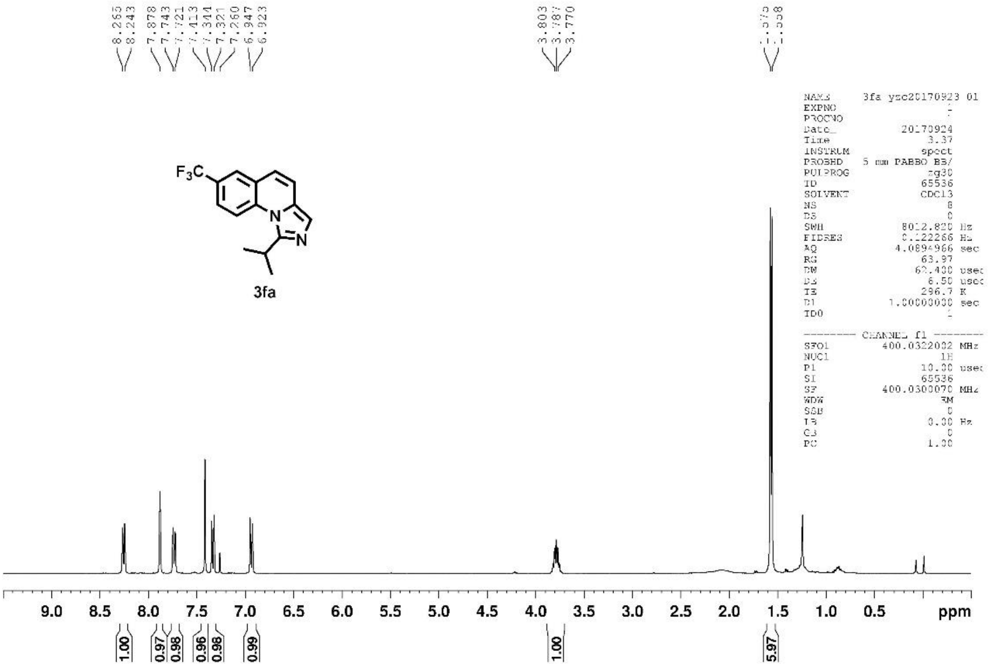
8.265
8.243
7.878
7.743
7.721
7.413
7.344
7.321
7.260
6.947
6.923
3.803
3.787
3.770
1.575
1.558
F3C
N
N
3fa
NAME 3fa yzc20170923 01
EXPNO
PROCNO
Date_ 20170924
Time 3.37
INSTRUM spect
PROBHD 5 mm PABBO BB/
PULPROG zg30
TD 65536
SOLVENT CDCl3
NS 8
DS 0
SWH 8012.820 Hz
FIDRES 0.122266 Hz
AQ 4.0894966 sec
RG 63.97
DW 62.400 usec
DE 6.50 usec
TE 296.7 K
D1 1.00000000 sec
TD0
CHANNEL f1
SFO1 400.0322002 MHz
NUC1 1H
P1 10.00 usec
SI 65536
SF 400.0300070 MHz
WDW EM
SSB 0
LB 0.30 Hz
GB 0
PC 1.00
9.0 8.5 8.0 7.5 7.0 6.5 6.0 5.5 5.0 4.5 4.0 3.5 3.0 2.5 2.0 1.5 1.0 0.5 ppm
1.00
0.97
0.98
0.96
0.98
0.99
1.00
5.97

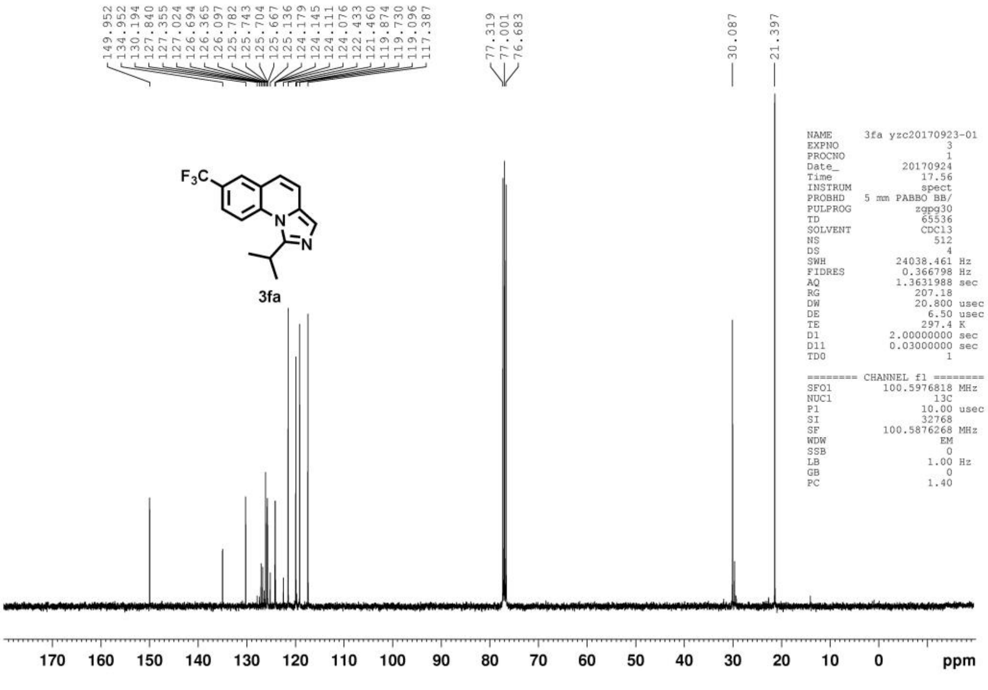
149.952
134.952
130.194
127.840
127.355
127.024
126.694
126.365
126.097
125.782
125.743
125.704
125.667
125.136
124.179
124.145
124.111
124.076
122.433
121.460
119.874
119.730
119.096
117.387
77.319
77.001
76.683
30.087
21.397
F3C
N
N
3fa
NAME 3fa yzc20170923-01
EXPNO 3
PROCNO 1
Date_ 20170924
Time 17.56
INSTRUM spect
PROBHD 5 mm PABBO BB/
PULPROG zgpg30
TD 65536
SOLVENT CDCl3
NS 512
DS 4
SWH 24038.461 Hz
FIDRES 0.366798 Hz
AQ 1.3631988 sec
RG 207.18
DW 20.800 usec
DE 6.50 usec
TE 297.4 K
D1 2.00000000 sec
D11 0.03000000 sec
TD0 1
======== CHANNEL f1 ========
SFO1 100.5976818 MHz
NUC1 13C
P1 10.00 usec
SI 32768
SF 100.5876268 MHz
WDW EM
SSB 0
LB 1.00 Hz
GB 0
PC 1.40
170 160 150 140 130 120 110 100 90 80 70 60 50 40 30 20 10 0 ppm

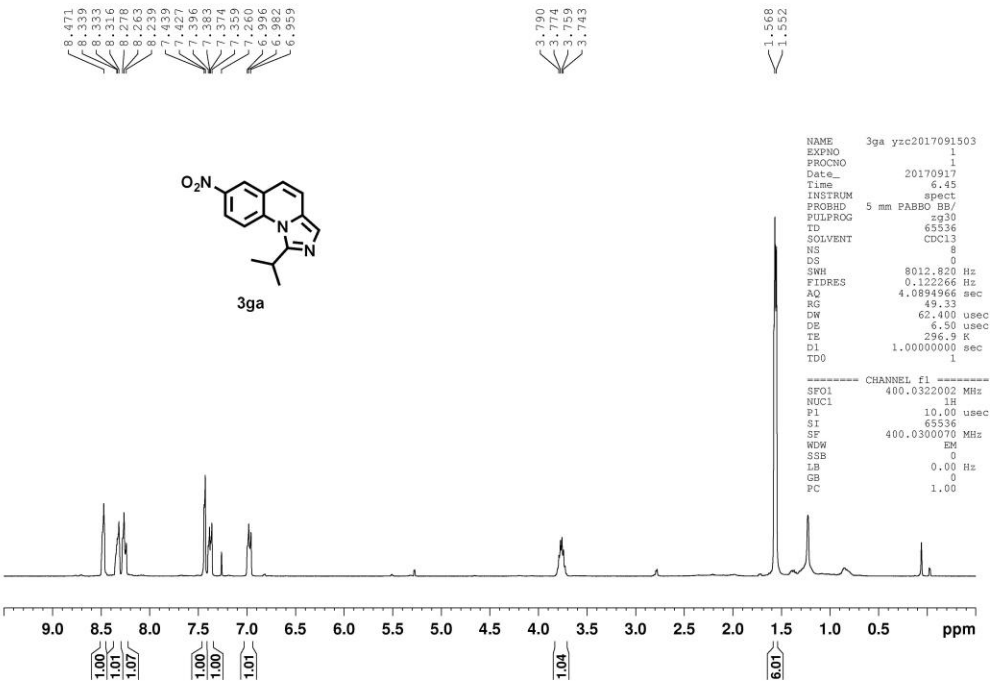
8.471 8.339 8.333 8.316 8.278 8.263 8.239 7.439 7.427 7.396 7.383 7.374 7.359 7.260 6.996 6.982 6.959
3.790 3.774 3.759 3.743
1.568 1.552
O_2N
N
N
3ga
NAME 3ga yzc2017091503
EXPNO 1
PROCNO 1
Date_ 20170917
Time 6.45
INSTRUM spect
PROBHD 5 mm PABBO BB/
PULPROG zg30
TD 65536
SOLVENT CDCl3
NS 8
DS 0
SWH 8012.820 Hz
FIDRES 0.122266 Hz
AQ 4.0894966 sec
RG 49.33
DW 62.400 usec
DE 6.50 usec
TE 296.9 K
D1 1.00000000 sec
TD0 1
======== CHANNEL f1 ========
SFO1 400.0322002 MHz
NUC1 1H
P1 10.00 usec
SI 65536
SF 400.0300070 MHz
WDW EM
SSB 0
LB 0.00 Hz
GB 0
PC 1.00
9.0 8.5 8.0 7.5 7.0 6.5 6.0 5.5 5.0 4.5 4.0 3.5 3.0 2.5 2.0 1.5 1.0 0.5 ppm
1.00 1.01 1.07 1.00 1.00 1.01 1.04 6.01

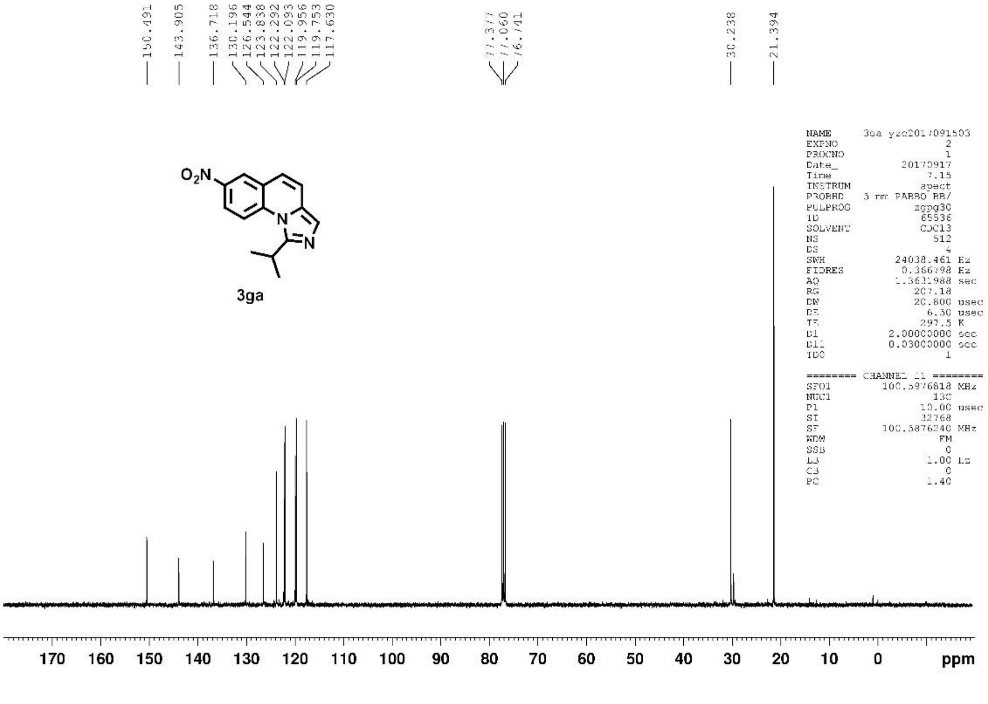
150.491 143.905 136.718 130.196 126.544 123.838 122.292 122.093 119.956 119.753 117.630
30.238 21.394
O_2N
N
N
3ga
170 160 150 140 130 120 110 100 90 80 70 60 50 40 30 20 10 0 ppm

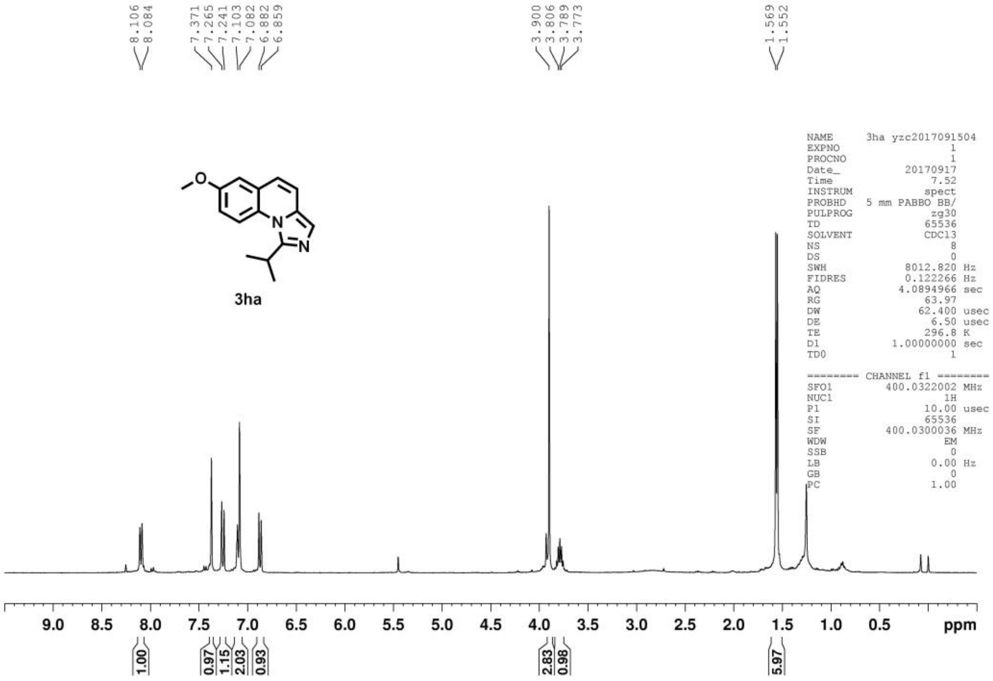
8.106
8.084
7.371
7.265
7.241
7.103
7.082
6.882
6.859
3.900
3.806
3.789
3.773
1.569
1.552
3ha
NAME 3ha yzc2017091504
EXPNO 1
PROCNO 1
Date_ 20170917
Time 7.52
INSTRUM spect
PROBHD 5 mm PABBO BB/
PULPROG zg30
TD 65536
SOLVENT CDCl3
NS 8
DS 0
SWH 8012.820 Hz
FIDRES 0.122266 Hz
AQ 4.0894966 sec
RG 63.97
DW 62.400 usec
DE 6.50 usec
TE 296.8 K
D1 1.00000000 sec
TD0 1
======== CHANNEL f1 ========
SFO1 400.0322002 MHz
NUC1 1H
P1 10.00 usec
SI 65536
SF 400.0300036 MHz
WDW EM
SSB 0
LB 0.00 Hz
GB 0
PC 1.00
9.0 8.5 8.0 7.5 7.0 6.5 6.0 5.5 5.0 4.5 4.0 3.5 3.0 2.5 2.0 1.5 1.0 0.5 ppm
1.00
0.97
1.15
2.03
0.93
2.83
0.98
5.97

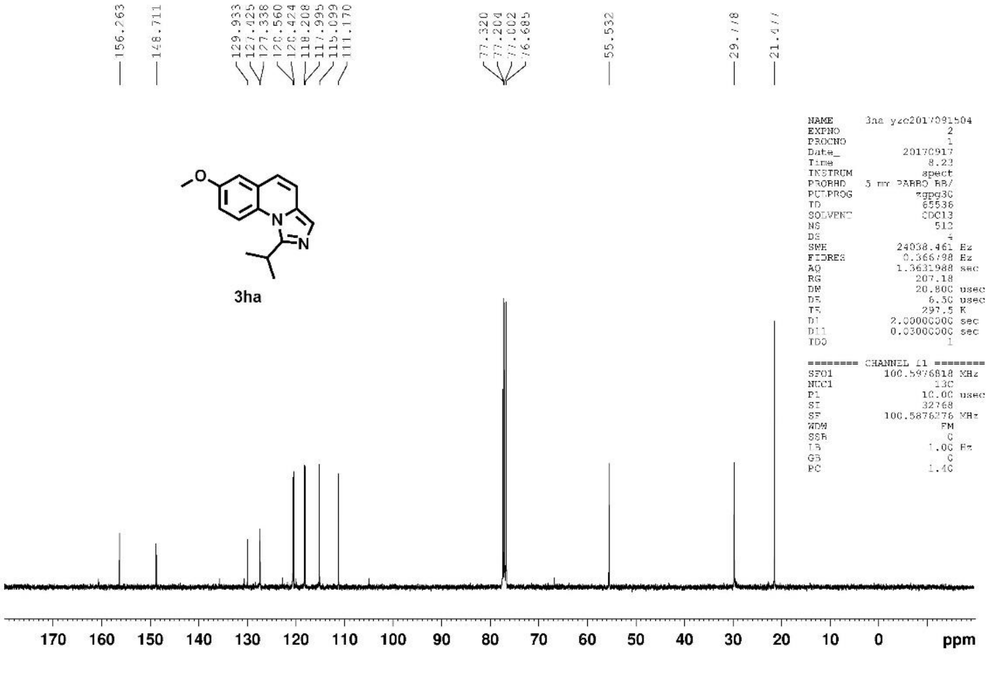
156.263
148.711
129.933
127.425
127.338
120.560
120.424
118.208
117.995
115.099
111.170
77.320
77.204
77.002
76.685
55.532
29.778
21.477
3ha
170 160 150 140 130 120 110 100 90 80 70 60 50 40 30 20 10 0 ppm

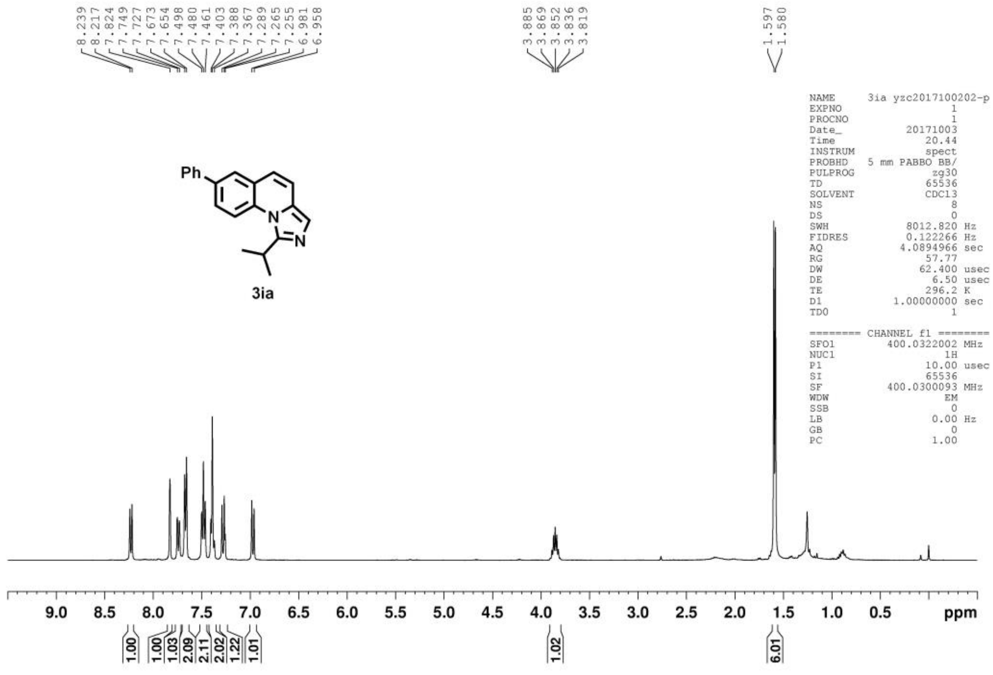
8.239
8.217
7.824
7.749
7.727
7.673
7.654
7.498
7.480
7.461
7.403
7.388
7.367
7.289
7.265
7.255
6.981
6.958
3.885
3.869
3.852
3.836
3.819
1.597
1.580
Ph
N
N
3ia
NAME 3ia yzc2017100202-p
EXPNO 1
PROCNO 1
Date_ 20171003
Time 20.44
INSTRUM spect
PROBHD 5 mm PABBO BB/
PULPROG zg30
TD 65536
SOLVENT CDCl3
NS 8
DS 0
SWH 8012.820 Hz
FIDRES 0.122266 Hz
AQ 4.0894966 sec
RG 57.77
DW 62.400 usec
DE 6.50 usec
TE 296.2 K
D1 1.00000000 sec
TD0 1
======== CHANNEL f1 ========
SFO1 400.0322002 MHz
NUC1 1H
P1 10.00 usec
SI 65536
SF 400.0300093 MHz
WDW EM
SSB 0
LB 0.00 Hz
GB 0
PC 1.00
9.0 8.5 8.0 7.5 7.0 6.5 6.0 5.5 5.0 4.5 4.0 3.5 3.0 2.5 2.0 1.5 1.0 0.5 ppm
1.00
1.00
1.03
2.09
2.11
2.02
1.22
1.01
1.02
6.01

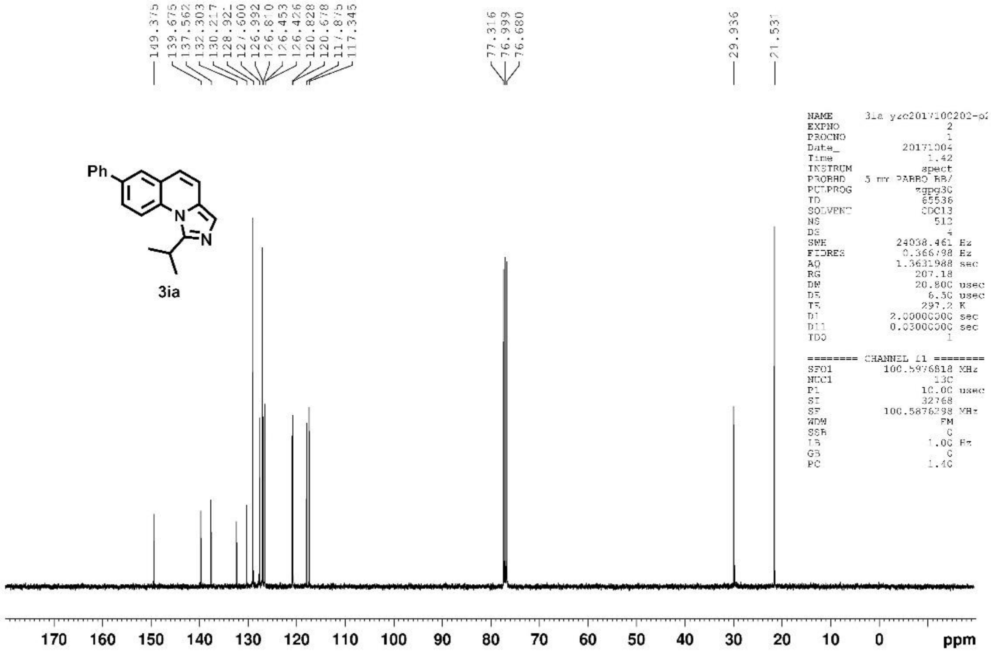
149.375
139.675
137.562
132.303
130.217
128.921
127.600
126.992
126.810
126.453
126.426
120.828
120.678
117.875
117.345
77.316
76.999
76.680
29.936
21.531
Ph
N
N
3ia
NAME 3ia yzc2017100202-p2
EXPNO 2
PROCNO 1
Date_ 20171004
Time 1.42
INSTRUM spect
PROBHD 5 mm PABBO BB/
PULPROG zgpg30
TD 65536
SOLVENT CDCl3
NS 512
DS 4
SWH 24038.461 Hz
FIDRES 0.366798 Hz
AQ 1.3631988 sec
RG 207.18
DW 20.800 usec
DE 6.50 usec
TE 297.2 K
D1 2.00000000 sec
D11 0.03000000 sec
TD0 1
======== CHANNEL f1 ========
SFO1 100.5976818 MHz
NUC1 13C
P1 10.00 usec
SI 32768
SF 100.5876298 MHz
WDW EM
SSB 0
LB 1.00 Hz
GB 0
PC 1.40
170 160 150 140 130 120 110 100 90 80 70 60 50 40 30 20 10 0 ppm

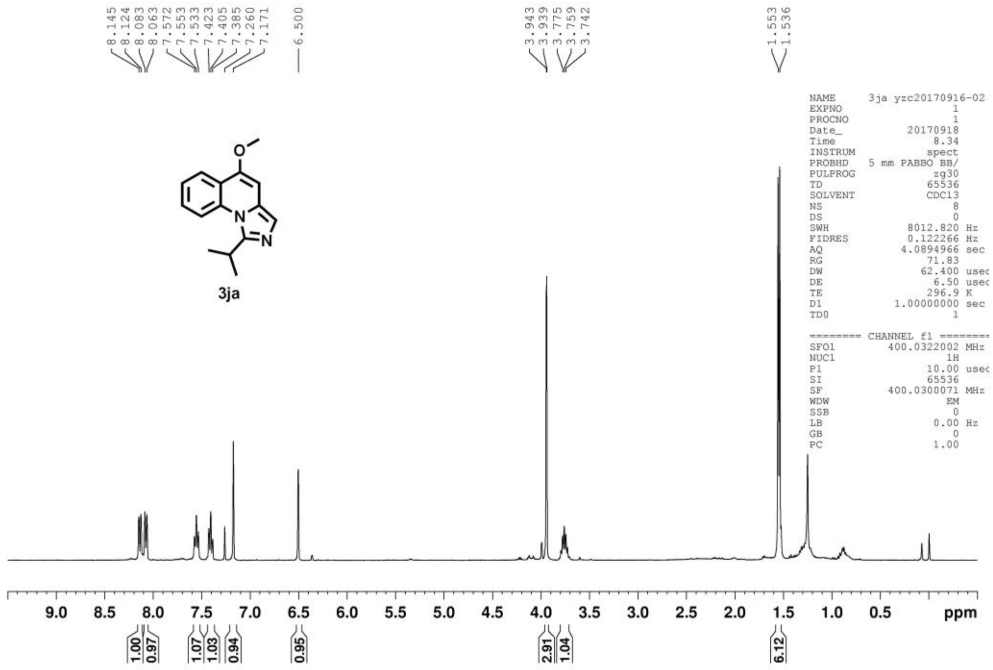
3ja
ppm

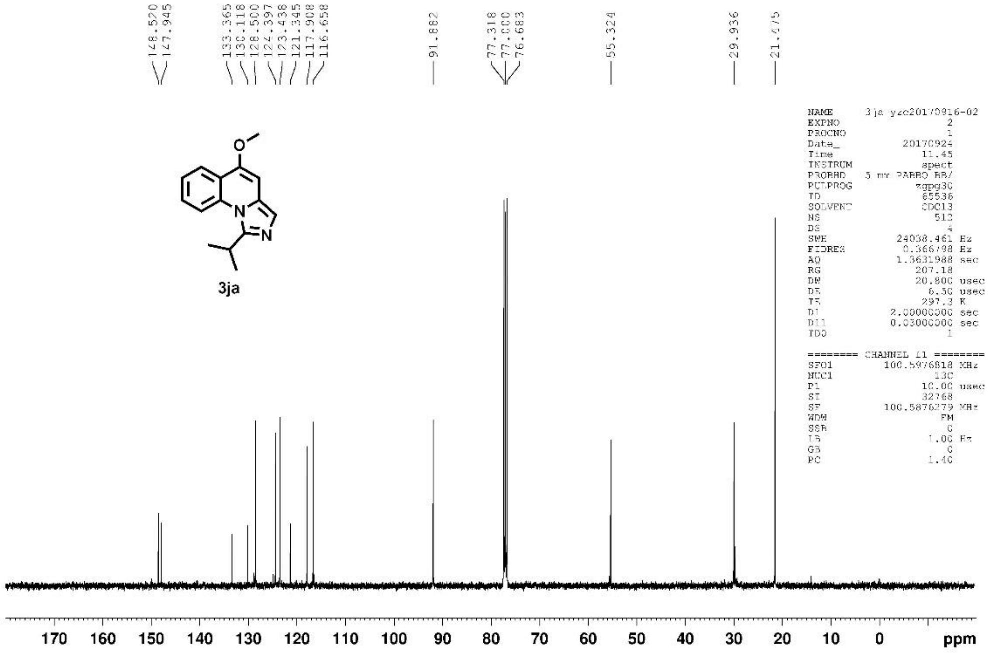
3ja
ppm

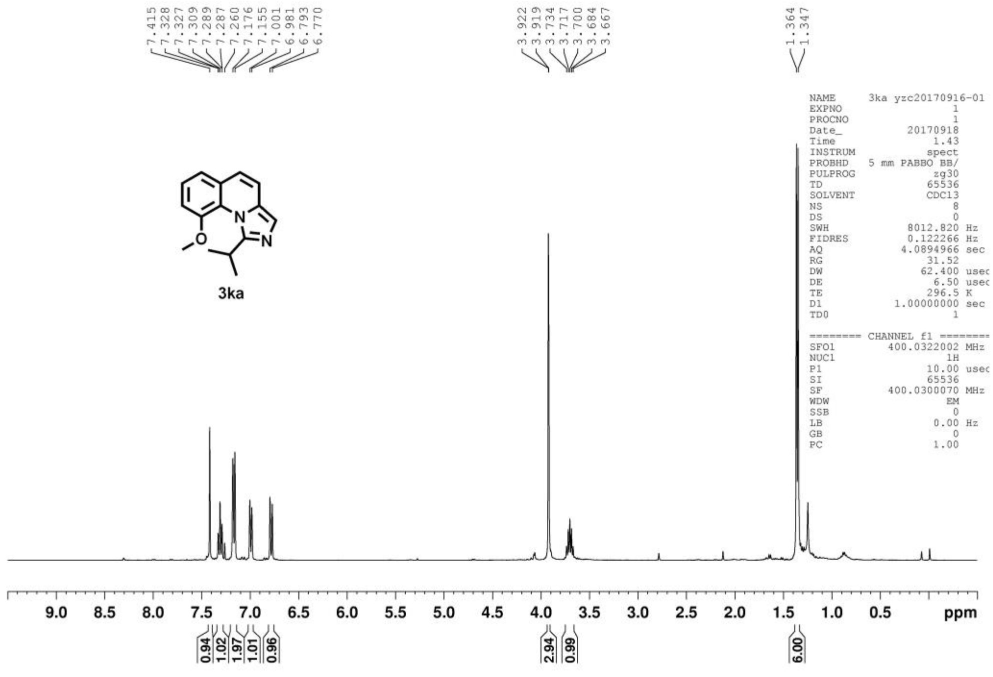
7.415
7.328
7.327
7.309
7.289
7.287
7.260
7.176
7.155
7.001
6.981
6.793
6.770
3.922
3.919
3.734
3.717
3.700
3.684
3.667
1.364
1.347
3ka
NAME 3ka yzc20170916-01
EXPNO 1
PROCNO 1
Date_ 20170918
Time 1.43
INSTRUM spect
PROBHD 5 mm PABBO BB/
PULPROG zg30
TD 65536
SOLVENT CDCl3
NS 8
DS 0
SWH 8012.820 Hz
FIDRES 0.122266 Hz
AQ 4.0894966 sec
RG 31.52
DW 62.400 usec
DE 6.50 usec
TE 296.5 K
D1 1.00000000 sec
TD0 1
======== CHANNEL f1 ========
SFO1 400.0322002 MHz
NUC1 1H
P1 10.00 usec
SI 65536
SF 400.0300070 MHz
WDW EM
SSB 0
LB 0.00 Hz
GB 0
PC 1.00
9.0 8.5 8.0 7.5 7.0 6.5 6.0 5.5 5.0 4.5 4.0 3.5 3.0 2.5 2.0 1.5 1.0 0.5 ppm
0.94
1.02
1.97
1.01
0.96
2.94
0.99
6.00

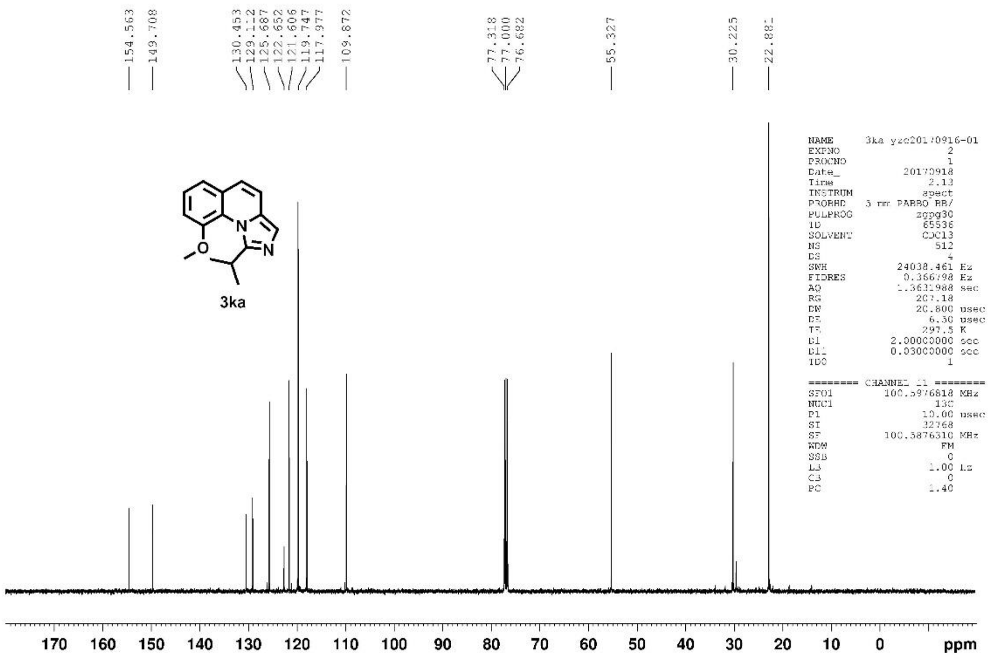
154.563
149.708
130.453
129.122
125.687
122.652
121.606
119.747
117.977
109.872
77.318
77.000
76.682
55.327
30.225
22.881
3ka
NAME 3ka yzc20170916-01
EXPNO 2
PROCNO 1
Date_ 20170918
SOLVENT CDCl3
NS 512
======== CHANNEL f1 ========
NUC1 13C
170 160 150 140 130 120 110 100 90 80 70 60 50 40 30 20 10 0 ppm

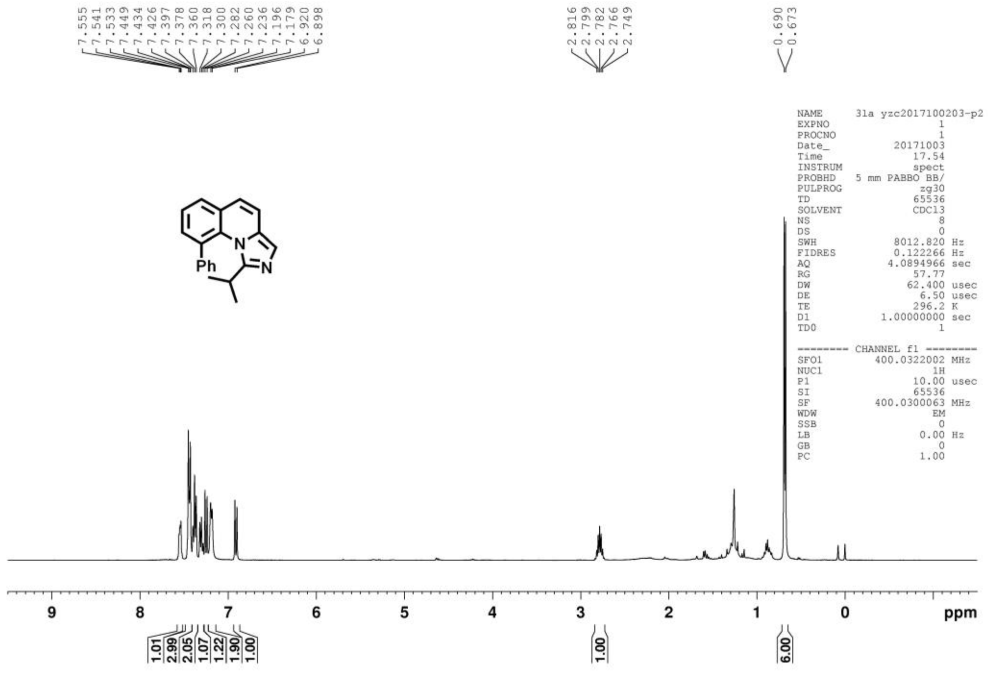
7.555 7.541 7.533 7.449 7.434 7.426 7.397 7.378 7.360 7.318 7.300 7.282 7.260 7.236 7.196 7.179 6.920 6.898
2.816 2.799 2.782 2.766 2.749
0.690 0.673
Ph
N
ppm
1.01 2.99 2.05 1.07 1.22 1.90 1.00
1.00
6.00

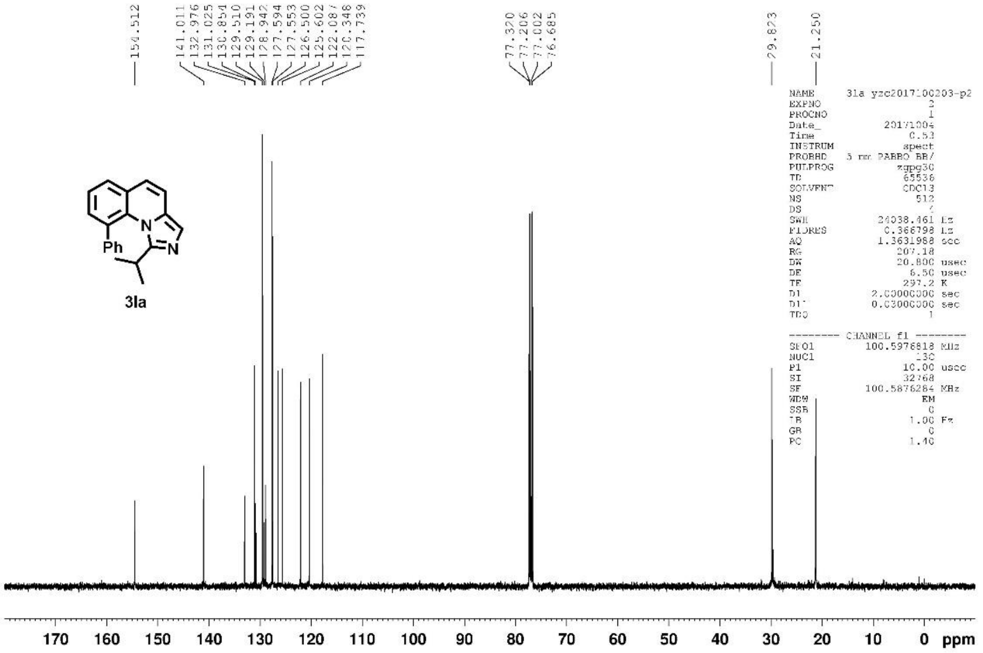
154.512
141.011 132.976 131.025 130.854 129.510 129.191 128.942 127.594 127.553 126.500 125.602 122.087 120.348 117.739
77.320 77.206 77.002 76.685
29.823
21.250
Ph
N
3la
ppm

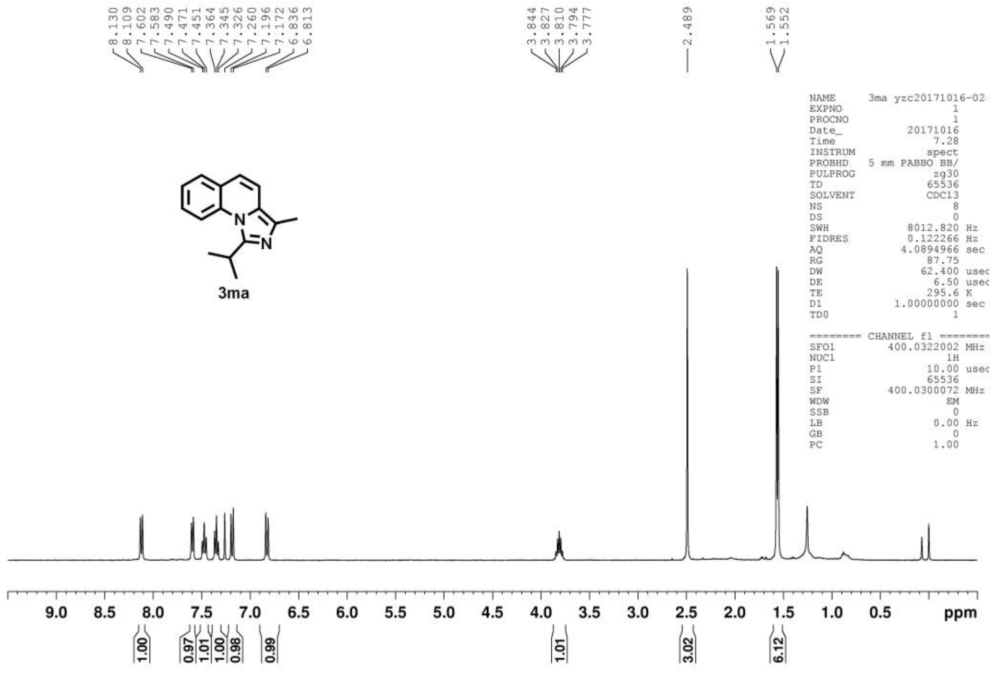
8.130
8.109
7.602
7.583
7.490
7.471
7.451
7.364
7.345
7.326
7.260
7.196
7.172
6.836
6.813
3.844
3.827
3.810
3.794
3.777
2.489
1.569
1.552
3ma
NAME 3ma yzc20171016-02
EXPNO 1
PROCNO 1
Date_ 20171016
Time 7.28
INSTRUM spect
PROBHD 5 mm PABBO BB/
PULPROG zg30
TD 65536
SOLVENT CDCl3
NS 8
DS 0
SWH 8012.820 Hz
FIDRES 0.122266 Hz
AQ 4.0894966 sec
RG 87.75
DW 62.400 usec
DE 6.50 usec
TE 295.6 K
D1 1.00000000 sec
TD0 1
======== CHANNEL f1 ========
SFO1 400.0322002 MHz
NUC1 1H
P1 10.00 usec
SI 65536
SF 400.0300072 MHz
WDW EM
SSB 0
LB 0.00 Hz
GB 0
PC 1.00
9.0 8.5 8.0 7.5 7.0 6.5 6.0 5.5 5.0 4.5 4.0 3.5 3.0 2.5 2.0 1.5 1.0 0.5 ppm
1.00
0.97
1.01
1.00
0.98
0.99
1.01
3.02
6.12

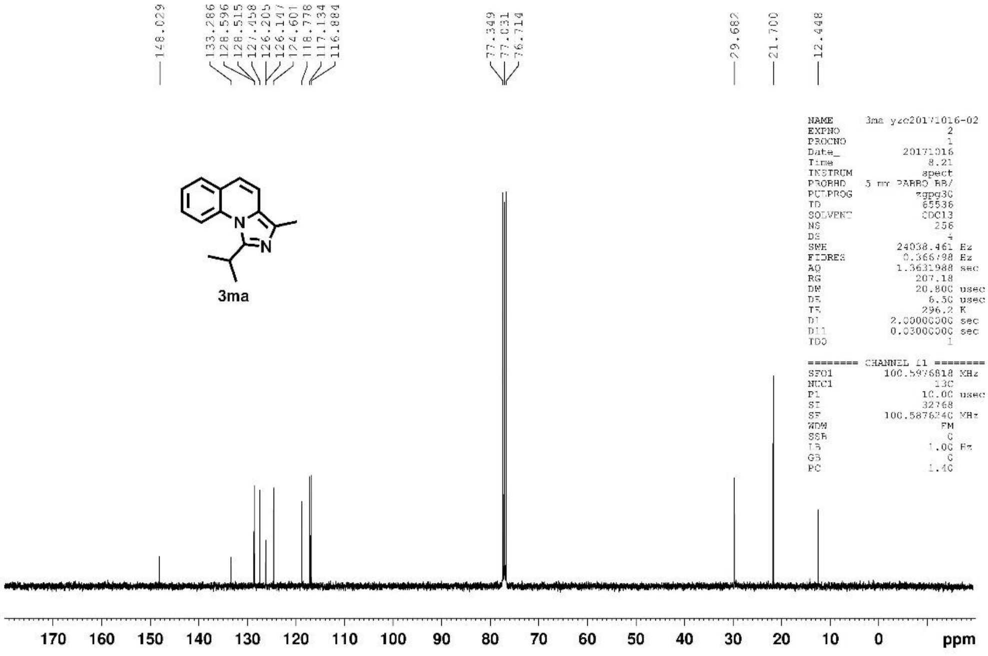
148.029
133.286
128.596
128.515
127.458
126.205
126.147
124.601
118.778
117.134
116.884
77.349
77.031
76.714
29.682
21.700
12.448
3ma
NAME 3ma yzc20171016-02
EXPNO 2
PROCNO 1
Date_ 20171016
Time 8.21
INSTRUM spect
PROBHD 5 mm PABBO BB/
PULPROG zgpg30
TD 65536
SOLVENT CDCl3
NS 256
DS 4
SWH 24038.461 Hz
FIDRES 0.366798 Hz
AQ 1.3631988 sec
RG 207.18
DW 20.800 usec
DE 6.50 usec
TE 296.2 K
D1 2.00000000 sec
D11 0.03000000 sec
TD0 1
======== CHANNEL f1 ========
SFO1 100.5976818 MHz
NUC1 13C
P1 10.00 usec
SI 32768
SF 100.5876240 MHz
WDW EM
SSB 0
LB 1.00 Hz
GB 0
PC 1.40
170 160 150 140 130 120 110 100 90 80 70 60 50 40 30 20 10 0 ppm

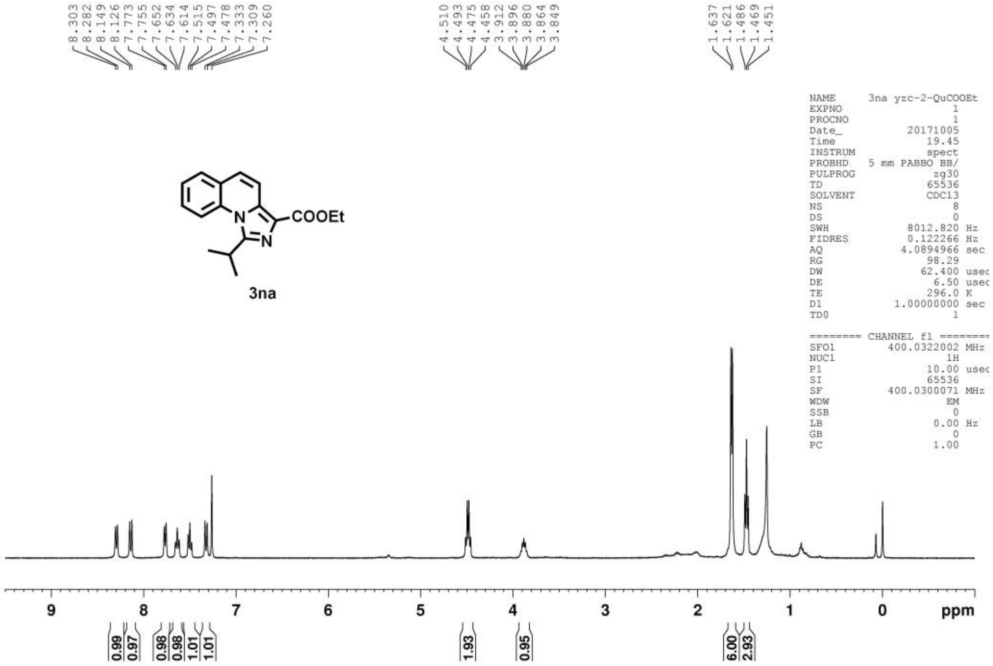
8.303
8.282
8.149
8.126
7.773
7.755
7.652
7.634
7.614
7.515
7.497
7.478
7.333
7.309
7.260
4.510
4.493
4.475
4.458
3.912
3.896
3.880
3.864
3.849
1.637
1.621
1.486
1.469
1.451
COOEt
N
N
3na
NAME 3na yzc-2-QuCOOEt
EXPNO 1
PROCNO 1
Date_ 20171005
Time 19.45
INSTRUM spect
PROBHD 5 mm PABBO BB/
PULPROG zg30
TD 65536
SOLVENT CDCl3
NS 8
DS 0
SWH 8012.820 Hz
FIDRES 0.122266 Hz
AQ 4.0894966 sec
RG 98.29
DW 62.400 usec
DE 6.50 usec
TE 296.0 K
D1 1.00000000 sec
TD0 1
======== CHANNEL f1 ========
SFO1 400.0322002 MHz
NUC1 1H
P1 10.00 usec
SI 65536
SF 400.0300071 MHz
WDW EM
SSB 0
LB 0.00 Hz
GB 0
PC 1.00
9
8
7
6
5
4
3
2
1
0
ppm
0.99
0.97
0.98
0.98
1.01
1.01
1.93
0.95
6.00
2.93

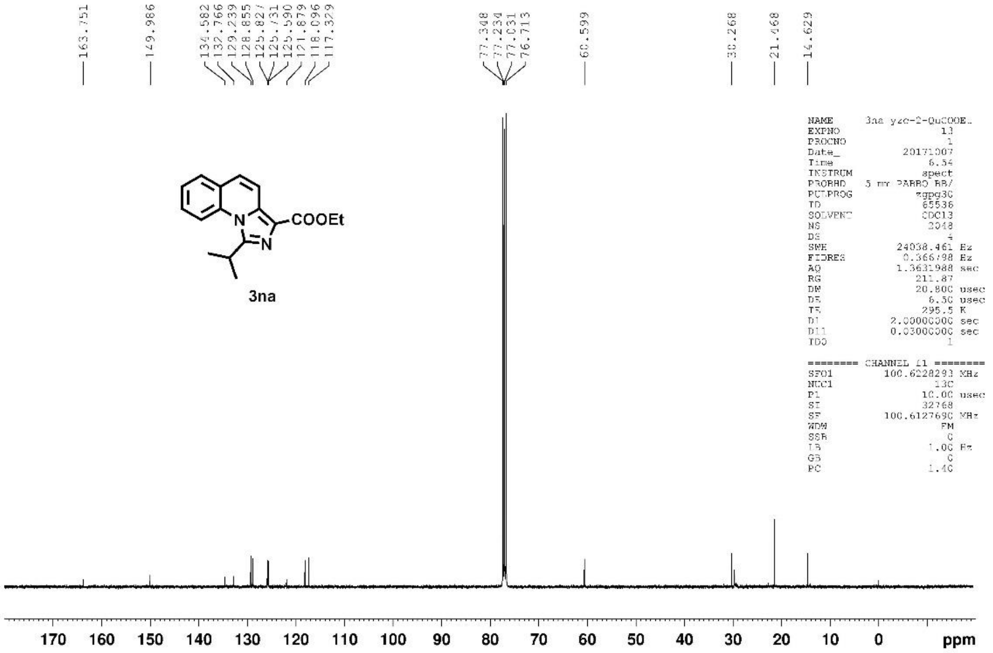
163.751
149.986
134.582
132.766
129.239
128.855
125.827
125.731
125.590
121.879
118.096
117.329
77.348
77.234
77.031
76.713
60.599
30.268
21.468
14.629
COOEt
N
N
3na
NAME 3na yzc-2-QuCOOEt
EXPNO 13
PROCNO 1
Date_ 20171007
Time 6.34
INSTRUM spect
PULPROG zgpg30
TD 65536
SOLVENT CDCl3
NS 2048
DS 4
RG 211.87
TE 295.5 K
======== CHANNEL f1 ========
NUC1 13C
SI 32768
WDW EM
170
160
150
140
130
120
110
100
90
80
70
60
50
40
30
20
10
0
ppm

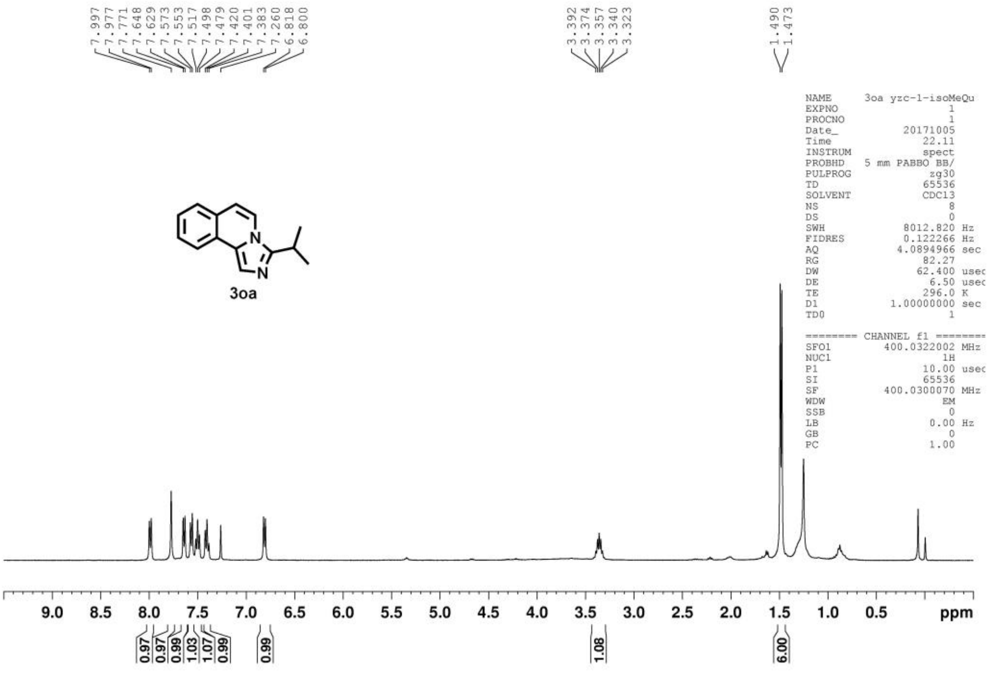
7.997
7.977
7.771
7.648
7.629
7.573
7.553
7.517
7.498
7.479
7.420
7.401
7.383
7.260
6.818
6.800
3.392
3.374
3.357
3.340
3.323
1.490
1.473
3oa
N
N
NAME 3oa yzc-1-isoMeQu
EXPNO 1
PROCNO 1
Date_ 20171005
Time 22.11
INSTRUM spect
PROBHD 5 mm PABBO BB/
PULPROG zg30
TD 65536
SOLVENT CDCl3
NS 8
DS 0
SWH 8012.820 Hz
FIDRES 0.122266 Hz
AQ 4.0894966 sec
RG 82.27
DW 62.400 usec
DE 6.50 usec
TE 296.0 K
D1 1.00000000 sec
TD0 1
======== CHANNEL f1 ========
SFO1 400.0322002 MHz
NUC1 1H
P1 10.00 usec
SI 65536
SF 400.0300070 MHz
WDW EM
SSB 0
LB 0.00 Hz
GB 0
PC 1.00
9.0 8.5 8.0 7.5 7.0 6.5 6.0 5.5 5.0 4.5 4.0 3.5 3.0 2.5 2.0 1.5 1.0 0.5 ppm
0.97
0.97
0.99
1.03
1.07
0.99
0.99
1.08
6.00

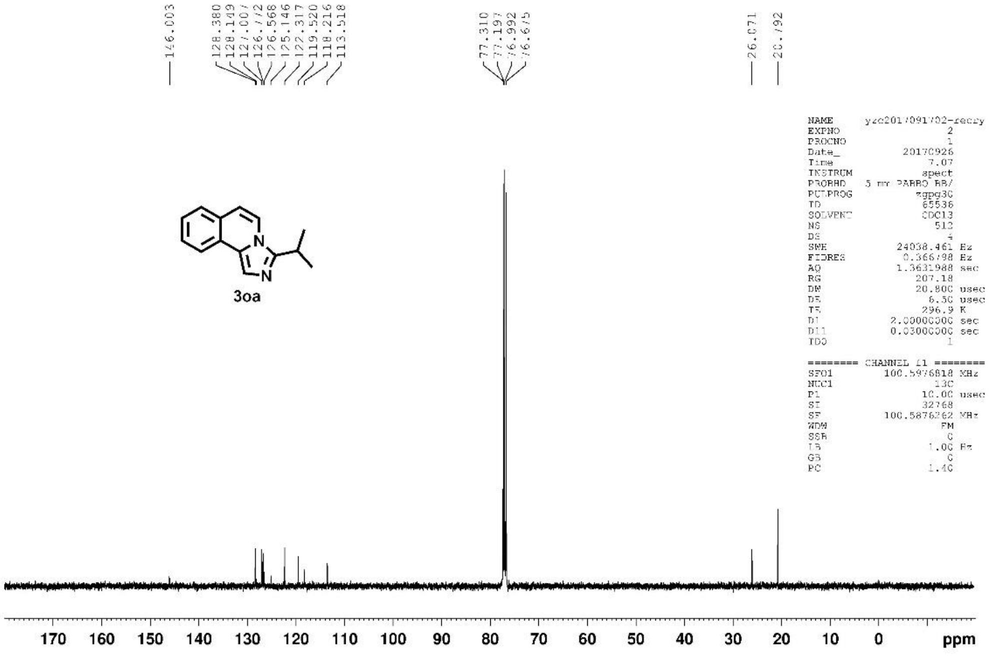
146.003
128.380
128.149
127.007
126.772
126.568
125.146
122.317
119.520
118.216
113.518
77.310
77.197
76.992
76.675
26.071
20.792
3oa
N
N
NAME
EXPNO 2
PROCNO 1
Date_ 20170926
Time 7.07
INSTRUM spect
SOLVENT CDCl3
NS 512
DS 4
SWH 24038.461 Hz
RG 207.18
TE 296.9 K
TD0 1
======== CHANNEL f1 ========
NUC1 13C
SI 32768
WDW EM
SSB 0
LB 1.00 Hz
GB 0
PC 1.40
170 160 150 140 130 120 110 100 90 80 70 60 50 40 30 20 10 0 ppm

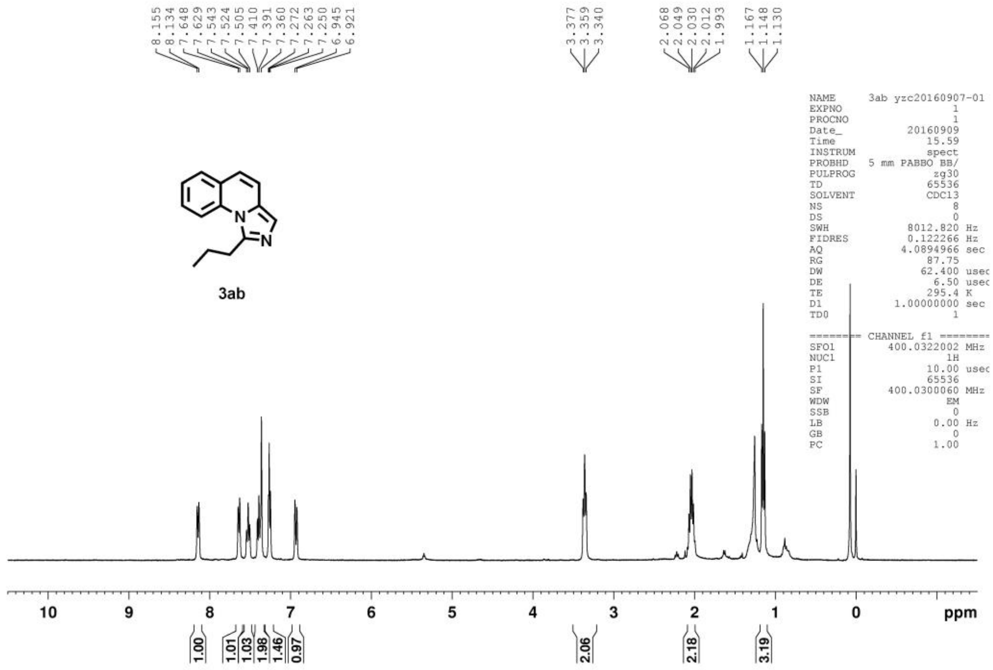
8.155
8.134
7.648
7.629
7.543
7.524
7.505
7.410
7.391
7.360
7.272
7.263
7.250
6.945
6.921
3.377
3.359
3.340
2.068
2.049
2.030
2.012
1.993
1.167
1.148
1.130
N
N
3ab
NAME 3ab yzc20160907-01
EXPNO 1
PROCNO 1
Date_ 20160909
Time 15.59
INSTRUM spect
PROBHD 5 mm PABBO BB/
PULPROG zg30
TD 65536
SOLVENT CDCl3
NS 8
DS 0
SWH 8012.820 Hz
FIDRES 0.122266 Hz
AQ 4.0894966 sec
RG 87.75
DW 62.400 usec
DE 6.50 usec
TE 295.4 K
D1 1.00000000 sec
TD0 1
======== CHANNEL f1 ========
SFO1 400.0322002 MHz
NUC1 1H
P1 10.00 usec
SI 65536
SF 400.0300060 MHz
WDW EM
SSB 0
LB 0.00 Hz
GB 0
PC 1.00
10
9
8
7
6
5
4
3
2
1
0
ppm
1.00
1.01
1.03
1.98
1.46
0.97
2.06
2.18
3.19

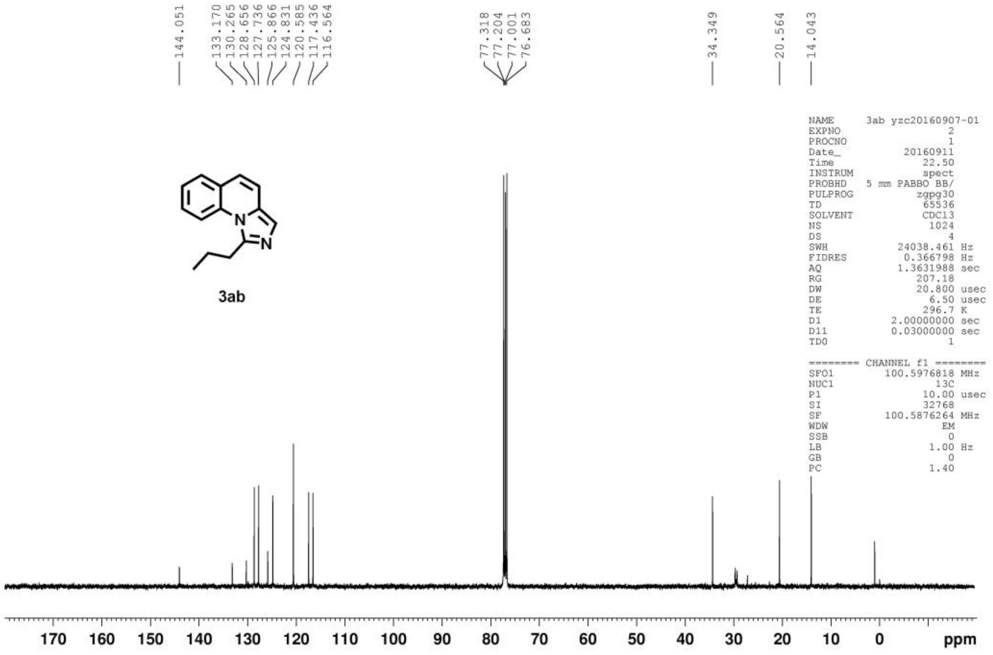
144.051
133.170
130.265
128.656
127.736
125.866
124.831
120.585
117.436
116.564
77.318
77.204
77.001
76.683
34.349
20.564
14.043
N
N
3ab
NAME 3ab yzc20160907-01
EXPNO 2
PROCNO 1
Date_ 20160911
Time 22.50
INSTRUM spect
PROBHD 5 mm PABBO BB/
PULPROG zgpg30
TD 65536
SOLVENT CDCl3
NS 1024
DS 4
SWH 24038.461 Hz
FIDRES 0.366798 Hz
AQ 1.3631988 sec
RG 207.18
DW 20.800 usec
DE 6.50 usec
TE 296.7 K
D1 2.00000000 sec
D11 0.03000000 sec
TD0 1
======== CHANNEL f1 ========
SFO1 100.5976818 MHz
NUC1 13C
P1 10.00 usec
SI 32768
SF 100.5876264 MHz
WDW EM
SSB 0
LB 1.00 Hz
GB 0
PC 1.40
170
160
150
140
130
120
110
100
90
80
70
60
50
40
30
20
10
0
ppm

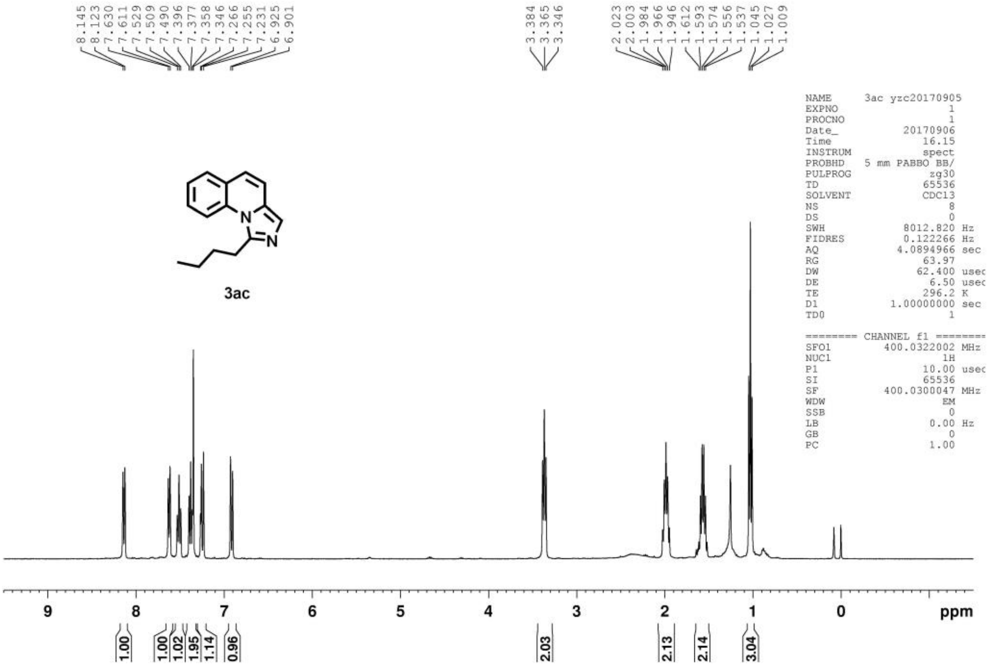
8.145
8.123
7.630
7.611
7.529
7.509
7.490
7.396
7.377
7.358
7.346
7.266
7.255
7.231
6.925
6.901
3.384
3.365
3.346
2.023
2.003
1.984
1.966
1.946
1.612
1.593
1.574
1.556
1.537
1.045
1.027
1.009
N
N
3ac
NAME 3ac yzc20170905
EXPNO 1
PROCNO 1
Date_ 20170906
Time 16.15
INSTRUM spect
PROBHD 5 mm PABBO BB/
PULPROG zg30
TD 65536
SOLVENT CDCl3
NS 8
DS 0
SWH 8012.820 Hz
FIDRES 0.122266 Hz
AQ 4.0894966 sec
RG 63.97
DW 62.400 usec
DE 6.50 usec
TE 296.2 K
D1 1.00000000 sec
TD0 1
======== CHANNEL f1 ========
SFO1 400.0322002 MHz
NUC1 1H
P1 10.00 usec
SI 65536
SF 400.0300047 MHz
WDW EM
SSB 0
LB 0.00 Hz
GB 0
PC 1.00
9 8 7 6 5 4 3 2 1 0 ppm
1.00
1.00
1.02
1.95
1.14
0.96
2.03
2.13
2.14
3.04

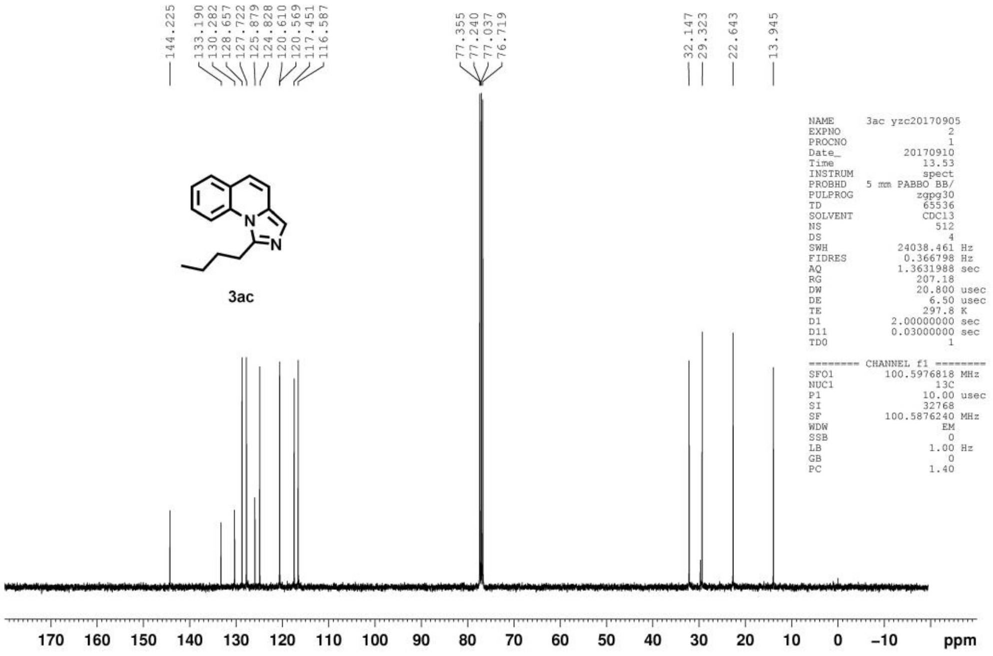
144.225
133.190
130.282
128.657
127.722
125.879
124.828
120.610
120.569
117.451
116.587
77.355
77.240
77.037
76.719
32.147
29.323
22.643
13.945
N
N
3ac
NAME 3ac yzc20170905
EXPNO 2
PROCNO 1
Date_ 20170910
Time 13.53
INSTRUM spect
PROBHD 5 mm PABBO BB/
PULPROG zgpg30
TD 65536
SOLVENT CDCl3
NS 512
DS 4
SWH 24038.461 Hz
FIDRES 0.366798 Hz
AQ 1.3631988 sec
RG 207.18
DW 20.800 usec
DE 6.50 usec
TE 297.8 K
D1 2.00000000 sec
D11 0.03000000 sec
TD0 1
======== CHANNEL f1 ========
SFO1 100.5976818 MHz
NUC1 13C
P1 10.00 usec
SI 32768
SF 100.5876240 MHz
WDW EM
SSB 0
LB 1.00 Hz
GB 0
PC 1.40
170 160 150 140 130 120 110 100 90 80 70 60 50 40 30 20 10 0 −10 ppm

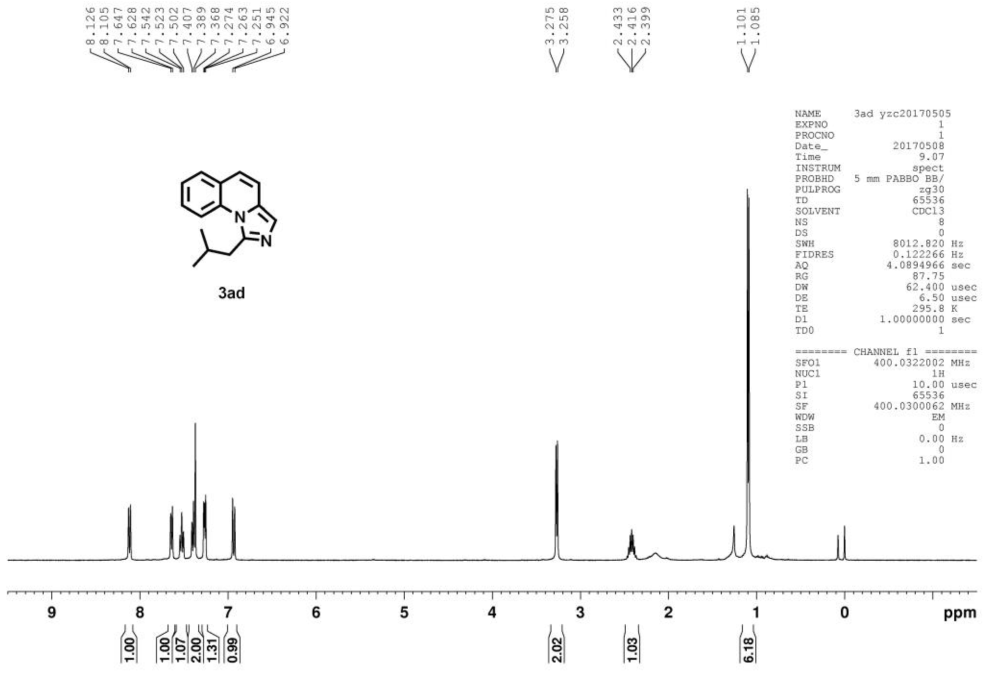
8.126
8.105
7.647
7.628
7.542
7.523
7.502
7.407
7.389
7.368
7.274
7.263
7.251
6.945
6.922
3.275
3.258
2.433
2.416
2.399
1.101
1.085
N
N
3ad
NAME 3ad yzc20170505
EXPNO 1
PROCNO 1
Date_ 20170508
Time 9.07
INSTRUM spect
PROBHD 5 mm PABBO BB/
PULPROG zg30
TD 65536
SOLVENT CDCl3
NS 8
DS 0
SWH 8012.820 Hz
FIDRES 0.122266 Hz
AQ 4.0894966 sec
RG 87.75
DW 62.400 usec
DE 6.50 usec
TE 295.8 K
D1 1.00000000 sec
TD0 1
======== CHANNEL f1 ========
SFO1 400.0322002 MHz
NUC1 1H
P1 10.00 usec
SI 65536
SF 400.0300062 MHz
WDW EM
SSB 0
LB 0.00 Hz
GB 0
PC 1.00
9 8 7 6 5 4 3 2 1 0 ppm
1.00
1.00
1.07
2.00
1.31
0.99
2.02
1.03
6.18

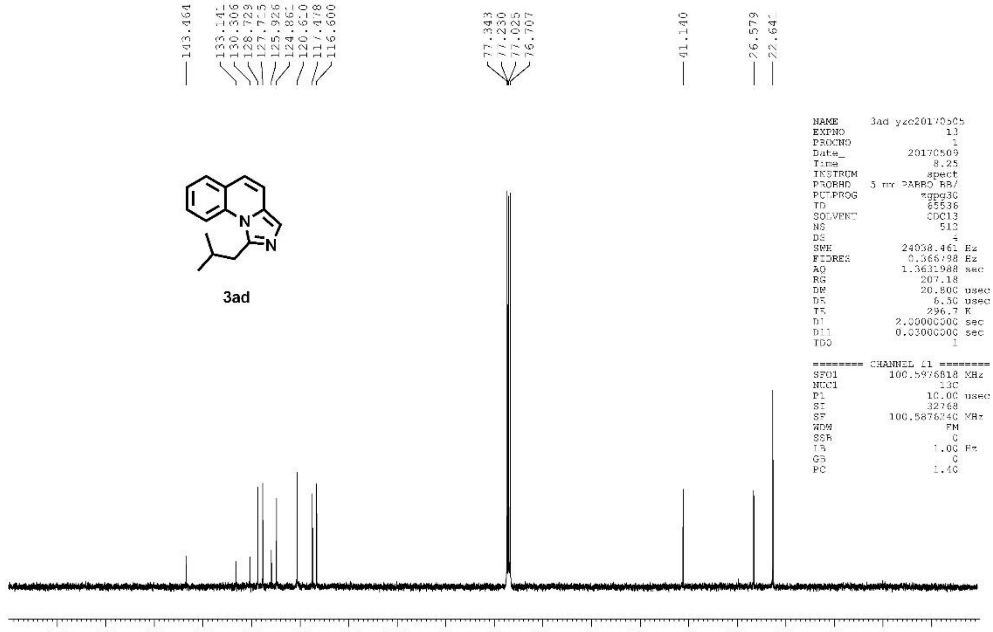
143.464
133.141
130.306
128.729
127.755
125.926
124.862
120.620
117.478
116.600
77.343
77.230
77.025
76.707
41.140
26.579
22.641
N
N
3ad
NAME 3ad yzc20170505
EXPNO 13
PROCNO 1
Date_ 20170509
Time 8.25
INSTRUM spect
PROBHD 5 mm PABBO BB/
PULPROG zgpg30
TD 65536
SOLVENT CDCl3
NS 512
DS 4
SWH 24038.461 Hz
FIDRES 0.366798 Hz
AQ 1.3631988 sec
RG 207.18
DW 20.800 usec
DE 6.50 usec
TE 296.7 K
D1 2.00000000 sec
D11 0.03000000 sec
TD0 1
======== CHANNEL f1 ========
SFO1 100.5976818 MHz
NUC1 13C
P1 10.00 usec
SI 32768
SF 100.5876240 MHz
WDW EM
SSB 0
LB 1.00 Hz
GB 0
PC 1.40
170 160 150 140 130 120 110 100 90 80 70 60 50 40 30 20 10 0 ppm

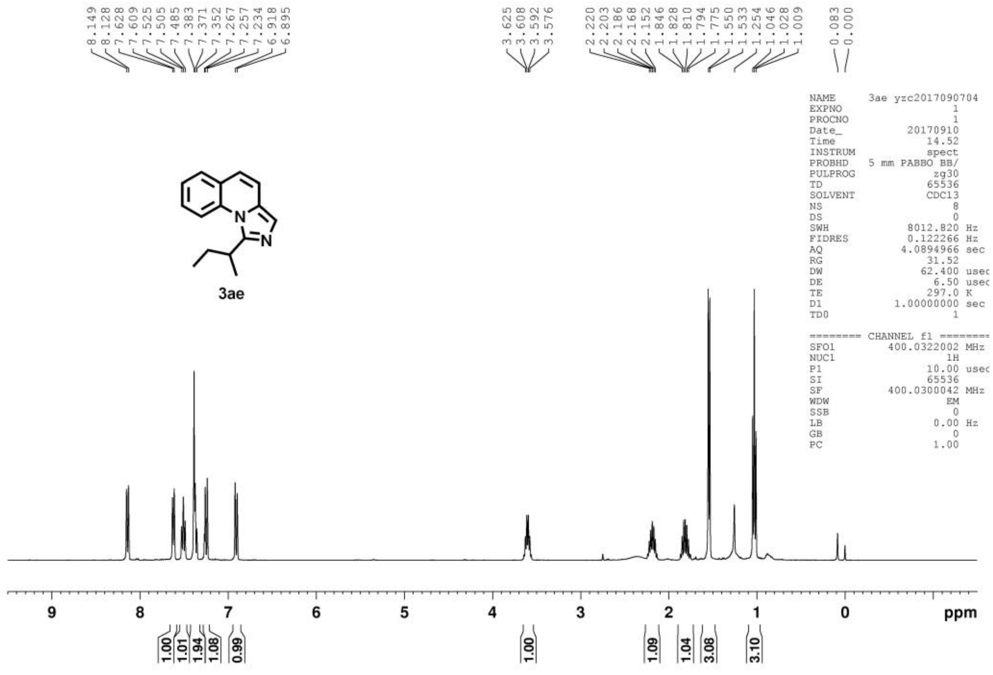
8.149
8.128
7.628
7.609
7.525
7.505
7.485
7.383
7.371
7.352
7.267
7.257
7.234
6.918
6.895
3.625
3.608
3.592
3.576
2.220
2.203
2.186
2.168
2.152
1.846
1.828
1.810
1.794
1.775
1.550
1.533
1.254
1.046
1.028
1.009
0.083
0.000
3ae
NAME 3ae yzc2017090704
EXPNO 1
PROCNO 1
Date_ 20170910
Time 14.52
INSTRUM spect
PROBHD 5 mm PABBO BB/
PULPROG zg30
TD 65536
SOLVENT CDCl3
NS 8
DS 0
SWH 8012.820 Hz
FIDRES 0.122266 Hz
AQ 4.0894966 sec
RG 31.52
DW 62.400 usec
DE 6.50 usec
TE 297.0 K
D1 1.00000000 sec
TD0 1
======== CHANNEL f1 ========
SFO1 400.0322002 MHz
NUC1 1H
P1 10.00 usec
SI 65536
SF 400.0300042 MHz
WDW EM
SSB 0
LB 0.00 Hz
GB 0
PC 1.00
9
8
7
6
5
4
3
2
1
0
ppm
1.00
1.01
1.94
1.08
0.99
1.00
1.09
1.04
3.08
3.10

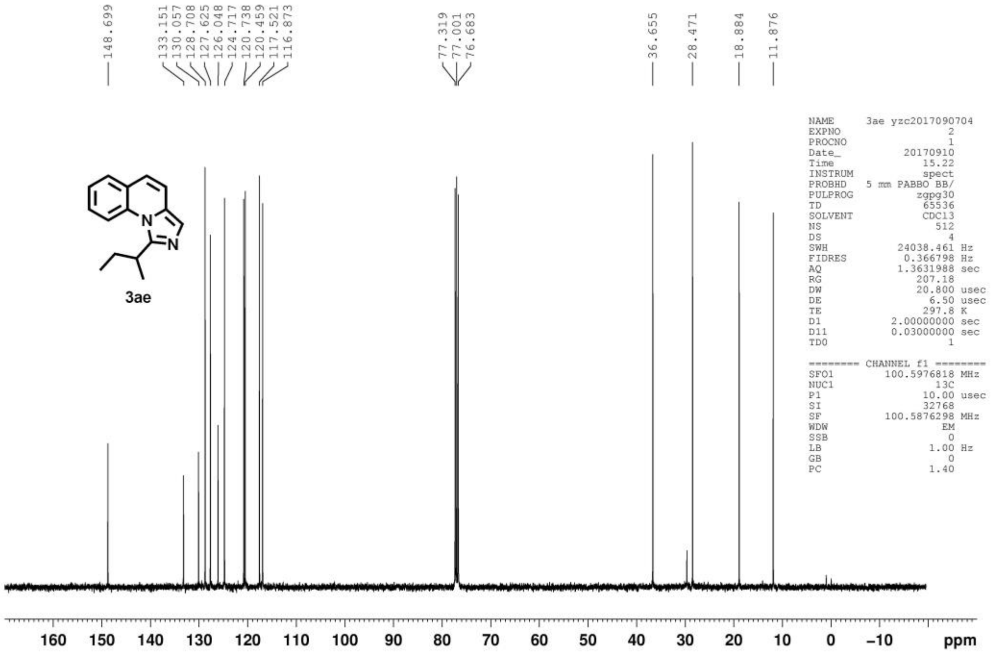
148.699
133.151
130.057
128.708
127.625
126.048
124.717
120.738
120.459
117.521
116.873
77.319
77.001
76.683
36.655
28.471
18.884
11.876
3ae
NAME 3ae yzc2017090704
EXPNO 2
PROCNO 1
Date_ 20170910
Time 15.22
INSTRUM spect
PROBHD 5 mm PABBO BB/
PULPROG zgpg30
TD 65536
SOLVENT CDCl3
NS 512
DS 4
SWH 24038.461 Hz
FIDRES 0.366798 Hz
AQ 1.3631988 sec
RG 207.18
DW 20.800 usec
DE 6.50 usec
TE 297.8 K
D1 2.00000000 sec
D11 0.03000000 sec
TD0 1
======== CHANNEL f1 ========
SFO1 100.5976818 MHz
NUC1 13C
P1 10.00 usec
SI 32768
SF 100.5876298 MHz
WDW EM
SSB 0
LB 1.00 Hz
GB 0
PC 1.40
160
150
140
130
120
110
100
90
80
70
60
50
40
30
20
10
0
−10
ppm

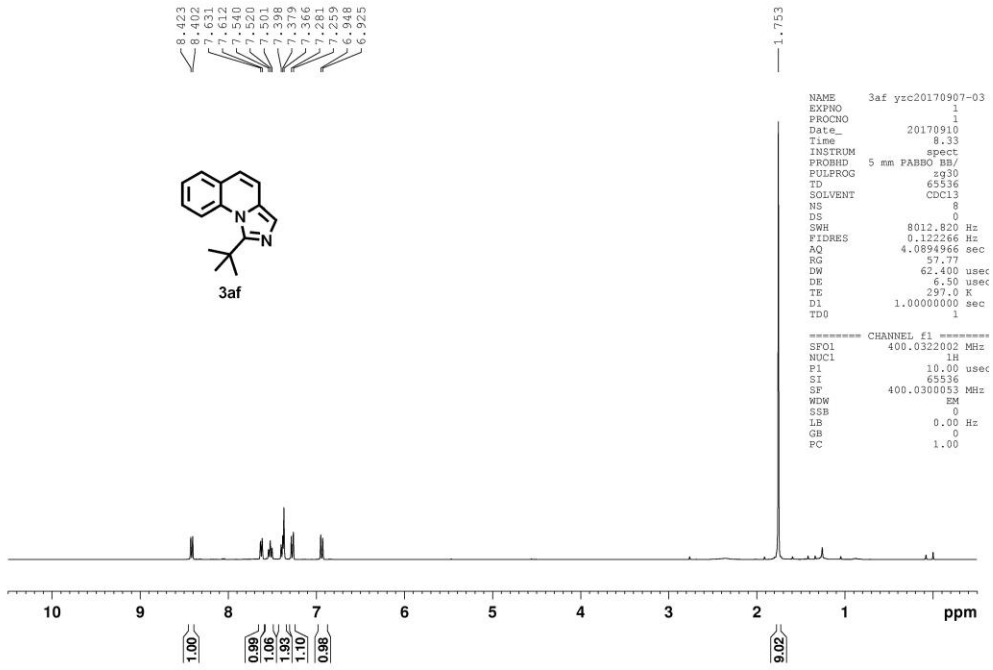
8.423
8.402
7.631
7.612
7.540
7.520
7.501
7.398
7.379
7.366
7.281
7.259
6.948
6.925
1.753
3af
NAME 3af yzc20170907-03
EXPNO 1
PROCNO 1
Date_ 20170910
Time 8.33
INSTRUM spect
PROBHD 5 mm PABBO BB/
PULPROG zg30
TD 65536
SOLVENT CDCl3
NS 8
DS 0
SWH 8012.820 Hz
FIDRES 0.122266 Hz
AQ 4.0894966 sec
RG 57.77
DW 62.400 usec
DE 6.50 usec
TE 297.0 K
D1 1.00000000 sec
TD0 1
======== CHANNEL f1 ========
SFO1 400.0322002 MHz
NUC1 1H
P1 10.00 usec
SI 65536
SF 400.0300053 MHz
WDW EM
SSB 0
LB 0.00 Hz
GB 0
PC 1.00
10
9
8
7
6
5
4
3
2
1
ppm
1.00
0.99
1.06
1.93
1.10
0.98
9.02

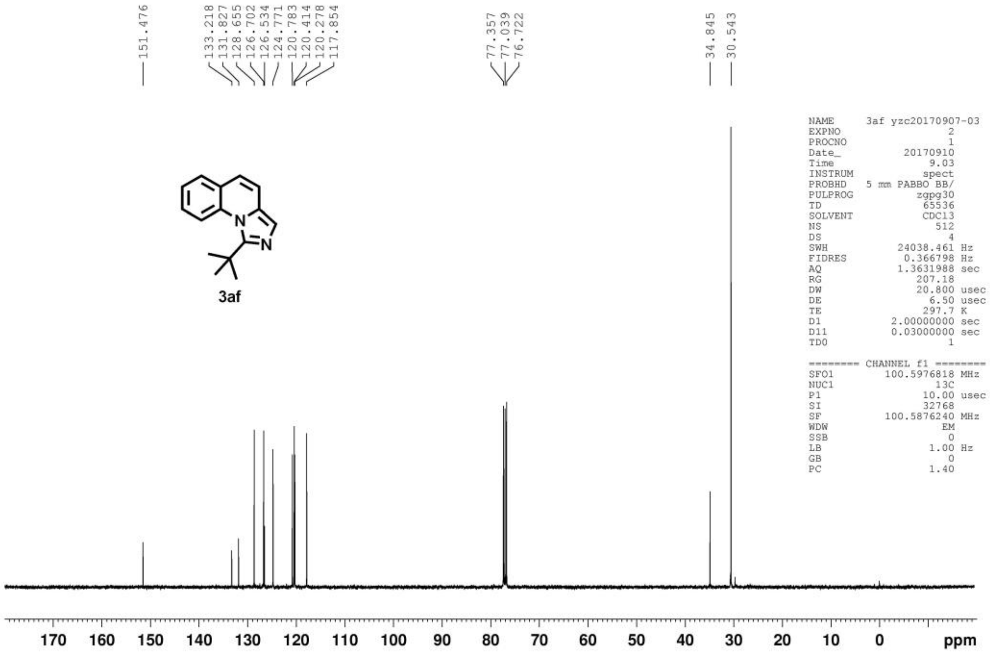
151.476
133.218
131.827
128.655
126.702
126.534
124.771
120.783
120.414
120.278
117.854
77.357
77.039
76.722
34.845
30.543
3af
NAME 3af yzc20170907-03
EXPNO 2
PROCNO 1
Date_ 20170910
Time 9.03
INSTRUM spect
PROBHD 5 mm PABBO BB/
PULPROG zgpg30
TD 65536
SOLVENT CDCl3
NS 512
DS 4
SWH 24038.461 Hz
FIDRES 0.366798 Hz
AQ 1.3631988 sec
RG 207.18
DW 20.800 usec
DE 6.50 usec
TE 297.7 K
D1 2.00000000 sec
D11 0.03000000 sec
TD0 1
======== CHANNEL f1 ========
SFO1 100.5976818 MHz
NUC1 13C
P1 10.00 usec
SI 32768
SF 100.5876240 MHz
WDW EM
SSB 0
LB 1.00 Hz
GB 0
PC 1.40
170
160
150
140
130
120
110
100
90
80
70
60
50
40
30
20
10
0
ppm

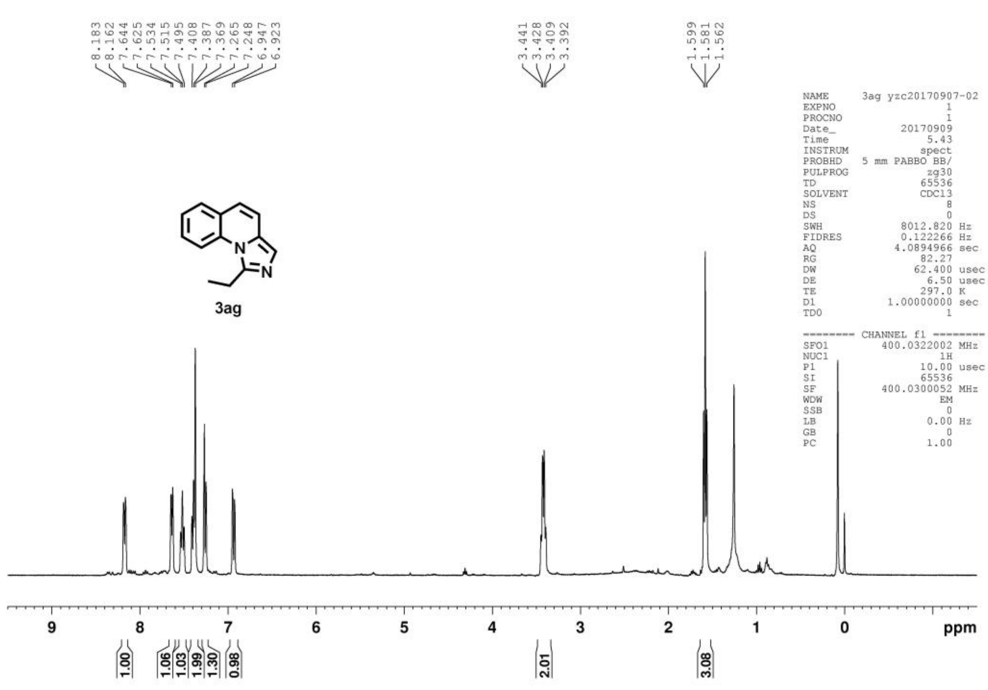

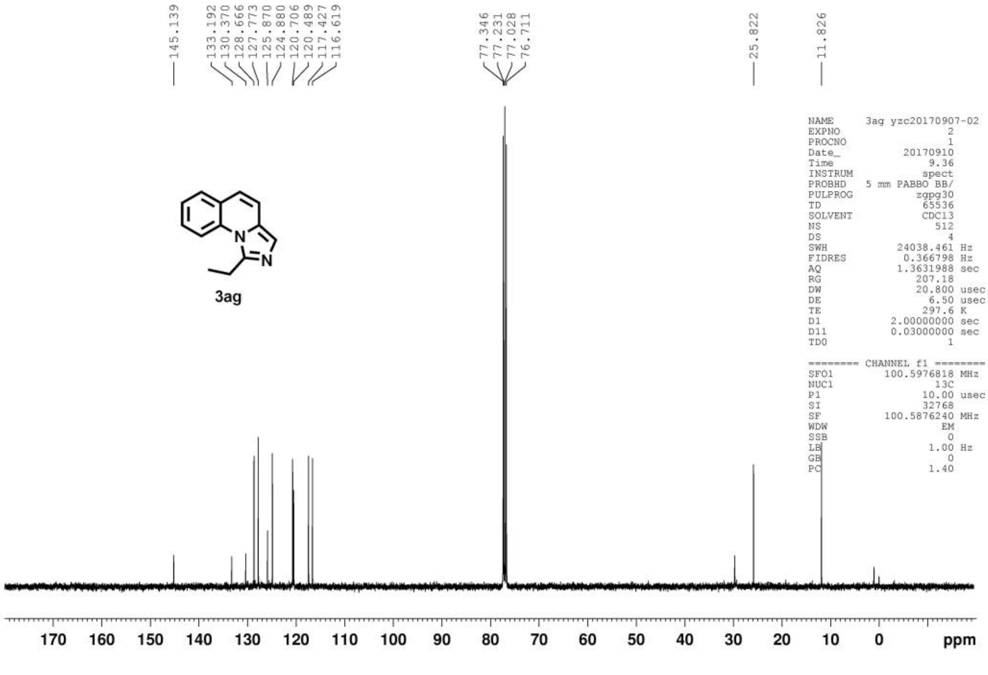

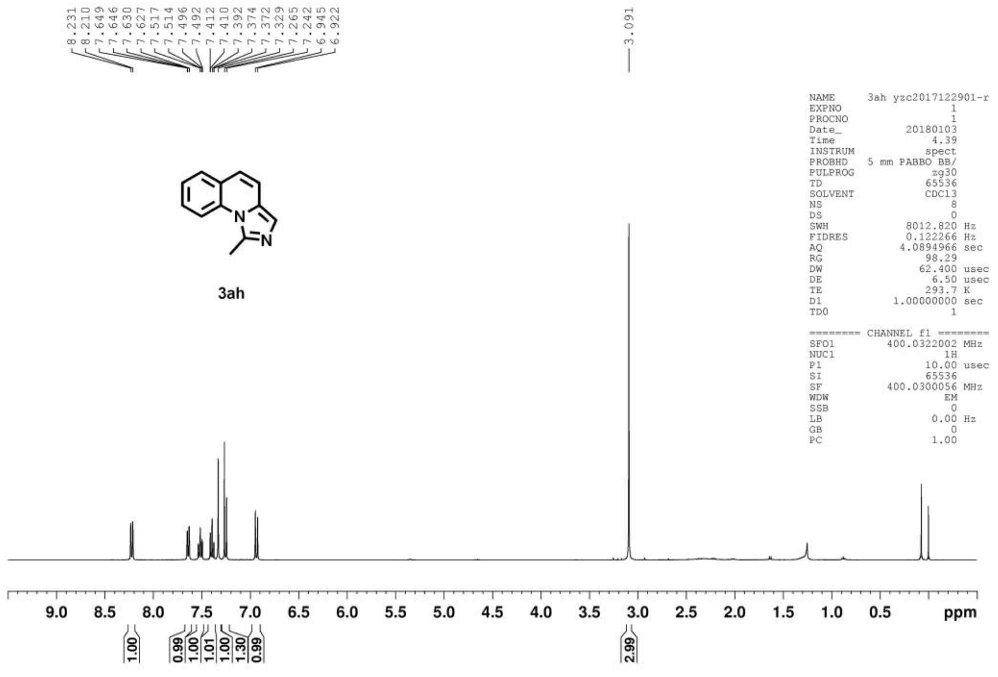
8.231 8.210 7.649 7.646 7.630 7.627 7.514 7.517 7.496 7.492 7.412 7.410 7.392 7.374 7.372 7.329 7.265 7.242 6.945 6.922
3.091
3ah
NAME 3ah yzc2017122901-r
EXPNO 1
PROCNO 1
Date_ 20180103
Time 4.39
INSTRUM spect
PROBHD 5 mm PABBO BB/
PULPROG zg30
TD 65536
SOLVENT CDCl3
NS 8
DS 0
SWH 8012.820 Hz
FIDRES 0.122266 Hz
AQ 4.0894966 sec
RG 98.29
DW 62.400 usec
DE 6.50 usec
TE 293.7 K
D1 1.00000000 sec
TD0 1
======== CHANNEL f1 ========
SFO1 400.0322002 MHz
NUC1 1H
P1 10.00 usec
SI 65536
SF 400.0300056 MHz
WDW EM
SSB 0
LB 0.00 Hz
GB 0
PC 1.00
9.0 8.5 8.0 7.5 7.0 6.5 6.0 5.5 5.0 4.5 4.0 3.5 3.0 2.5 2.0 1.5 1.0 0.5 ppm
1.00 0.99 1.00 1.01 1.00 1.30 0.99 2.99

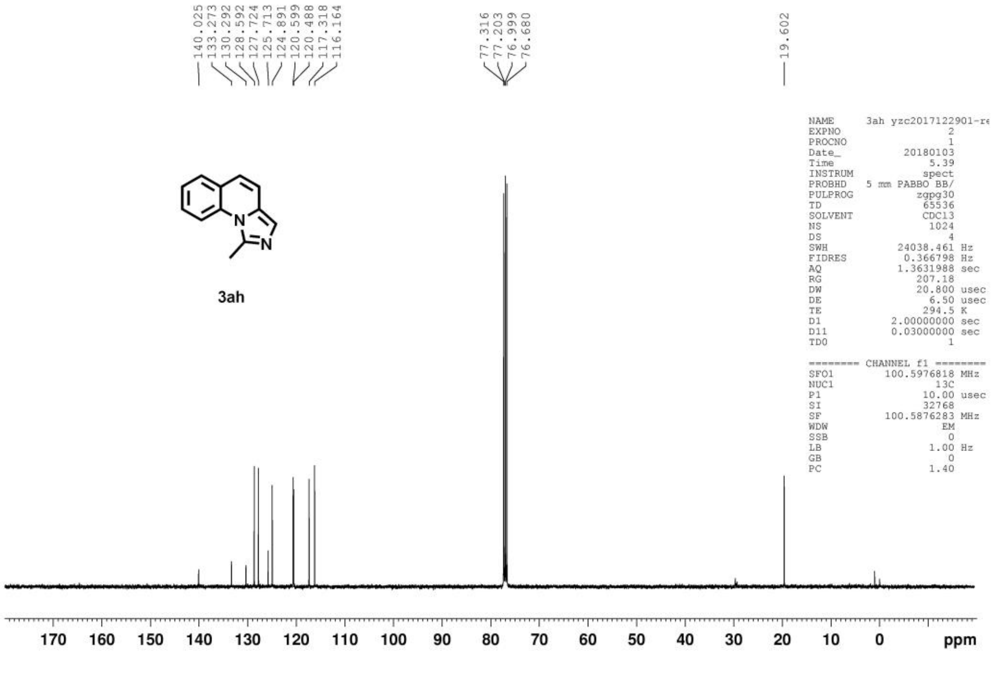
140.025 133.273 130.292 128.592 127.724 125.713 124.891 120.599 120.488 117.318 116.164
77.316 77.203 76.999 76.680
19.602
3ah
NAME 3ah yzc2017122901-re
EXPNO 2
PROCNO 1
Date_ 20180103
Time 5.39
INSTRUM spect
PROBHD 5 mm PABBO BB/
PULPROG zgpg30
TD 65536
SOLVENT CDCl3
NS 1024
DS 4
SWH 24038.461 Hz
FIDRES 0.366798 Hz
AQ 1.3631988 sec
RG 207.18
DW 20.800 usec
DE 6.50 usec
TE 294.5 K
D1 2.00000000 sec
D11 0.03000000 sec
TD0 1
======== CHANNEL f1 ========
SFO1 100.5976818 MHz
NUC1 13C
P1 10.00 usec
SI 32768
SF 100.5876283 MHz
WDW EM
SSB 0
LB 1.00 Hz
GB 0
PC 1.40
170 160 150 140 130 120 110 100 90 80 70 60 50 40 30 20 10 0 ppm

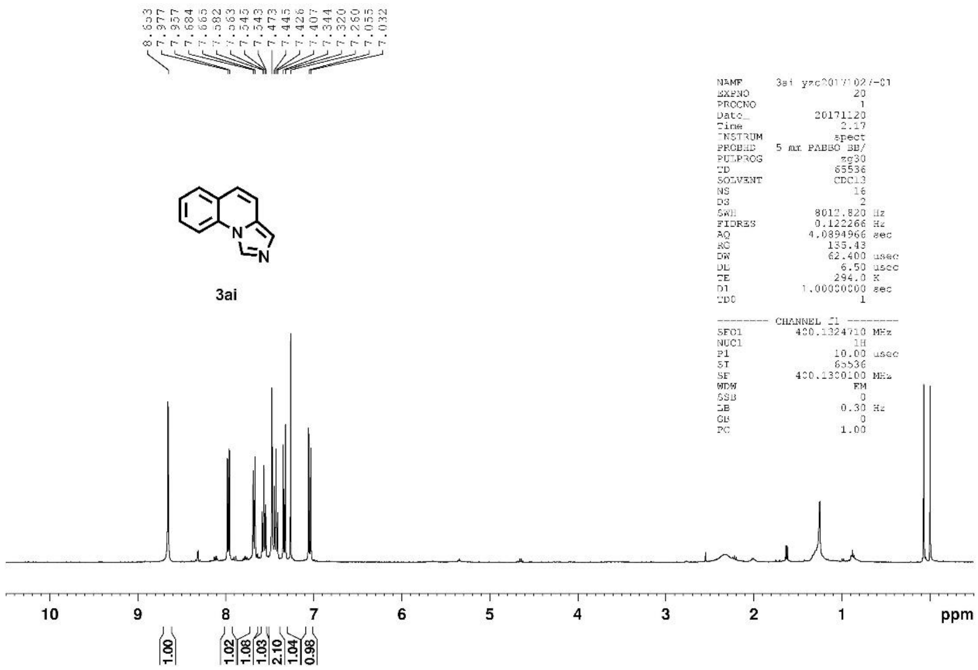
8.653
7.977
7.957
7.684
7.665
7.582
7.563
7.545
7.543
7.473
7.446
7.426
7.407
7.344
7.320
7.260
7.055
7.032
3ai
NAME 3ai yzc20171027-01
EXPNO 20
PROCNO 1
Date_ 20171120
Time 2.17
INSTRUM spect
PROBHD 5 mm PABBO BB/
PULPROG zg30
TD 65536
SOLVENT CDCl3
NS 16
DS 2
SWH 8012.820 Hz
FIDRES 0.122266 Hz
AQ 4.0894966 sec
RG 135.43
DW 62.400 usec
DE 6.50 usec
TE 294.0 K
D1 1.00000000 sec
TD0 1
======== CHANNEL f1 ========
SFO1 400.1324710 MHz
NUC1 1H
P1 10.00 usec
SI 65536
SF 400.1300100 MHz
WDW EM
SSB 0
LB 0.30 Hz
GB 0
PC 1.00
10
9
8
7
6
5
4
3
2
1
ppm
1.00
1.02
1.08
1.03
2.10
1.04
0.98

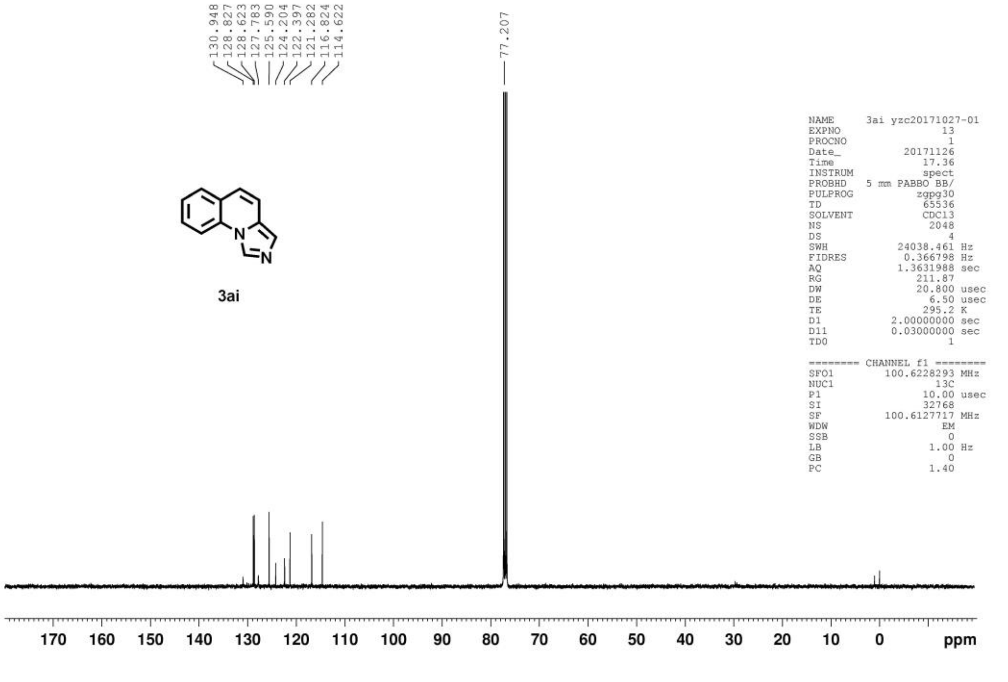
130.948
128.827
128.623
127.783
125.590
124.204
122.397
121.282
116.824
114.622
77.207
3ai
NAME 3ai yzc20171027-01
EXPNO 13
PROCNO 1
Date_ 20171126
Time 17.36
INSTRUM spect
PROBHD 5 mm PABBO BB/
PULPROG zgpg30
TD 65536
SOLVENT CDCl3
NS 2048
DS 4
SWH 24038.461 Hz
FIDRES 0.366798 Hz
AQ 1.3631988 sec
RG 211.87
DW 20.800 usec
DE 6.50 usec
TE 295.2 K
D1 2.00000000 sec
D11 0.03000000 sec
TD0 1
======== CHANNEL f1 ========
SFO1 100.6228293 MHz
NUC1 13C
P1 10.00 usec
SI 32768
SF 100.6127717 MHz
WDW EM
SSB 0
LB 1.00 Hz
GB 0
PC 1.40
170
160
150
140
130
120
110
100
90
80
70
60
50
40
30
20
10
0
ppm

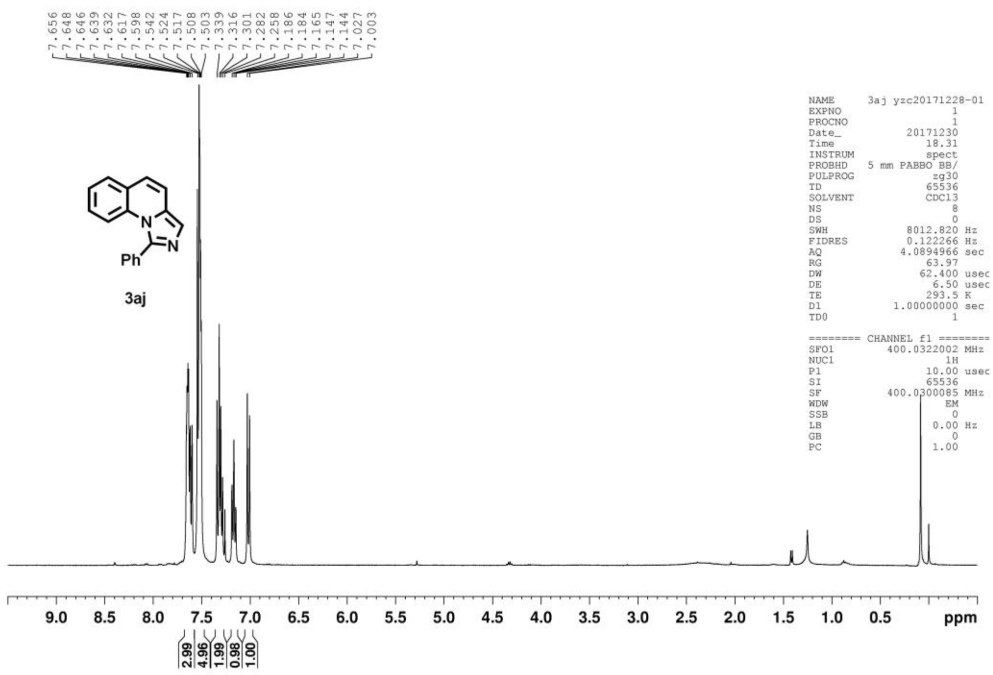
7.656
7.648
7.646
7.639
7.632
7.617
7.598
7.542
7.524
7.517
7.508
7.503
7.339
7.316
7.301
7.282
7.258
7.186
7.184
7.165
7.147
7.144
7.027
7.003
Ph
N
N
3aj
NAME 3aj yzc20171228-01
EXPNO 1
PROCNO 1
Date_ 20171230
Time 18.31
INSTRUM spect
PROBHD 5 mm PABBO BB/
PULPROG zg30
TD 65536
SOLVENT CDCl3
NS 8
DS 0
SWH 8012.820 Hz
FIDRES 0.122266 Hz
AQ 4.0894966 sec
RG 63.97
DW 62.400 usec
DE 6.50 usec
TE 293.5 K
D1 1.00000000 sec
TD0 1
======== CHANNEL f1 ========
SFO1 400.0322002 MHz
NUC1 1H
P1 10.00 usec
SI 65536
SF 400.0300085 MHz
WDW EM
SSB 0
LB 0.00 Hz
GB 0
PC 1.00
9.0 8.5 8.0 7.5 7.0 6.5 6.0 5.5 5.0 4.5 4.0 3.5 3.0 2.5 2.0 1.5 1.0 0.5 ppm
2.99
4.96
1.99
0.98
1.00

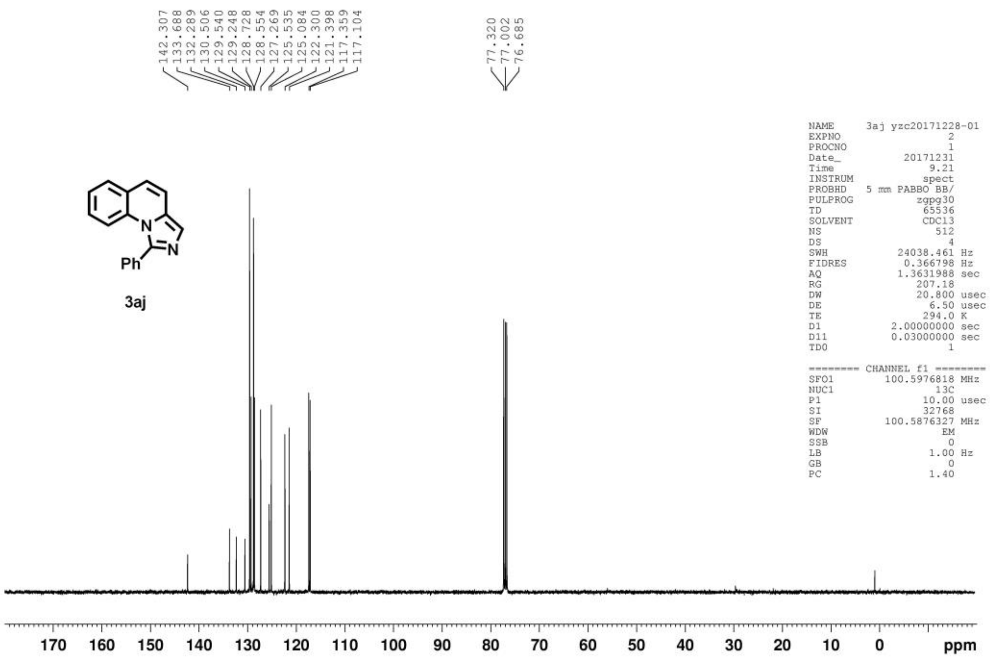
142.307
133.688
132.289
130.506
129.540
129.248
128.728
128.554
127.269
125.535
125.084
122.300
121.398
117.359
117.104
77.320
77.002
76.685
Ph
N
N
3aj
NAME 3aj yzc20171228-01
EXPNO 2
PROCNO 1
Date_ 20171231
Time 9.21
INSTRUM spect
PROBHD 5 mm PABBO BB/
PULPROG zgpg30
TD 65536
SOLVENT CDCl3
NS 512
DS 4
SWH 24038.461 Hz
FIDRES 0.366798 Hz
AQ 1.3631988 sec
RG 207.18
DW 20.800 usec
DE 6.50 usec
TE 294.0 K
D1 2.00000000 sec
D11 0.03000000 sec
TD0 1
======== CHANNEL f1 ========
SFO1 100.5976818 MHz
NUC1 13C
P1 10.00 usec
SI 32768
SF 100.5876327 MHz
WDW EM
SSB 0
LB 1.00 Hz
GB 0
PC 1.40
170 160 150 140 130 120 110 100 90 80 70 60 50 40 30 20 10 0 ppm

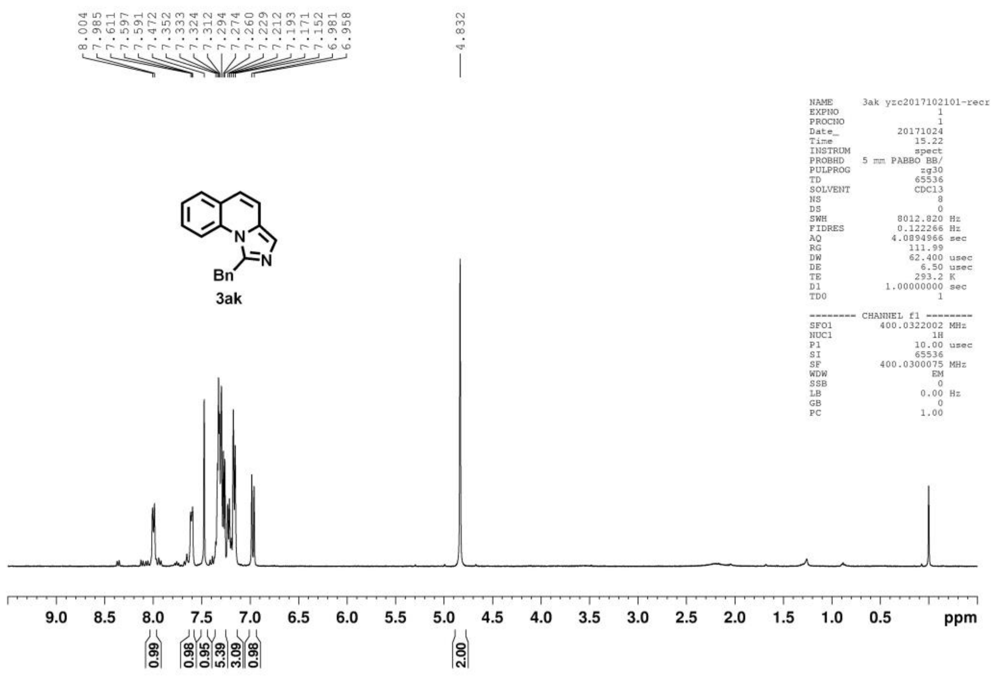
8.004
7.985
7.611
7.597
7.591
7.472
7.352
7.333
7.324
7.312
7.294
7.274
7.260
7.229
7.212
7.193
7.171
7.152
6.981
6.958
4.832
Bn
N
N
3ak
NAME 3ak yzc2017102101-recr
EXPNO 1
PROCNO 1
Date_ 20171024
Time 15.22
INSTRUM spect
PROBHD 5 mm PABBO BB/
PULPROG zg30
TD 65536
SOLVENT CDCl3
NS 8
DS 0
SWH 8012.820 Hz
FIDRES 0.122266 Hz
AQ 4.0894966 sec
RG 111.99
DW 62.400 usec
DE 6.50 usec
TE 293.2 K
D1 1.00000000 sec
TD0 1
======== CHANNEL f1 ========
SFO1 400.0322002 MHz
NUC1 1H
P1 10.00 usec
SI 65536
SF 400.0300075 MHz
WDW EM
SSB 0
LB 0.00 Hz
GB 0
PC 1.00
9.0 8.5 8.0 7.5 7.0 6.5 6.0 5.5 5.0 4.5 4.0 3.5 3.0 2.5 2.0 1.5 1.0 0.5 ppm
0.99
0.98
0.95
5.39
3.09
0.98
2.00

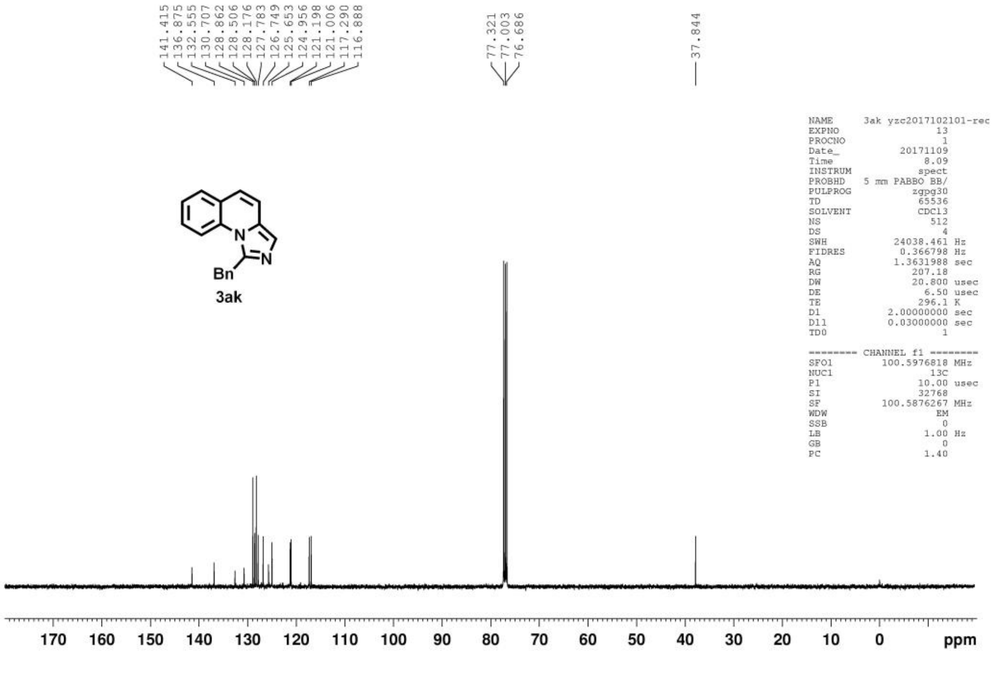
141.415
136.875
132.555
130.707
128.862
128.506
128.176
127.783
126.749
125.653
124.956
121.198
121.006
117.290
116.888
77.321
77.003
76.686
37.844
Bn
N
N
3ak
NAME 3ak yzc2017102101-rec
EXPNO 13
PROCNO 1
Date_ 20171109
Time 8.09
INSTRUM spect
PROBHD 5 mm PABBO BB/
PULPROG zgpg30
TD 65536
SOLVENT CDCl3
NS 512
DS 4
SWH 24038.461 Hz
FIDRES 0.366798 Hz
AQ 1.3631988 sec
RG 207.18
DW 20.800 usec
DE 6.50 usec
TE 296.1 K
D1 2.00000000 sec
D11 0.03000000 sec
TD0 1
======== CHANNEL f1 ========
SFO1 100.5976818 MHz
NUC1 13C
P1 10.00 usec
SI 32768
SF 100.5876267 MHz
WDW EM
SSB 0
LB 1.00 Hz
GB 0
PC 1.40
170 160 150 140 130 120 110 100 90 80 70 60 50 40 30 20 10 0 ppm

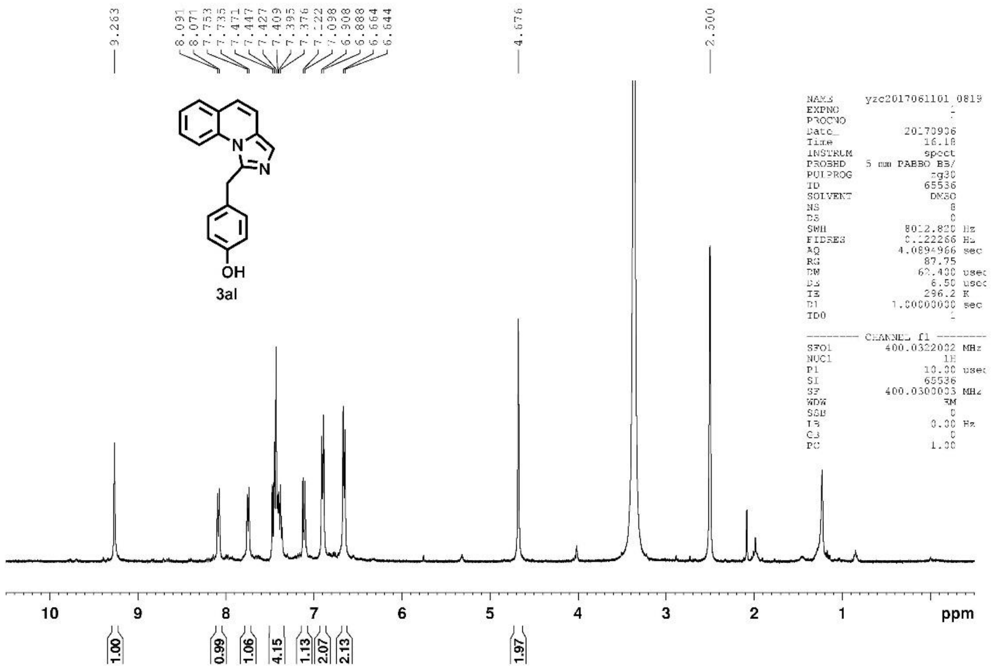
9.263
8.091
8.071
7.753
7.735
7.471
7.447
7.427
7.409
7.395
7.376
7.122
7.098
6.908
6.888
6.664
6.644
4.676
2.500
N
N
OH
3al
10
9
8
7
6
5
4
3
2
1
ppm
1.00
0.99
1.06
4.15
1.13
2.07
2.13
1.97

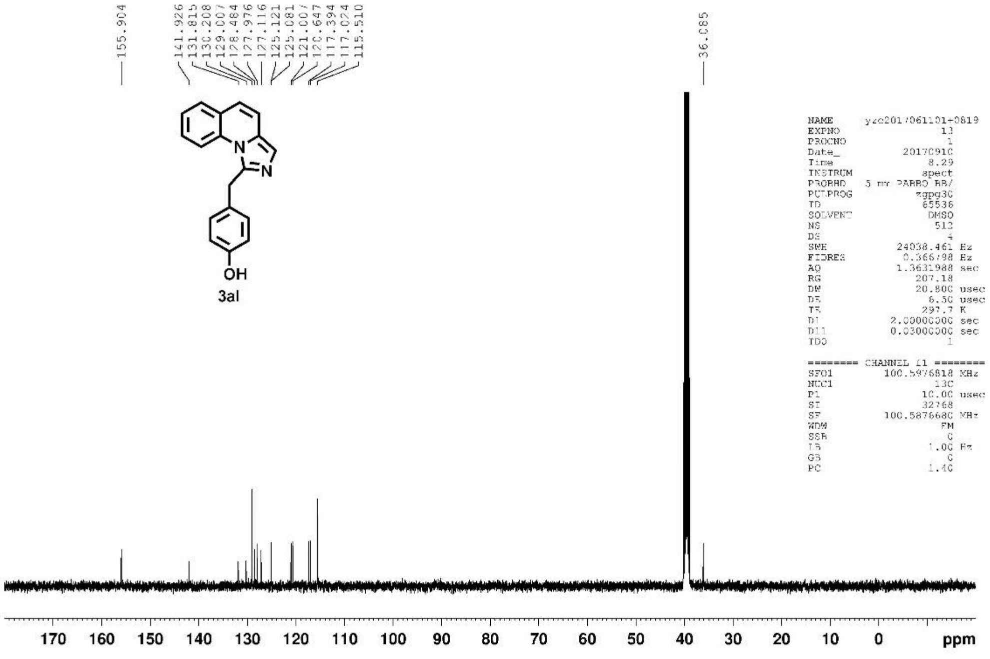
155.904
141.926
131.815
130.208
129.007
128.484
127.976
127.116
125.121
125.081
121.007
120.647
117.394
117.024
115.510
36.085
N
N
OH
3al
170
160
150
140
130
120
110
100
90
80
70
60
50
40
30
20
10
0
ppm
